# eBird Data Highlight Shifts in Wetland Resources Structuring Waterfowl and Shorebird Abundance

**DOI:** 10.1002/ece3.73061

**Published:** 2026-02-17

**Authors:** J. Patrick Donnelly, Johnnie N. Moore, John S. Kimball, Shea Coons, Daniel P. Collins, Mark J. Petri, David E. Naugle

**Affiliations:** ^1^ Ducks Unlimited Inc. Missoula Montana USA; ^2^ W.A. Franke College of Forestry and Conservation University of Montana Missoula Montana USA; ^3^ Numerical Terradynamic Simulation Group University of Montana Missoula Montana USA; ^4^ Group for Quantitative Study of Snow and Ice, Department of Geosciences University of Montana Missoula Montana USA; ^5^ Borderlands Research Institute Sul Ross State University Alpine Texas USA; ^6^ Ducks Unlimited, Inc. Vancouver Washington USA

**Keywords:** flyways, shorebirds, waterfowl, western United States, wetlands

## Abstract

Recent findings documenting rapid drying in some wetland ecosystems raise concerns over the sustainability of flyway habitats supporting migratory waterbirds. To improve our understanding of these potential impacts, we combined newly available data documenting 40 years (1984–2023) of wetland surface water hydrology in the western U.S. with eBird relative abundance maps to identify emerging bottlenecks in habitat availability. Assessments were made using an ensemble of shorebird and waterfowl species (hereafter waterbirds) representing diverse life histories tied to wetland ecosystems within the region. A machine‐learning approach was applied to identify wetland factors important to structuring individual species abundance as a spatial framework to assess changes in essential ecosystem functions aligned with seasonal distributions (i.e., breeding, post‐breeding migration, nonbreeding, pre‐breeding migration). Inundated wetlands accounted for only 0.3% of land cover within the study area. “Wetland area” (measured as surface water extent), semi‐permanent wetlands, and littoral saline lake wetlands were the primary factors structuring bird abundance. Waterbirds exhibited patterns of density dependence to offset resource scarcity by aggregating within landscapes encompassing the most predictable and abundant wetland habitats. Functional wetland losses, caused by persistent declines in surface water, overlapped with species' annual cycles signaling decreasing availability and greater uncertainty in waterbird habitats. Losses were highest in semi‐permanent wetlands, with declines of 19%–48% over the past 20 years. While these effects were based on a selection of representative species, impacts were emblematic of associated waterbird guilds reliant on concurrent wetland environments. To address rapid change in wetland resources, we encourage integrating our approach into waterbird management strategies to conserve the ecological processes that support flyway function in North America and worldwide.

## Graphical abstract

Wetland losses in the American West, driven by accelerated drying, overlapped multiple shorebird and waterfowl life histories. Trends were indicators of declining availability and increased uncertainty in waterbird habitat networks. Losses were pervasive in semi‐permanent wetlands, with 19%–48% reductions over the past two decades. While these effects were derived using a selection of wasterfowl and shorebird species, impacts were representative of waterbird guilds reliant on similar wetland environments.

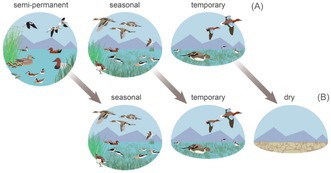

## 1 Introduction

Conservation of migratory bird flyways is complex, requiring knowledge of species movements and landscape interactions between distinct geographic regions spanning hundreds to thousands of kilometers that collectively support breeding, migration, and nonbreeding habitats. Climate and land‐use change have substantially increased the risk of species declines globally (Spooner et al. 2018). Migratory birds are especially vulnerable to these changes because their annual life cycles depend on vast geographic ranges, which can expose populations to multiple independent risks (Zurell et al. 2018). Risks are compounded by cross‐seasonal effects where environmental conditions experienced in one location (e.g., breeding grounds or migration) can affect fitness in subsequent locations, leading to declines in long‐term demographic performance (sensu Sedinger and Alisauskas 2014). While some birds have changed their migration chronology and range extent to align with shifting climate and land‐use patterns (Hitch and Leberg 2007; Tomotani et al. 2018), increasing environmental pressures are likely to outstrip the adaptive plasticity of many species (Schmaljohann and Both 2017).

In the arid and semi‐arid western United States (U.S.), migratory shorebirds (Charadriiformes) and waterfowl (Anatidae) rely on wetland landscapes that function as nodes in broader continental habitat networks (i.e., flyways). Systematic monitoring of wetland change has focused on historical threats driven by land conversion from agricultural and urban expansion (Dahl et al. 1991) that have reported a cumulative decline of only 0.6% to freshwater palustrine systems since 1986 (Dahl 2000, 2006, 2011; Lang et al. 2024). Recent efforts focused on indirect ecological effects (rather than direct human impacts) have revealed systemic shifts to wetland function driven by increased water scarcity over the past two decades (Donnelly et al. 2025). Patterns show overall wetland losses exceeding 20% in some regions of the western U.S., with most systems transitioning towards more pronounced and less predictable ephemeral states due to accelerated wetland drying. New information raises concerns over the resilience of migratory flyways as rapid wetland change risks the emergence of resource bottlenecks driven by increasing misalignment between waterbirds and their annual life history needs.

Detailed knowledge of migratory waterbird distribution and abundance has curtailed evaluations of changing flyway conditions. The ongoing evolution of miniaturized GPS tracking devices has provided valuable insight for some species; however, widespread use has been constrained by birds large enough to carry tags and costs to purchase and deploy the technology (Geen et al. 2019). Innovations by the citizen science platform eBird (Sullivan et al. 2009) have recently changed this paradigm through the creation of spatially explicit abundance data depicting bird distributions by seasonal life histories (i.e., breeding, post‐breeding migration, nonbreeding, and pre‐breeding migration) for over 2400 species globally (Johnston et al. 2020). eBird data has been shown to function as an accurate surrogate for traditional surveys when used to depict temporal distributions of bird abundance at local (Feng and Che‐Castaldo 2021) and regional levels (Fink et al. 2020; Horns et al. 2018; Walker and Taylor 2017). Integration of this information into conservation design has filled crucial gaps in evaluating spatially explicit interactions between migratory birds and large‐scale habitat networks supporting populations (sensu Stuber et al. 2022).

In the western U.S., rapid drying of wetland ecosystems threatens the sustainability of wetland habitat networks that support continental waterbird flyways. Our understanding of these impacts remains limited because large‐scale monitoring efforts (Dahl 2000, 2006, 2011; Lang et al. 2024) inventory the presence or absence of sites rather than changes in hydrological processes that influence their value and availability to waterbirds. To address this knowledge gap, we combined new data from Donnelly et al. (2025), which document 40 years (1984–2023) of wetland surface‐water hydrology in the western U.S., with eBird (2008–2022) relative abundance maps to identify emerging bottlenecks in waterbird habitat networks. Assessments utilized an ensemble of waterfowl and shorebird species, hereafter “waterbirds,” representing diverse life histories tied to wetland ecosystems within the western U.S. A machine‐learning approach was employed to identify wetland factors important in structuring bird abundance, creating a spatial and temporal framework to evaluate changes in wetland functions and their alignment with seasonal waterbird distributions (breeding, post‐breeding migration, nonbreeding, and pre‐breeding migration). Wetland factors influencing eBird abundance were then analyzed to identify habitat trends over the 40 years period (1984–2023). This work builds on similar regional analyses by Donnelly et al. (2020, 2022) and expands the scope to include 11 western states. The study results offer insights into the magnitude of waterbird habitat change and highlight the need for innovative conservation solutions that preserve the ecological processes supporting flyway function.

## 2 Methods

### 2.1 Study Area

**FIGURE 1:**
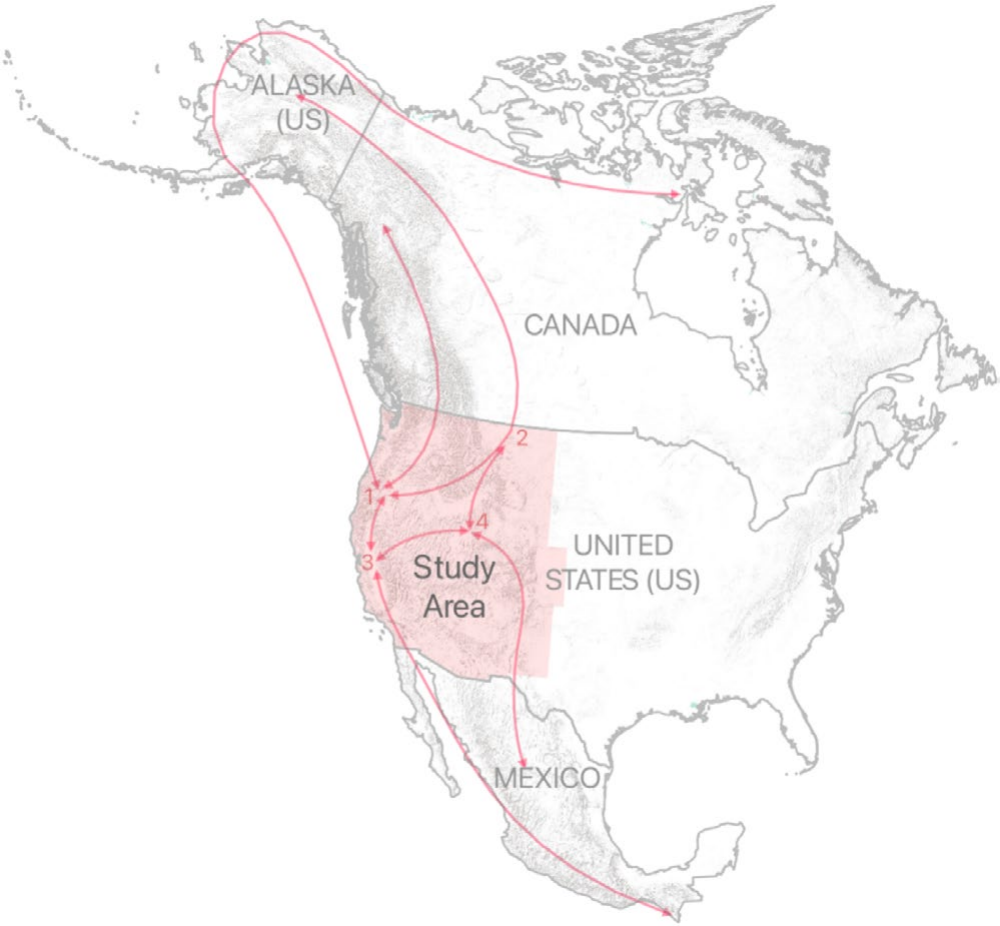
Study area shown in the context of generalized western North American shorebird and waterfowl flyways (red lines), including wetland landscapes of (1) Southern Oregon‐Northeast California (SONEC), (2) western Prairie Pothole region, (3) California's Central Valley/Bay‐Delta region, and (4) Utah's Great Salt Lake.

The study area consisted of 11 states in the western U.S., featuring landscapes with high wetland density and closed‐basin saline lakes recognized for their international and hemispheric importance in supporting North American waterbird flyways (North American Waterfowl Management Plan [NAWMP] et al. 2012; Figure 1; Senner et al. 2016). Key areas included California's Central Valley and Bay‐Delta region, Southern Oregon‐Northeast California (SONEC), Utah's Great Salt Lake, and the western parts of the Prairie Pothole Region. Wetland ecology was defined by geographically distinct hydrologic processes. Most wetlands occur naturally, but some are managed directly through irrigation infrastructure and water‐level manipulation (hereafter, “managed wetlands”). Managed wetlands are primarily located on state and federal wildlife refuges to sustain wetland habitats vital for migratory waterbirds. Other managed wetlands are maintained by private organizations for waterfowl hunting and shorebird conservation. Most of these sites were concentrated in California's Central Valley and around Utah's Great Salt Lake, supplying up to 60% of the wetland resources in those areas (United States Fish and Wildlife Service [USFWS] 2020). Estuarine wetlands were relatively limited and mainly found along coastlines, occurring in low‐energy bays at the terminus of riverine valleys (e.g., the Bay‐Delta region of California).

Agroecological systems in the study area account for half of the wetland resources (Donnelly et al. 2025). Although most agricultural practices fall outside traditional wetland definitions (sensu Cowardin et al. 1979), we included grass‐hay pasture (hereafter “grass‐hay‐wetlands”), rice cultivation (hereafter “rice”), and earthen stock ponds (hereafter “ponds”) in our analysis to evaluate their potential role in supporting migratory waterbird populations. For instance, grass‐hay grown for livestock forage in the Intermountain West supports over 300 kha of wetlands through flood irrigation on riparian floodplains (Donnelly, Jensco, et al. 2024). This practice has been linked to greater sandhill cranes (Antigone canadensis tabida), supporting 60% of their wetland breeding habitat in the western U.S. (Donnelly, Collins, et al. 2024). In California's Central Valley, rice cultivation supports one of the largest waterbird wintering areas in western North America, creating over 140 kha of agricultural habitat from November to February through post‐harvest flooding of fields to decompose leftover stubble (Petrie et al. 2016). Additionally, ponds built and maintained for livestock watering and local irrigation benefit waterfowl as surrogates for natural wetlands during breeding (Perez‐Arteaga et al. 2001) and migration (Mackell et al. 2021).

### 2.2 Waterbirds

**FIGURE 2:**
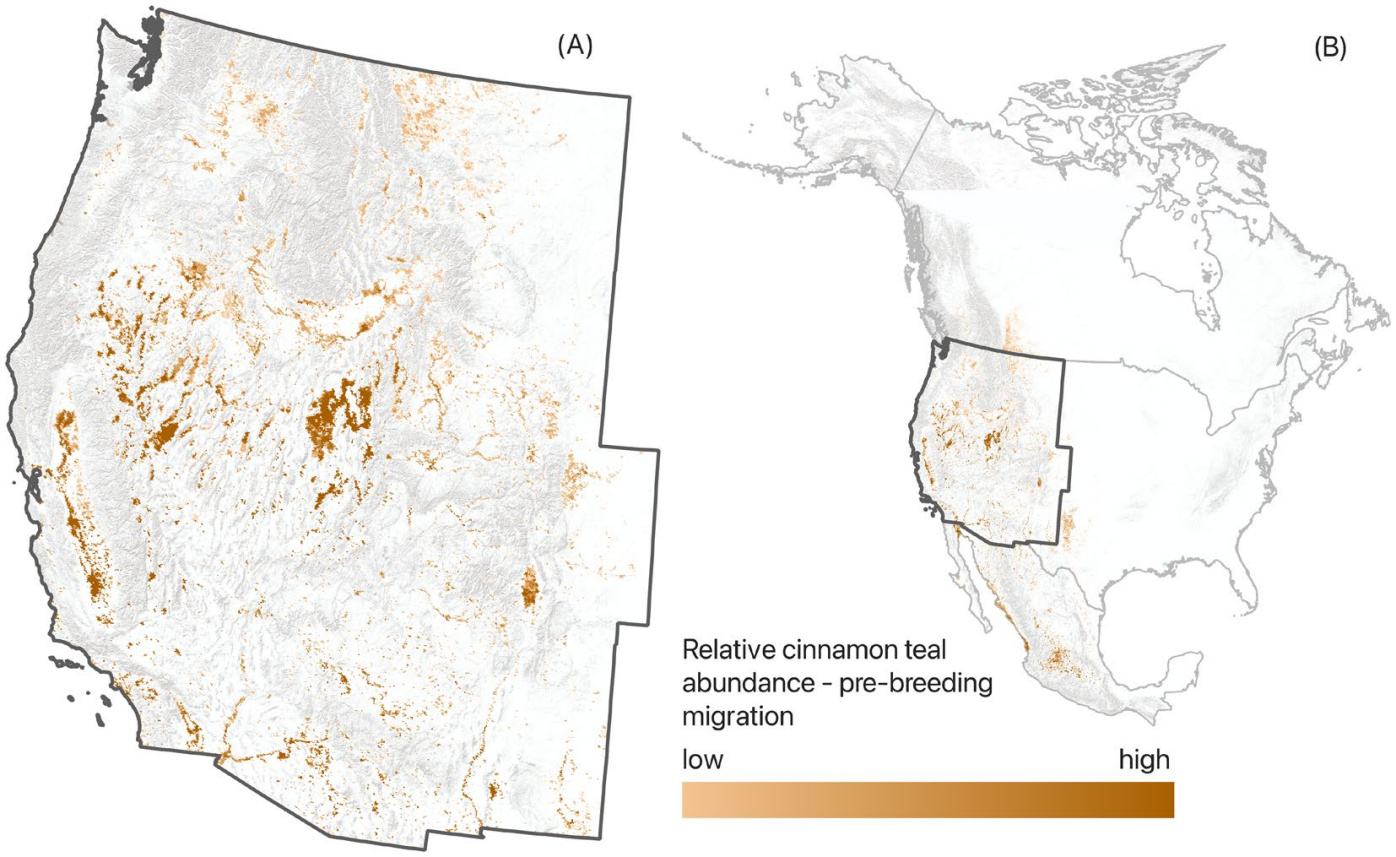
Example, eBird mean relative abundance distributions for cinnamon teal during pre‐breeding migration (February–April). Maps depict data for the study area (A) and North America (B). Data derived using eBird observations from 2008 to 2022.

A multi‐species approach representing associated dabbling duck, diving duck, and shorebird guilds was used to assess wetland change (Lambeck 1997). Selected species included American avocet ( *Recurvirostra americana* ), black‐necked stilts ( *Himantopus mexicanus* ), canvasbacks ( *Aythya valisineria* ), cinnamon teal ( *Anas cyanoptera* ), northern pintail ( *Anas acuta* ), and Wilson's phalarope ( *Phalaropus tricolor* ). Only species with 25% or more of their relative continental abundance in the western U.S. during breeding, post‐breeding migration, nonbreeding, or pre‐breeding migration periods (hereafter “annual cycles”) were included (Table 1). Proportional measures were estimated from North American eBird mean relative abundance maps (hereafter “abundance data”; Fink et al. 2023) generated from citizen field observations from 2008 to 2022 (Figure 2). Data were downloaded as raster layers (pixels = 2.5 × 2.5 km) and summarized using a zonal statistical function applied to the study area boundary. Results were used to interpret distribution patterns within annual cycles (as defined by eBrid; Fink et al. 2020) to ensure that selected species were representative of temporally and spatially diverse space‐use. For example, northern pintails broadly represented wetland resources associated with nonbreeding dabbling duck ecology, given the high concentrations of wintering birds in parts of the study area. In contrast, cinnamon teal winter at lower latitudes and breeds across the western U.S., providing a surrogate for examining wetland trends supporting breeding dabbling duck populations. Similar considerations were applied to diving duck and shorebird guilds. Other considerations included public support and funding for selected species, based on conservation status and socio‐economic and cultural values. For instance, northern pintails and canvasbacks are harvested species in long‐term decline that are emblematic of the funding strategies of hunting organizations (e.g., Ducks Unlimited) to support wetland conservation (sensu Bagstad et al. 2019). Similarly, American avocets, black‐necked stilts, and Wilson's phalaropes are frequently regarded as flagship species by shorebird conservation groups (e.g., Audubon and Manomet) for their value to the bird‐watching public (Senzaki et al. 2017). Annual cycle distribution maps for selected species are provided as Figures S1–S6.

**TABLE 1:** North American proportion of American avocet, black‐necked stilt, cinnamon teal, canvasback, northern pintail, and Wilson's phalarope populations in the western U.S.

	Breeding (%)	Post‐breeding migration (%)	Nonbreeding (%)	Pre‐breeding migration (%)
American avocet	58	85	10	73
Black‐necked stilt	49	50	13	34
Canvasback	3	10	28	8
Cinnamon teal	84	60	12	69
Northern pintail	1	19	34	10
Wilson's phalarope	90	81	0	17

For analysis, eBird annual cycle dates were rounded to the nearest month to align with the wetland trends data, which is summarized at monthly intervals. This truncated the breeding period of Black‐necked Stilts and Wilson's phalarope into a single month (Table 2). We assume these months to be representative of these species' core breeding periods.

**TABLE 2:** eBird annual cycles by species, rounded to the nearest month.

	Breeding	Post‐breeding migration	Nonbreeding	Pre‐breeding migration
American avocet	May–Jun	Jul–Nov	Dec–Jan	Feb–Apr
Black‐necked stilt	Jun	Jul–Nov	Dec–Feb	Mar–May
Canvasback	Jun–Aug	Sep–Nov	Dec–Jan	Feb–May
Cinnamon teal	May–Jun	Jul–Nov	Dec–Jan	Feb–Apr
Northern pintail	Jun–Jul	Aug–Nov	Dec–Jan	Feb–May
Wilson's phalarope	Jun	Jul–Oct	Nov–Feb	Mar–May

### 2.3 Wetland Hydroperiod

Wetland surface‐water hydrology in the western U.S. was measured to evaluate its role in predicting waterbird abundance. Using methods outlined by Donnelly et al. (2019), Landsat 5 Thematic Mapper and Landsat 8/9 Operational Land Imager satellite images were used to estimate monthly wetland surface water area for conditions from 2008 to 2022. These results aligned with the averaged eBird abundance data for the same period. Surface water estimates were classified by hydroperiod (i.e., the annual duration of wetland inundation). Hydroperiods are crucial for wetland functions, affecting food resources, plant communities, and habitat availability for waterbirds (Daniel and Rooney 2021). Hydroperiods were identified by summarizing monthly (January to December) surface water layers at the pixel level (30 × 30 m). Data were combined into a single raster layer by counting the number of months during which inundation was observed. Hydroperiod results were classified as “temporary” (flooded < 2 months), “seasonal” (flooded more than 2 but < 8 months), or “semi‐permanent” (flooded more than 8 months), following standards similar to Cowardin et al. (1979). The results provided an overall hydroperiod class layer representing average conditions from 2008 to 2022.

**FIGURE 3:**
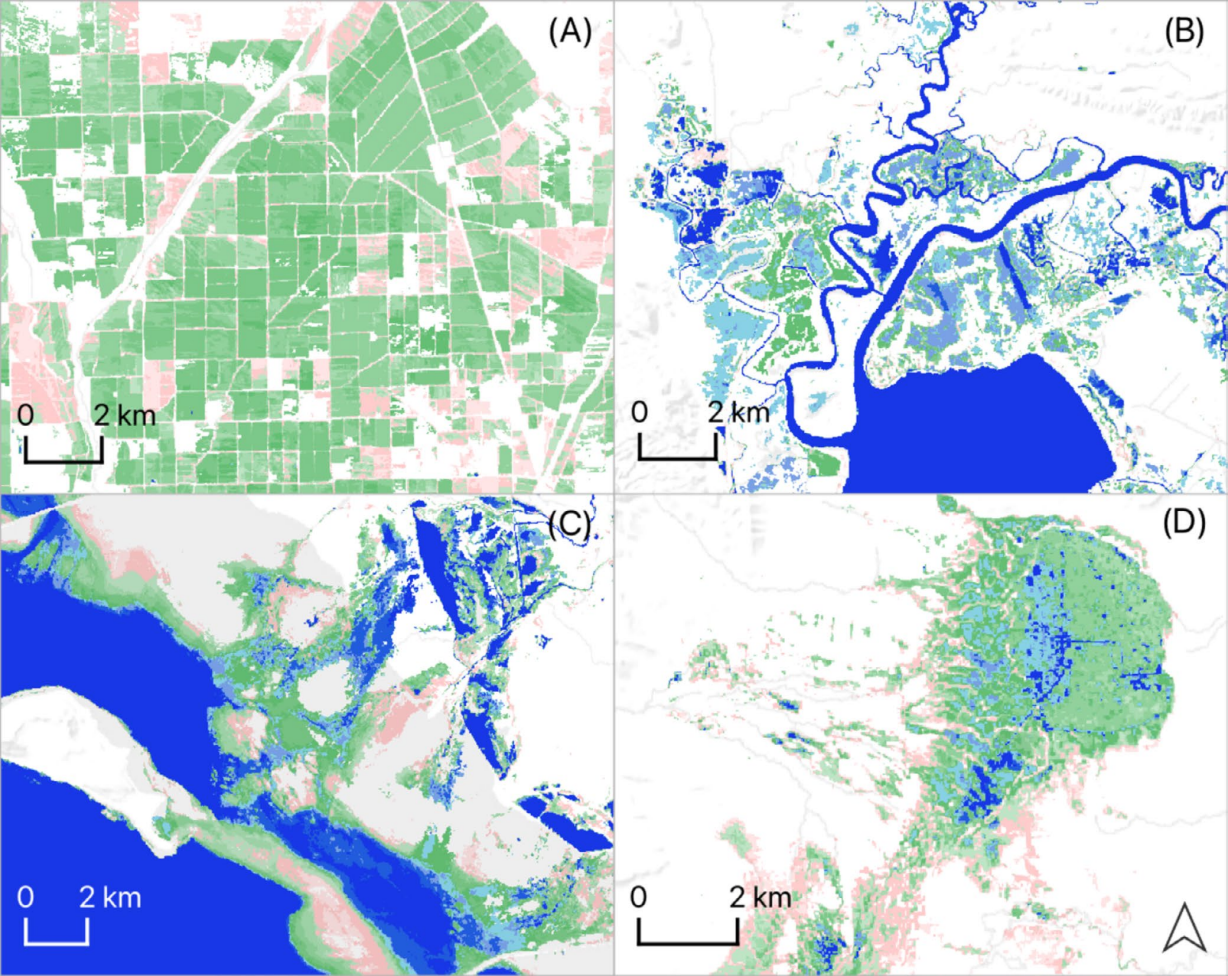
Wetland surface water and hydroperiod model examples: Flooded rice cultivation in Central Valley, California (A); estuarine and natural freshwater wetlands in California Bay‐Delta region (B); managed and littoral saline lake wetlands in Great Salt Lake (C); and natural wetlands in Sycan Marsh, Southern Oregon‐Northeast California (SONEC; D). Wetland surface water is classified by hydroperiod: Semi‐permanent (blue), seasonal (green), and temporary (pink).

To fit hydroperiod classes to waterbird species, wetland surface‐water measurements were repeated to estimate conditions during individual annual cycles (see Table 2). Results reflected average conditions from 2008 to 2022. Using a masking function, surface‐water extent for each species and annual cycle was applied to subset the previously generated 2008–2022 hydroperiod layer. These products provided spatially explicit representations of wetland surface water and hydroperiod conditions for individual waterbird species across their annual cycles, aligned with eBird abundance data for the same period (Figure 3). A more detailed description of the remote sensing methods used to generate wetland surface water estimates and classify hydroperiods is provided in the Appendix S2.

### 2.4 Functional Wetland Groups

We grouped wetlands into functional classes to explore additional factors influencing waterbird distributions (Table 3). These designations included rice, livestock/irrigation ponds, and grass‐hay wetlands as agroecological systems known to support habitat resources that sustain migratory waterbird populations (Fleskes et al. 2016; Fleskes and Battaglia 2004; Mackell et al. 2021). To classify wetland surface water and hydroperiod data, an exhaustive inventory of 780,000 polygons containing functional group designations from Donnelly et al. (2025) was used. Polygons were first converted into a raster layer and masked with surface‐water and hydroperiod layers for each species and annual cycle. The masked results were then added to the corresponding wetland surface‐water and hydroperiod data as an additional raster band containing pixel‐level functional group classifications. This allowed the use of spatial filtering to identify functional classes within wetland surface‐water and hydroperiod layers when associating results with species abundance data. Reservoirs and natural lakes were partially filtered by excluding areas classified as semi‐permanent hydroperiods. This removed perennial, deep water bodies while keeping temporary and seasonal wetlands supported by littoral lake fringes. A similar method was applied to lotic systems by removing semi‐permanent hydroperiods from the riparian functional group to exclude deepwater river channels while maintaining nearby temporary and seasonally flooded wetlands. Large reservoirs and lakes designated as wetland areas by Donnelly et al. (2025) were also included. These areas were mainly associated with river deltas and regions supporting emergent plant communities.

**TABLE 3:** Functional classes.

Class	Description
Estuary	Tidal saltwater wetlands
Grass‐hay‐wetlands	Wetland areas supported by flood‐irrigated grass‐hay production occurring in riparian floodplains as defined by Donnelly, Collins, et al. (2024), Donnelly, Jensco, et al. (2024)
Littoral (saline lake) wetlands	Littoral seasonal and temporary wetlands along the fringe of saline lakes (i.e., Great Salt Lake)
Littoral wetlands	Littoral seasonal and temporary wetlands along the fringe of natural freshwater lakes
Managed‐wetlands	Wetlands with hydrology controlled through the direct application and draining of water
Palustrine wetlands	Unmanaged, nonagricultural, nonlittoral freshwater palustrine wetlands
Pond	Small human‐made impoundments developed for watering livestock or for water storage used in local agricultural irrigation. Excludes urban water bodies, such as golf course ponds
Rice	Cultivated rice in the Central Valley of California flooded during the growing season (May–Aug) and from (Oct–Feb) for stubble decomposition

### 2.5 Classifying Variable Importance

We used randomForestSRC regression tree analysis (Ishwaran and Kogalur 2019) to assess the importance of wetland functional groups, wetland surface water, and hydroperiods in influencing waterbird abundance. Wetland layers were converted to densities using a sum function and a square 2.5 km kernel estimator for input into the randomForestSRC (Węglarczyk 2018). This method preserved the precision of finer‐scale wetland data (30 × 30 m) while rescaling pixel‐area values to match the spatial resolution of eBird abundance estimates (2.5 × 2.5 km). Wetland surface‐water layers were used as a variable representing the total wetland area (hereafter, “wetland area”). Cultivated rice was excluded from “wetland area” calculations to differentiate agroecological features from neighboring managed and natural wetland systems. All wetland covariate and bird abundance layers were merged at the pixel level using spatial join functions to generate tables for the randomForestSRC regression tree analysis.

The randomForest approach is a nonparametric measure of variable importance (herafter “VIMP”) that is less sensitive to collinearity issues among predictors and applies to ecological systems with non‐normal distributions (Culter et al. 2007; De'ath and Fabricius 2000). RandomForestSRC used a two‐step method of randomization to de‐correlate trees, which decreased variance and bias (Zhang and Lu 2012), resulting in more statistically powerful outcomes. Confidence intervals for importance measures were calculated for each variable in the model, using double bootstrap subsampling (*n* = 500, alpha = 0.05; Ishwaran and Lu 2019), to provide a quantitative comparison among relative importance results for each variable in the model. We used the Breiman‐Cutler (aka, permutation) method (Breiman 2002) to calculate VIMP for all wetland variables in each randomForest model. Hyperparameter tuning was used to identify the optimal number of trees needed (*n* ~ 1000–2000) to maximize model accuracy (Oshiro et al. 2012). Wetland VIMP scores were generated independently for each species' and their annual cycles (see Table 2). Results were presented as boxplots, arranged by VIMP score, to visualize differences in rankings, their overlaps, and the most important variables controlling waterbird abundance (Figures S7–S12). Nonbreeding distributions of Wilson's phalarope occurred outside the study area, preventing VIMP score generation for this period.

**FIGURE 4:**
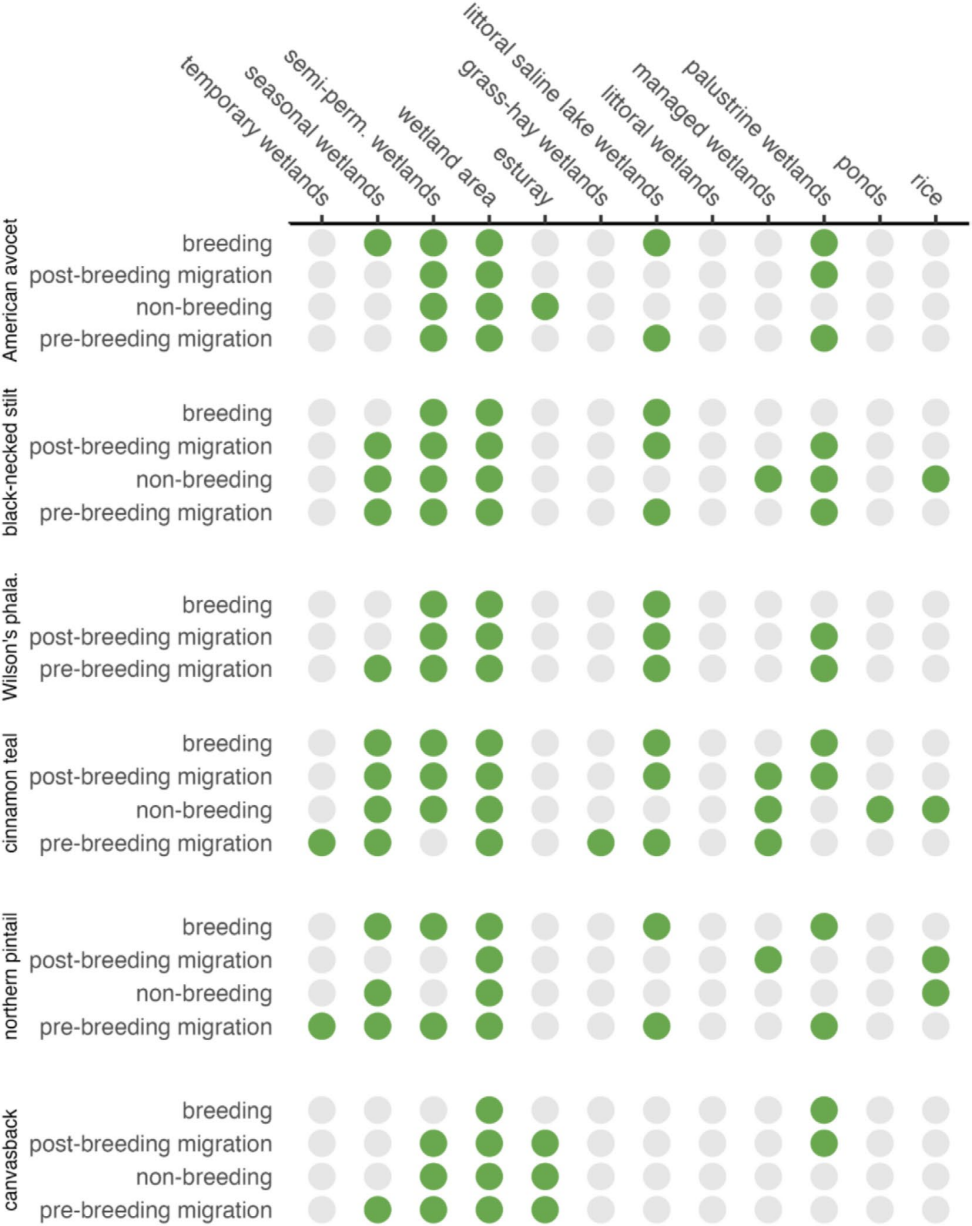
Wetland predictors of waterbird abundance in the western U.S. (x‐axis) by species' annual cycle (y‐axis). Features identified as important are shown in green, and features with limited influence in gray. Classification derived from RandomForestSRC variable importance scores.

We used the minimum depth to determine the most important wetland functional types associated with waterbird abundance. Minimum depth measures the average distance of a variable's splits from the root node. Variables that split near the root node tend to be more important, as they have a greater impact on the overall model structure. The mean of the distribution of minimum depths for all the trees in a model run is termed the “minimum depth threshold”. Minimum depth thresholds overcome the arbitrary classification of variable importance based on traditional techniques that identify a point along the ranking where there is a significant difference in VIMP rankings (Ishwaran et al. 2010). A simple optimal threshold rule was applied using the mean of the minimal depth distribution. Variables with minimal depth lower than this threshold were classified as important to waterbird abundance predictions (Figure 4). Variables above this threshold were considered to have a limited effect in structuring bird abundance. To visualize patterns of variable importance among species, categorized scores were summarized using a dot matrix. All marginal effects for important wetland variables were provided as partial dependence plots (Appendix S1, Figures S13–S35). We assumed all eBird data used in our analysis to be an accurate depiction of large‐scale waterbird annual cycle distribution and relative abundance (Feng and Che‐Castaldo 2021; Fink et al. 2020; Horns et al. 2018; Stuber et al. 2022; Walker and Taylor 2017).

### 2.6 Wetland Trends

Long‐term surface water trends in the western U.S. were analyzed using ecological factors (identified from randomForestSRC analyses) that influenced waterbird distribution and abundance. This approach applied wetland surface water as a surrogate for functional wetland change to quantify shifts in hydrologic processes that affect waterbird resource availability. The wetland trends identified in our results should be interpreted as functional changes (e.g., wetland drying) associated with shifts in the extent and timing of annual inundation. Wetland trends were calculated using methods and data from Donnelly et al. 2025, which estimated monthly wetland surface water area and hydrology for the study area annually from 1984 to 2023. The spatial resolution, hydroperiod classes, and functional groups from these data matched those used in waterbird RandomForestSRC analyses. Long‐term wetland trends were assessed by averaging monthly hydroperiod, wetland area, and functional wetland group measures (from Donnelly et al. (2025)), within annual cycles for each waterbird species (see Table 2). This process was repeated for each year from 1984 to 2023. Using a masking function, results were clipped to species' annual cycle distributions based on eBird abundance layers. This approach limited the summary of wetland trends to regions where species abundance was estimated to be > 0. Maps showing species' annual cycle distributions are provided as Figures S1–S6. We assumed eBird abundance data (2008–2022) to be representative of the general timing of waterbird annual cycles throughout the period of the wetland trends from 1984 to 2023. Although it is likely that a changing climate has influenced the timing of these events, large uncertainties remain about their effects on waterfowl (sensu Frei et al. 2024). Similarly, changes in shorebird breeding and migration timing caused by climate change have been mixed, with long‐term studies showing no significant differences in arrival dates at breeding grounds (sensu Ely et al. 2018; Smith et al. 2010).

Wetland trends were determined by grouping wetland area, hydroperiod classes, and functional groups (see Table 3) for each species and annual cycle into two equal 20 years periods: 1984–2003 (P1) and 2004–2023 (P2). Comparing differences between these periods reduces the impact of short‐term climate cycles (e.g., El Nino Southern Oscillation) that could have influenced the results (Dettinger et al. 1998). Changes indicate shifts in resources affecting waterbird abundance. Wetland change was summarized using the average annual differences in pixel area between P1 and P2. These results serve as indicators of trends in wetland resource availability. Data are presented as a dot matrix to visualize patterns of change among species and annual cycles.

Boxplots depicting the annual variance in P1 and P2 for wetland variables important for shaping waterbird abundance are included as Appendix S1, Figures S36–S41 to provide a more detailed explanation of our findings. Differences were evaluated using Wilcoxon rank‐order tests (Siegel 1957). A *p*‐value of < 0.1 was used to represent statistical significance of the change. Additionally, tables were created to summarize wetland change as mean percentages between P1 and P2 by waterbird species and annual cycle (Tables S1–S6).

### 2.7 Data Processing

All image processing and raster‐based modeling were conducted using Google Earth Engine, a cloud‐based geospatial processing platform (Gorelick et al. 2017). GIS operations were performed using QGIS (QGIS Development Team 2020). Plotting and statistical analyses were generated using the R environment (R Core Team 2019; RStudio Team 2019), including R‐packages Tidyverse (Wickham et al. 2019) and RandomForestSRC (Ishwaran and Kogalur 2019).

## 3 Results

### 3.1 Variable Importance

Wetland area (i.e., wetland surface water area) was a key ecological factor for all waterbird species, acting as a universal predictor of abundance (Figure 4, Figures S7–S12). Waterbirds showed clear preferences for wetlands along gradients of hydroperiod persistence, with semi‐permanent wetlands being significant predictors of species' abundance across 87% of their annual cycles. In contrast, more ephemeral seasonal and temporary wetlands were significant predictors in 56% and 1% of annual cycles, respectively. Important functional groups included littoral saline lakes and palustrine wetlands, which were associated with approximately half of waterbird annual cycles. Managed wetlands, estuary, and rice importance were limited but notable predictors for some species. Littoral wetlands, grass‐hay wetlands, and ponds had limited overall importance for waterbirds. Inundated wetland area, on average, made up only 0.3% of land cover during the period associated with eBird abundance data (2008–2022).

Partial dependence plots showed positive relationships between waterbird abundance and important wetland features (i.e., hydroperiod classes and functional groups; Appendix S1, Figures S13–S35). Relationships were generally linear but included nonlinear exponential or asymptotic trends (Figure 6). Two outliers included negative relationships between areas of low‐density flooded rice and nonbreeding black‐necked stilt abundance (Figure S19). Additionally, “wetland area” showed a slightly negative to flat correlation with breeding canvasbacks (Figure S21). This result may have been influenced by low bird densities (< 0.37 birds 2.5 km^2^) and breeding distributions that represented only 3% of the continental population (*see* Table 1).

### 3.2 Species‐Level Variable Importance

#### 3.2.1 Shorebirds

**FIGURE 5:**
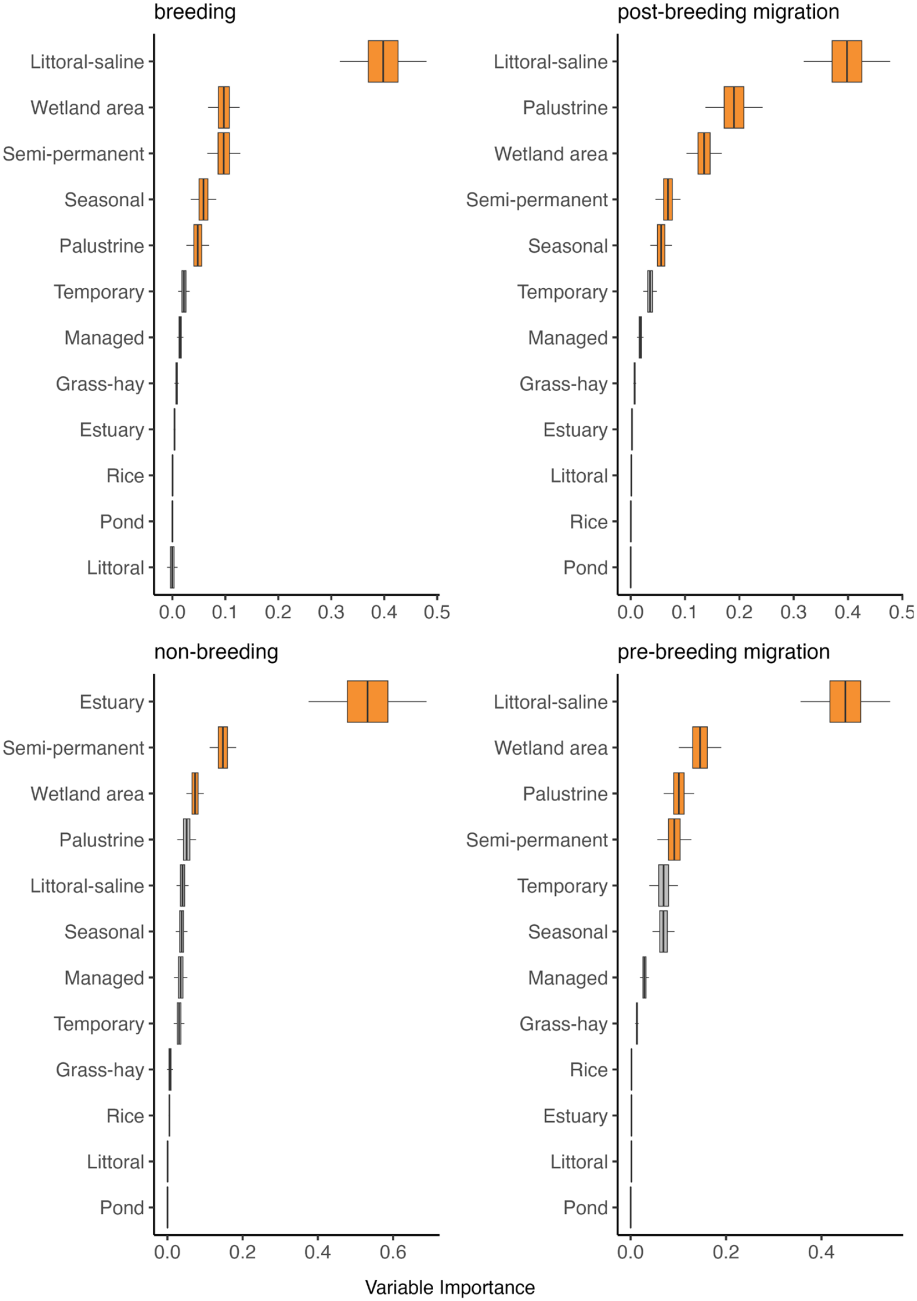
Variable importance scores for American avocets. High scores are partitioned to delineate wetland hydroperiod classes and functional groups that are important (red) in predicting broad‐scale abundance. Low‐scoring predictors (gray) are considered to have limited overall effects.

Littoral saline lake wetlands were a major predictor of shorebird abundance (Figure 4). Random forest VIMP scores ranked this functional group highest and clearly separated it from the other covariates for most species (Figure 5, Appendix S1, Figures S8 and S9). Shorebird preference for persistent and high‐density wetland landscapes was consistent with broader trends, as semi‐permanent wetlands and “wetland area” acted as universal predictors of their abundance. The importance of seasonal and palustrine wetlands was less prevalent but notable for some species' annual cycles. Outliers included estuarine wetlands as the top‐ranked predictor of nonbreeding American avocets (Figure 5) and managed wetlands and flooded winter rice as important predictors for nonbreeding black‐necked stilt abundance (Appendix S1, Figure S9).

#### 3.2.2 Waterfowl

**FIGURE 6:**
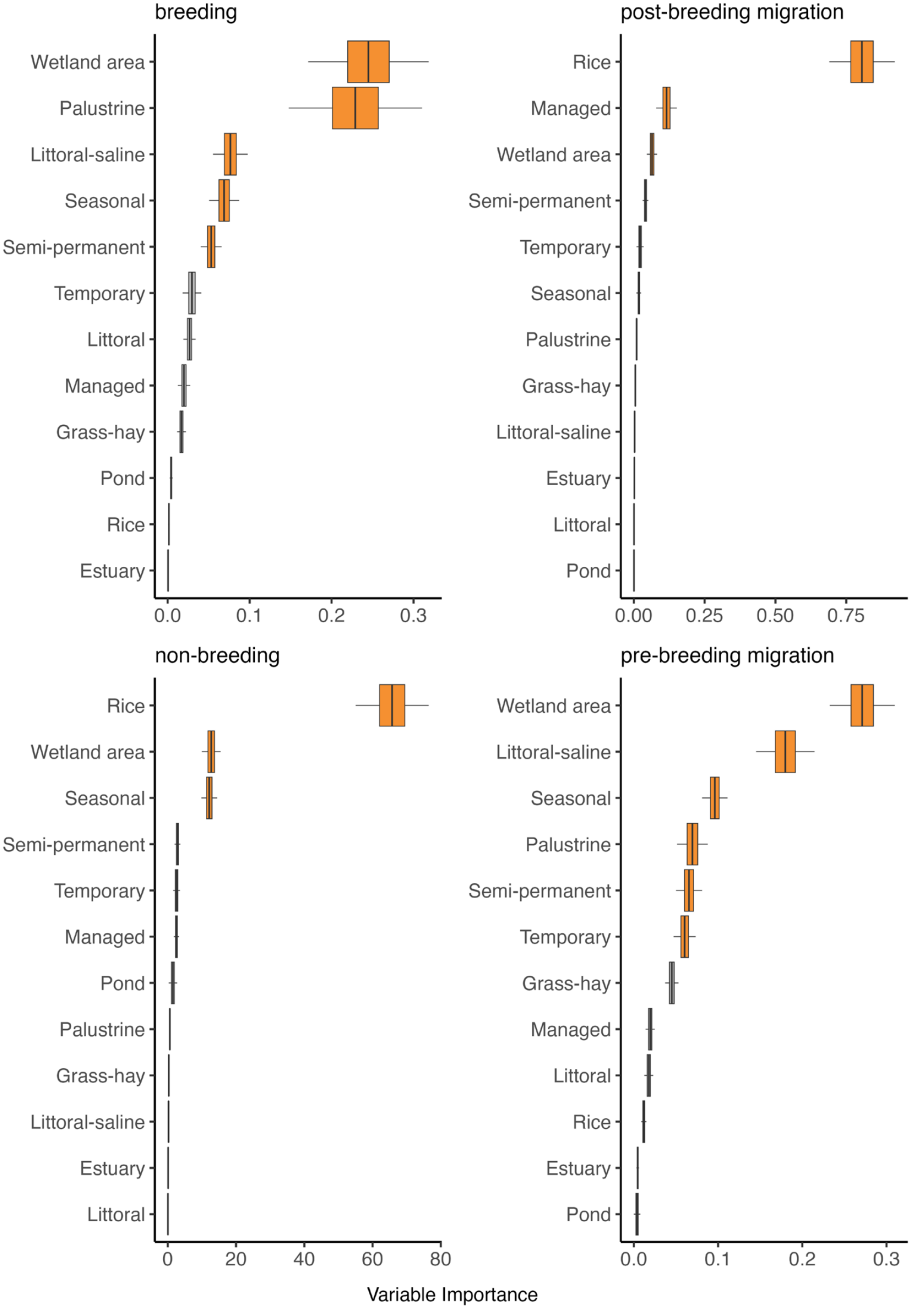
Variable importance scores for Northern Pintails. High scores are partitioned to delineate wetland hydroperiod classes and functional groups that are important (red) in predicting broad‐scale abundance. Low‐scoring predictors (gray) are considered to have limited overall effects.

“Wetland area” and winter‐flooded rice were the primary predictors of dabbling duck (i.e., cinnamon teal and northern pintails) abundance (Figure 6; Appendix S1, Figures S11 and S12). Other dabbling duck predictors included littoral saline‐lake and managed wetlands. Pre‐breeding migration dabbling ducks were the only waterbirds that exhibited abundance structured around temporary wetlands. Additional outliers included ponds and grass‐hay wetlands associated with cinnamon teal abundance during nonbreeding and pre‐breeding migration periods. Estuarine wetlands, “wetland area,” and semi‐permanent wetlands were the most important predictors of canvasback abundance (Appendix S1, Figure S10).

### 3.3 Wetland Trends

**FIGURE 7:**
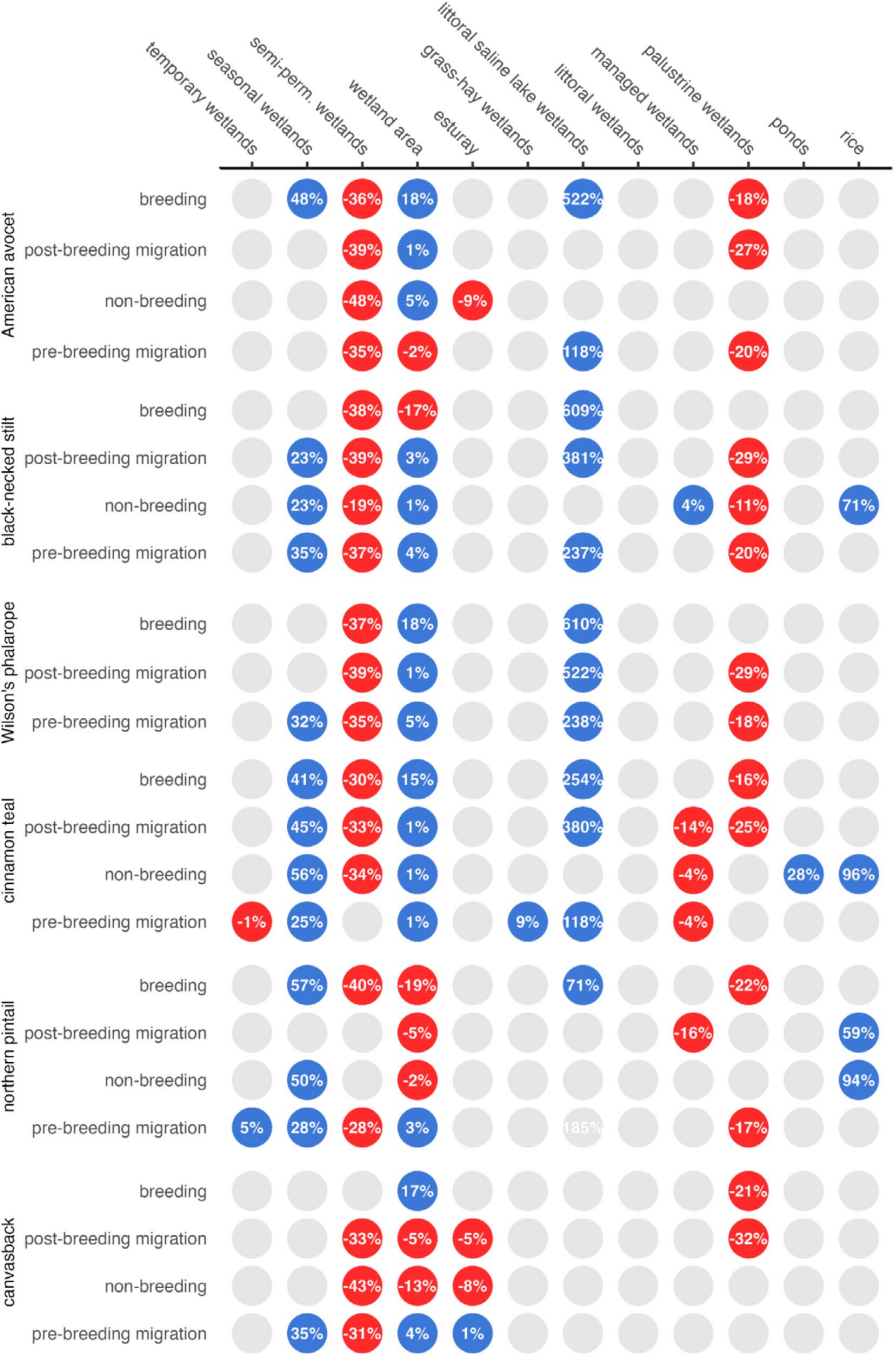
Change to wetland resources (x‐axis) identified as important to structuring waterbird abundance in the western U.S. Results are shown by species' annual cycle (y‐axis). Dots depict declines (red) and increases (blue) in mean annual wetland availability, represented by differences in inundated surface water extent between P1 (1984–2003) and P2 (2004–2023). Gray dots identify features with limited influence on bird abundance that were excluded from the analysis.

Approximately half of wetland predictors structuring waterbird abundance declined between P1 (1984–2003) and P2 (2004–2023; Figure 7). Declines of semi‐permanent and palustrine wetlands were universal, averaging 35% and 23% across species. Nearly all managed wetlands supporting black‐necked stilt, cinnamon teal, and northern pintail declined, with 4%–16% losses. Other declines included a majority of estuarine wetlands overlapping American avocet and canvasback distributions. Changes to “wetland area” were mixed, with declines to approximately a third of resources supporting American avocet, black‐necked stilt, canvasback, and northern pintails.

Littoral saline lake wetlands were the only functional group to experience exponential growth, expanding between 71% and 609% between P1 and P2 (Figure 7). Similarly, important temporary and seasonal wetland predictors exhibited nearly universal expansion, increasing from five to 57%. In agroecological systems, ponds and winter‐flooded rice, associated with black‐necked stilt, cinnamon teal, and northern pintail abundance, increased between 26% and 96%. Additionally, grass‐hay wetlands, supporting pre‐breeding migration cinnamon teal abundance, increased by 9%.

## 4 Discussion

Our analysis intersects migratory waterbird annual cycles with long‐term wetland trends to identify emerging risks to habitat networks in the western U.S. Resource scarcity was the driver of waterbird distributions as inundated wetlands within the study area accounted for only 0.3% of land cover. Waterbirds offset limited resources by prioritizing the use of predictable high‐density wetland landscapes, as evidenced by “wetland area” acting as a universal predictor of bird abundance. Ubiquitous declines in nearly half of predictive wetland systems were indicators of landscape drying that portend an irreversible and permanent shift in ecosystem water balance across portions of North American waterbird flyways. Our improved understanding of wetland and waterbird interactions across annual cycles elevates the urgency for developing adaptive conservation strategies to sustain continental habitat networks that are rapidly evolving under the stress of ongoing surface water declines.

### 4.1 Wetland Importance

Wetland scarcity drove density dependence in waterbirds as “wetland area” was a constant predictor of bird abundance. Spatial inventories in the western U.S. have concluded that wetlands account for 1%–3% of landscapes (Tiner 2003); however, wetland conditions may further restrict habitat availability as our results show that inundated wetlands accounted for only 0.3% of land cover. Moreover, findings from Donnelly et al. (2025) showed that 60% of inundated wetlands occurred in regions comprising only 17% of the western U.S. These relationships suggest that waterbirds concentrate in a limited number of high‐density wetland landscapes to overcome otherwise scarce resource abundance (i.e., inundated wetland habitats). Similar patterns of density dependence have been demonstrated at local and regional scales, showing wetland area as a key predictor of breeding and migrating waterbird distributions (Albanese and Davis 2015; Elliott et al. 2019; Elmore et al. 2024; Webb et al. 2010).

More persistent semi‐permanent systems functioned as a higher‐valued waterbird resource, likely due to reduced uncertainty around habitat availability in otherwise dynamic and wetland‐limited landscapes. Recent findings link waterbird preference for semi‐permanent wetlands to differences in resource predictability and food densities among wetland hydrologies (i.e., semi‐permanent, seasonal, and temporary). While waterbird energetic studies are limited in the western U.S., higher macroinvertebrate densities, an important food resource for shorebirds and waterfowl, have been attributed to semi‐permanent wetlands (Setash, Behney, Gammonley, and Koons 2024). Higher macroinvertebrate densities were associated with increased breeding waterfowl abundance and persistent surface water conditions, which supported more complex and productive aquatic communities (sensu Schad et al. 2020). Other studies have shown seasonal and temporary wetlands (in the western U.S.) to produce higher seed abundance and similar macroinvertebrate densities (compared to semi‐permanent wetlands). However, these systems were less predictable due to seasonal ephemerality, which limited their annual availability (Gammonley and Laubhan 2002).

**FIGURE 8:**
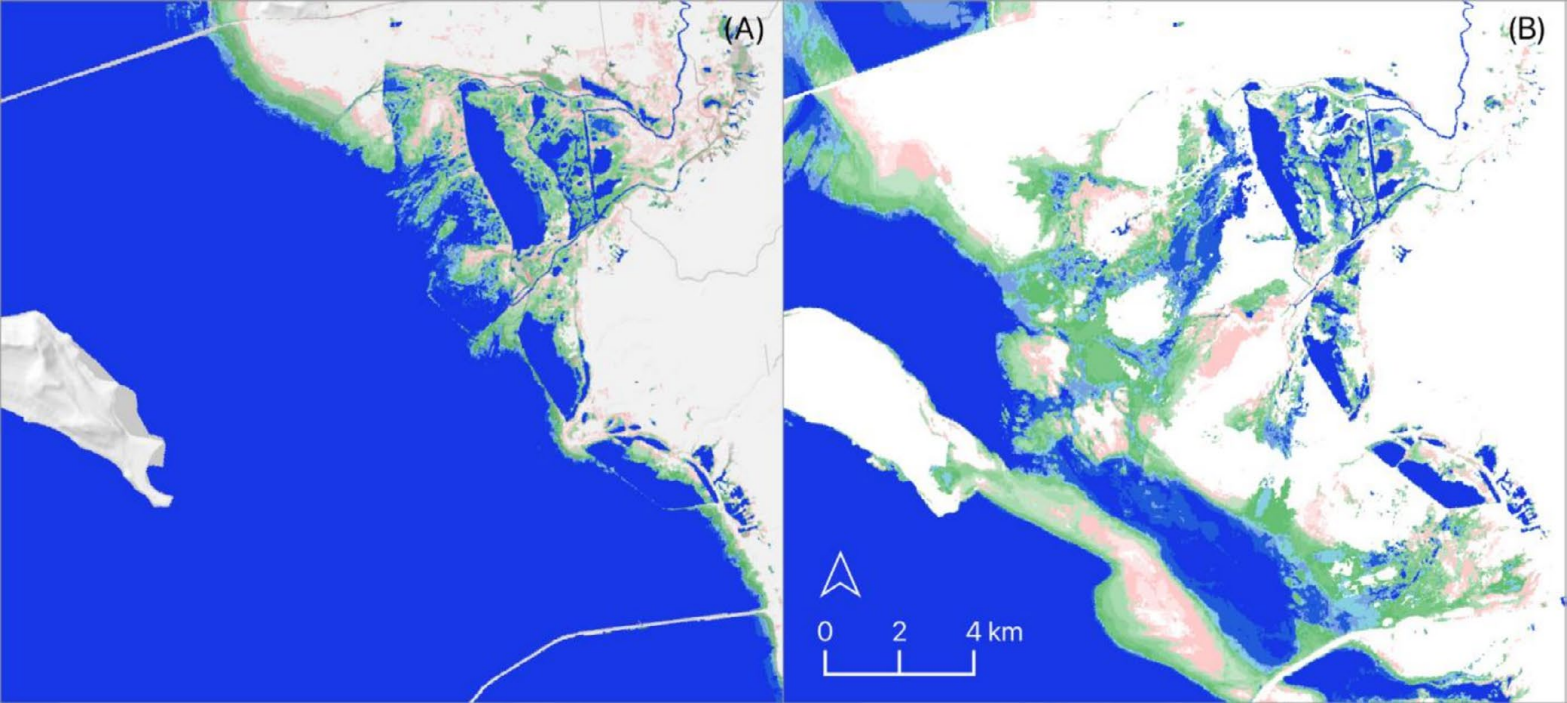
Ogden Bay, Great Salt Lake showing littoral seasonal (green) and temporary (green) wetland extent for May 2001 (A) and 2016 (B). Contracting lake extent (blue—lower left) increases littoral wetlands along the fringe and below freshwater inputs as outflows from managed wetlands in the upper right.

Expansion of littoral wetlands identified in our results was likely due to saline lake desiccation. Declining lake elevations may offset broader wetland decline and benefit waterbirds through near‐term increases in habitat availability (Donnelly et al. 2022). The exponential growth of these systems was visible in wetland mapping layers that showed the spread of seasonal and temporary wetlands onto exposed lake beds below points of freshwater discharge and along the periphery of receding lake water bodies (Figure 8). Resulting processes supported highly heterogeneous wetland conditions that concentrated productive saline and freshwater systems in close proximity, allowing waterbirds to leverage a greater diversity of habitat resources. Despite seasonal and temporary wetland ephemerality, their availability remained persistent due to changing water levels that drove seasonal creation and redistribution of resources along lake peripheries. Under this scenario, saline lake ecosystems offset uncertainty in resource availability, resulting in diverse, highly predictable wetland environments that drove waterbird abundance.

Natural (i.e., unmanaged) palustrine and estuarine wetlands were significant drivers of waterbird abundance. In contrast, the importance of managed wetland systems was constrained by a limited geographic extent compared to more abundant and dispersed natural systems. For example, managed wetland reliance observed in black‐necked stilts, cinnamon teal, and northern pintails was due to localized bird concentrations in the Central Valley of California, where densities of managed systems were high (Donnelly et al. 2022). Regional studies have shown that naturally occurring palustrine wetlands may also act as a limiting factor in landscape suitability when structuring waterbird use of anthropogenic habitats. For example, King et al. (2010) found waterbirds' use of cultivated rice was influenced by the surrounding distribution of natural wetlands, which filled crucial gaps in habitat diversity, allowing birds to leverage agricultural resources more effectively. Similarly, Sebastián‐González et al. (2010) demonstrated that the availability of adjacent natural wetlands enhanced waterbird use of constructed ponds.

**FIGURE 9:**
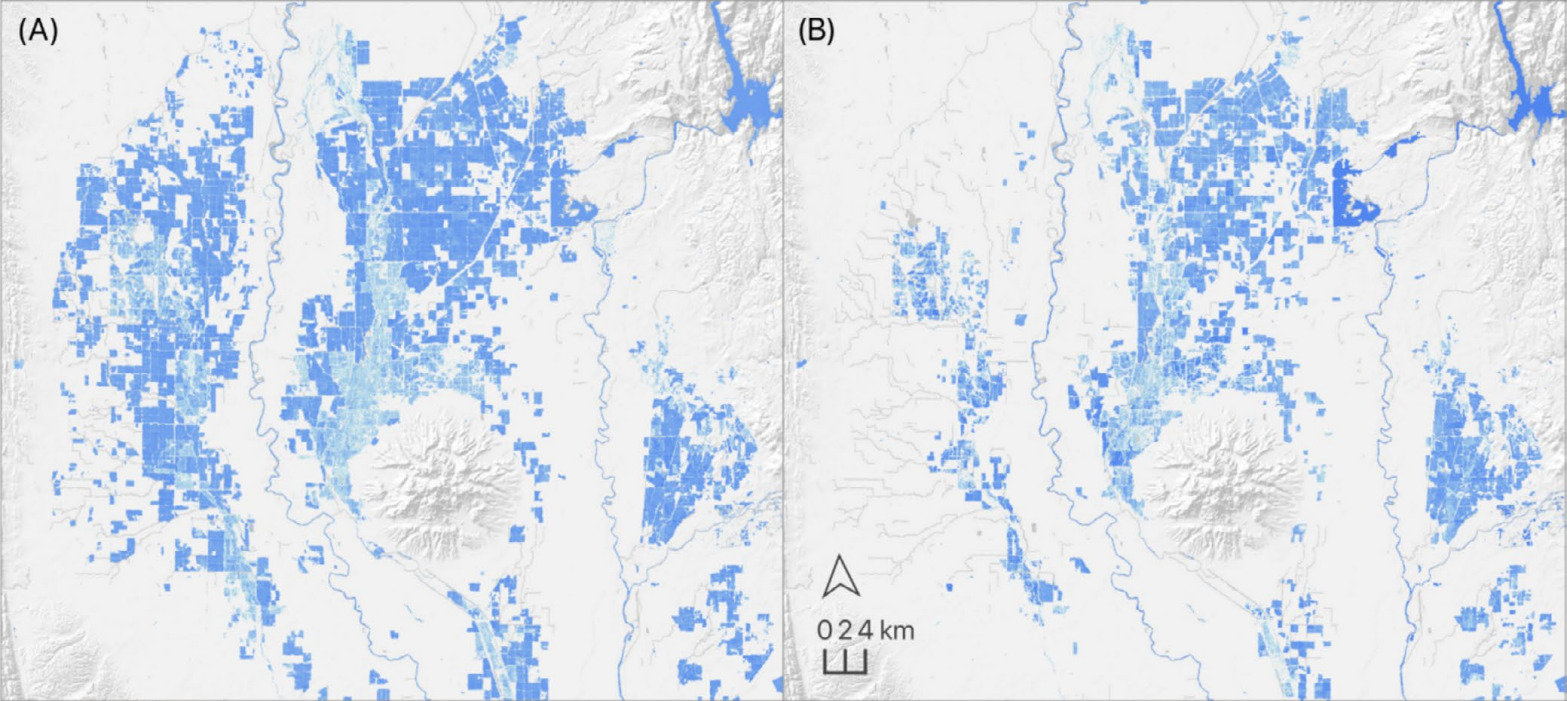
Flooded winter rice (blue) in California's Central Valley showing typical conditions in December 2023 (A) and severe drought conditions during December 2022 (B). Flooding was reduced by ~60,000 ha on the west half of the valley, confining inundation to managed wetlands. Wetland surface water delineations extracted from the WET dataset (Donnelly 2021).

The limited influence of rice cultivation as a predictor of waterbird abundance was attributed to its narrow geographic extent and temporal availability, which largely constrained resource availability to nonbreeding and pre‐breeding migration of dabbling ducks (i.e., cinnamon teal and northern pintail) in the Central Valley of California. In this geography, rice supports one of the largest nonbreeding concentrations of waterfowl in North America, providing approximately 56% of food energy to birds through waste grain and post‐harvest winter flooding of fields (United States Fish and Wildlife Service [USFWS] 2020). The expansion of winter‐flooded rice overlapping dabbling duck abundance was attributed to changing environmental regulations associated with the Clean Air Act https://paperpile.com/c/vEo4d4/AAarR/?noauthor=1 (Clean Air Act Amendments 1990), which eliminated burning in favor of winter flooding to remove (i.e., decompose) rice stubble in harvested fields beginning in the mid‐1990s (Miller et al. 2010). While currently stable, rice‐supported waterfowl habitat is threatened by long‐term drought and declining snowpack, which has reduced irrigation capacity, leading to intermittent declines in winter‐flooded rice (Donnelly et al. 2022). In 2022, for example, diminished water storage in upstream reservoirs constrained winter rice flooding to approximately half its typical extent, substantially reducing food energy availability for nonbreeding waterfowl (Figure 9). Such events highlight a potential shortcoming in assessing long‐term wetland trends, suggesting that evaluating the impacts of increasingly frequent short‐term severe drought events on waterbirds is warranted.

Regional tracking and resource selection studies supported our model outcomes, identifying ponds as an important predictor of nonbreeding cinnamon teal abundance. Nonbreeding bird distributions occurring in southwestern portions of the study area occupied landscapes where ponds accounted for 51%–88% of available wetland resources (Donnelly et al. 2025). Recent findings from Mackell et al. (2021) using GPS‐marked cinnamon teal showed that ponds are essential in shaping bird movements during nonbreeding periods, particularly in arid landscapes with limited natural wetlands. Pond abundance associated with cinnamon teal distributions has increased significantly over the past two decades, partly offsetting wetland losses in some regions (Donnelly et al. 2025). Expansion has been associated with cattle production and increased heat‐stress days on livestock in the western U.S., requiring ranchers to develop improved water access to maintain operations (Reeves et al. 2017).

The minimal influence of grass‐hay wetlands on broad‐scale waterbird abundance was attributed to the misalignment between land use practices and species' life history needs. Findings from Setash, Behney, Gammonley, Pejchar, et al. (2024) found that breeding dabbling ducks actively avoided grass‐hay pastures as nesting habitats due to lower plant density and diversity compared to uncropped areas. Haying and grazing may also reduce residual seed abundance, a food resource for some waterfowl species (Fredrickson and Reid 1988). While flood‐irrigated grass‐hay agriculture is known to support a majority of greater sandhill cranes breeding in the western U.S. (Donnelly, Collins, et al. 2024), its predominantly temporary wetland hydrologies harbor fewer macroinvertebrates (food energy) than more perineal (e.g., semi‐permanent) systems (Setash, Behney, Gammonley, and Koons 2024) which were key predictors of waterbird species evaluated in this study. Further, the predominance of peak grass‐hay wetlands abundance in June (Donnelly, Jensco, et al. 2024) fell outside periods where regional studies have identified benefits to waterfowl during post‐breeding migration. For example, anomalous March and April grass‐hay flood irrigation in portions of the SONEC region has been shown to provide important stopover habitat supporting large concentrations of dabbling ducks (Fleskes and Yee 2007).

### 4.2 Flyway Function

**FIGURE 10:**
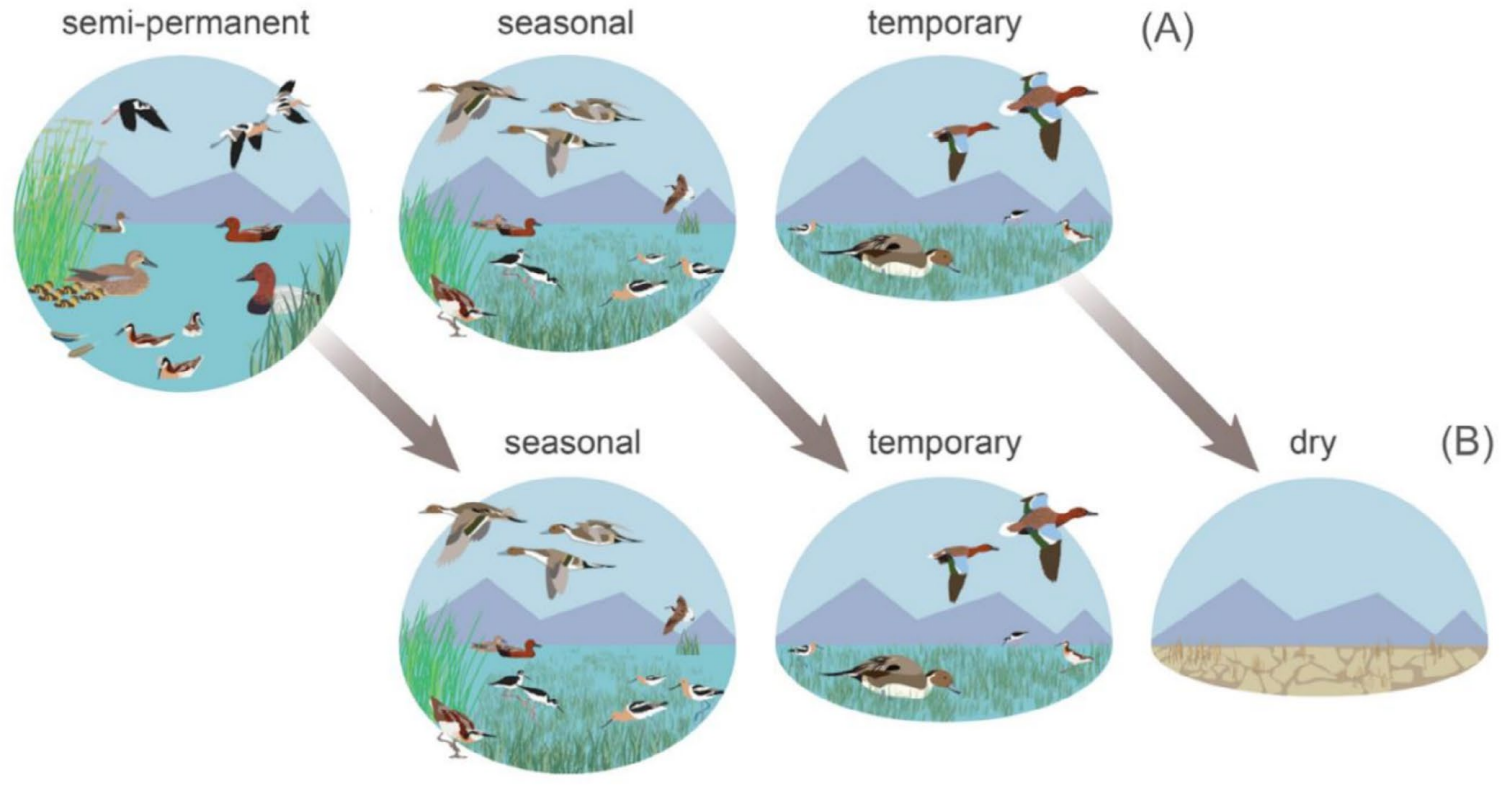
Functional wetland declines showed disproportionate impacts on waterbird species reliant on semi‐permanent wetlands during their annual cycle. Semi‐permanent losses (A) resulted from shortened hydroperiods caused by excessive drying that forced wetland transition to seasonal and temporary hydrologies—a process that offset concurrent seasonal and temporary wetland declines (B).

Semi‐permanent declines were early indicators of large‐scale functional loss, signaling a transition of waterbird habitat networks along a continuum of persistent to more ephemeral states. Losses resulted from shortened hydroperiods caused by excessive drying, which forced semi‐permanent conversion to seasonal and temporary hydrologies—a process apparent in our analysis that partially offsets concurrent seasonal and temporary wetland declines (Figure 10). This scenario can result in disproportionate impacts on waterbird species with life histories reliant on semi‐permanent wetland systems. For example, in 2020, ~60,000 waterfowl were lost on a single wildlife refuge in SONEC due to a disease outbreak attributed to declining semi‐permanent wetland abundance, which increasingly concentrated birds on these limited habitats when used as molting refugia during late summer (Sabalow 2020). Our results showed semi‐permanent wetland losses of 30%–38% in western U.S. distributions of American avocet, black‐necked stilt, cinnamon teal, and Wilson's phalarope that overlapped 58%, 49%, 84%, and 90% of their continental breeding populations. While Wilson's phalarope abundance has declined by 75% since the mid‐1990s (Smith et al. 2023), American avocet, black‐necked stilt, and cinnamon teal populations are considered stable (Ackerman et al. 2020; Gammonley 2020; Robinson et al. 2020). However, these species are poorly monitored, and it is unclear what effect recent semi‐permanent losses have had on populations.

High waterbird reliance on saline lakes, as evidenced by our results, furthers concerns over the sustainability of flyway habitat networks. While the expansion of littoral wetlands due to saline lake desiccation partly offset near‐term habitat declines, they were additional indicators of landscape drying (Donnelly et al. 2020). Since 2003, surface water in the western U.S. closed basin saline lake systems has declined by 27% (Donnelly et al. 2020). Potential waterbird impacts from lake drying are well documented, identifying lower water volumes and higher salinity as increasing risks for trophic collapse in aquatic food webs sustaining waterbirds (Rubega and Robinson 1997; Senner et al. 2018). Increased salinity has also been linked to reduced chick survival in breeding shorebirds (e.g., American avocet) due to the limited capacity of young birds to metabolize salts (Haig et al. 2019). Transition of some declining freshwater lakes to saline states may open habitat niches in the western U.S. to offset losses in others; however, these lakes may also be vulnerable to collapse if their freshwater inflows continue to diminish (sensu Thomas 1995).

**FIGURE 11:**
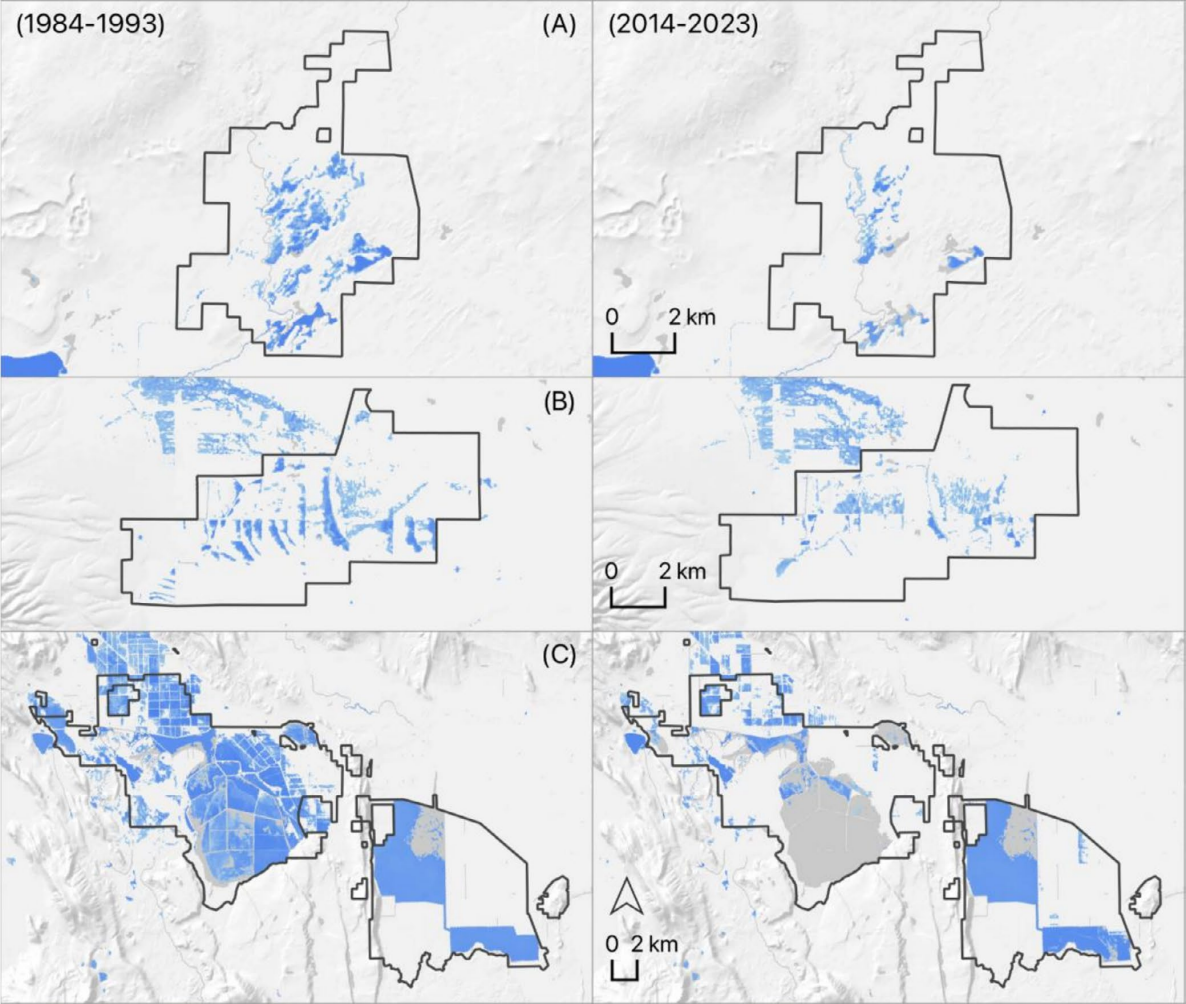
Examples illustrating the declining managed wetland capacity on Camas (A), Monte Vista (B), and Lower Klamath/Tule Lake National Wildlife Refuges (C) as an indirect effect of drought and changing agricultural water use practices. Change shown by comparing managed wetland inundation (blue) between 10‐year averages (1984–1993 and 2014–2023) for May (Camas and Monte Vista) or March (Lower Klamath/Tule Lake). Refuge boundaries are defined by black polygons—areas of blue outside polygons designate adjacent wetland resources. Wetland surface water delineations extracted from the WET dataset (Donnelly 2021).

Declines in managed wetlands coincident with black‐necked stilt, cinnamon teal, and northern pintail post‐breeding migration were indicators of broader water use and policy changes that have indirectly impacted waterbird habitats on public wildlife refuges (Figure 11). For example, the expansion of irrigated agriculture and associated groundwater pumping around Camas National Wildlife Refuge in eastern Idaho, USA, has lowered water table elevations by five meters, substantially reducing wetland surface water and waterbird habitat values the refuge was initially established to protect (Rattray 2017). Similarly, large public refuges in the San Luis Valley (Colorado, USA), created to sustain migratory waterbirds, have lost 45% of their managed wetland capacity, declining from 7200 to 3300 ha. over the past two decades (Wetland Dynamics 2019). Declines were attributed to long‐term drought and over‐allocation of groundwater for agricultural irrigation, which have triggered moratoriums on usage, eliminating the application of artesian well water historically employed by refuge managers to augment surface water demands for wetland management. Other impacts included the Lower Klamath and Tule Lake National Wildlife Refuges (California, USA), whose managed wetlands once supported over 3 million northern pintails during post‐breeding migration (Gilmer et al. 2004) but recently are dry due to drought and the reprioritization of declining water stores to support endangered fish and irrigated agriculture (National Marine Fisheries Service [NMFS] 2019).

Minor declines in estuarine wetlands associated with some nonbreeding American avocet and canvasback were likely a result of restoration. As illustrated by eBird abundance maps (Figures S1 and S3), waterbird distributions showed focused use in the California Bay‐Delta Region, encompassing the largest estuarine system within the study area. Large‐scale efforts to restore tidal connectivity have significantly affected local wetland conditions (sensu Kerr et al. 2016). For example, single projects converting evaporation salt ponds to estuarine marshes affected more than 6000 ha (Callaway et al. 2011). Outcomes have increased sediment accretion and revegetation of shallow bays, reducing open water in estuarine wetlands identified in our surface water models. Estuarine restoration, while reducing wetland surface water through revegetation, has been linked to increased waterfowl and shorebird diversity and multi‐species benefits in the California Bay‐Delta Region (Casazza et al. 2021).

## 5 Conclusions

The overlap of landscape drying with waterbird distributions signals the potential emergence of new and powerful ecological bottlenecks in western North American habitat networks. Patterns of wetland loss were ubiquitous, indicating impacts across waterbird life histories through reduced predictability and diminished abundance of habitat resources. While these effects were derived using a selection of representative species, their impacts were likely emblematic of associated waterbird guilds reliant on concurrent wetland networks. North American droughts impacting wetland resources are projected to intensify in the coming decades (Bradford et al. 2020), increasing the urgency to improve our understanding of potential flyway scale effects. Xu et al. (2024) predict an average wetland loss of 10% in the U.S. by 2100, with most systems transitioning towards more pronounced ephemeral states due to reduced snowpack and higher summer evaporation rates. The decline of semi‐permanent wetlands structuring waterbird abundance provides an early indicator of changing flyway conditions. Recent findings from Donnelly et al. (2025) show overall wetland losses in important waterbird landscapes of Great Salt Lake and SONEC, have already exceeded long‐term predictions with declines of 13% and 21% over the past two decades.

Existing and upcoming changes to waterbird flyways will seriously challenge current natural resource management and conservation efforts, requiring forward‐looking approaches to enable adaptation. The primary approach to waterbird conservation has been the establishment of permanent protections for habitats rather than the protection of underlying ecological functions that generate them (Holling and Meffe 1996). As the rapid evolution of wetland ecosystems and land use change alter surface water hydrology, established land protection networks risk becoming increasingly misaligned with species conservation goals. Addressing change will require reevaluating common perspectives and values built on assumptions that ecological baselines remain stationary (Adger et al. 2013). For example, climate resilience in wetland ecosystems identified in regions of Mexico (Donnelly et al. 2020) provides opportunities to increase targeted conservation in areas more likely to sustain nonbreeding (i.e., wintering) waterbirds in the future. This would require a substantial shift in established funding strategies and international collaboration to achieve.

Enabling adaptive flyway conservation will require novel investments in our ability to monitor continental habitat change in concert with dynamic waterbird interactions. Here we demonstrate efficient solutions at relatively low cost by combining publicly available satellite imagery with no‐cost (for noncommercial use) cloud‐based computing platforms (sensu Gorelick et al. 2017) and eBird's global citizen science data (Sullivan et al. 2014). Integration of this approach into existing North American waterfowl and shorebird conservation initiatives can provide a catalyst for innovative solutions to inform the sustainability of continental waterbird populations and wetland networks into the current century. Assimilation would enable strategies beyond a preservationist framework (e.g., static land protection) by including those that allow for the conservation of ecological processes sustaining flyway function (sensu Moore and Schindler 2022). We encourage collaborative use of our data among stakeholders to inform wetland and waterbird preservation for an increasingly dynamic and unpredictable future.

## Author Contributions

**J. Patrick Donnelly:** conceptualization (lead), data curation (lead), formal analysis (lead), investigation (lead), methodology (lead), resources (lead), validation (lead), writing – original draft (lead). **Johnnie N. Moore:** writing – review and editing (supporting). **John S. Kimball:** writing – review and editing (supporting). **Shea Coons:** visualization (supporting). **Daniel P. Collins:** data curation (supporting), funding acquisition (supporting), resources (supporting). **Mark J. Petri:** writing – review and editing (supporting). **David E. Naugle:** writing – review and editing (supporting).

## Conflicts of Interest

The authors declare no conflicts of interest.

## Supporting information


**Appendix S1:** ece373061‐sup‐0001‐AppendixS1.docx.

**Appendix S2:** ece373061‐sup‐0002‐AppendixS2.docx.

## Data Availability

Shorebird and waterfowl relative abundance data used in this analysis may be accessed through eBird (https://ebird.org/). Wetland hydroperiod and functional classification data used to train Random Forest Models are available as a table (https://drive.google.com/file/d/193G‐oAwzwTQLeguANafegN46wsR3Z4K6/view?usp=sharing).
