# Correction to “eBird Data Highlight Shifts in Wetland Resources Structuring Waterfowl and Shorebird Abundance”

**DOI:** 10.1002/ece3.73219

**Published:** 2026-03-09

Donnelly, J. P., J. N. Moore, J. S. Kimball, et al. 2026. “eBird Data Highlight Shifts in Wetland Resources Structuring Waterfowl and Shorebird Abundance.” *Ecology and Evolution* 16, no. 2: e73061. https://doi.org/10.1002/ece3.73061.

In the original article, the author name Mark J. Petri is incorrect. This should be Mark J. Petrie.

The online version of this article has been corrected accordingly.

We apologize for this error.

