# Supplementary material for: eBird Data Highlight Shifts in Wetland Resources Structuring Waterfowl and Shorebird Abundance
Source: Ecol Evol. 2026 Feb 17;16(2):e73061. doi: 10.1002/ece3.73061 (PMC12913221; doi:10.1002/ece3.73061)
Supplement: Supplementary file 1 — Appendix S1: ece373061‐sup‐0001‐AppendixS1.docx. [file ECE3-16-e73061-s001.docx]

Appendix S1. Supplementary figures and tables

1.1 eBird relative abundance and distribution maps

Figures S1-S6 show eBird mean relative abundance for American avocet (*Recurvirostra americana*, hereafter “avocets”), black-necked stilts (*Himantopus mexicanus*), canvasbacks (*Aythya valisineria*), cinnamon teal (*Anas cyanoptera*), northern pintail (*Anas acuta*), and Wilson’s phalarope (*Phalaropus tricolor*). Data are clipped to the study area and partitioned by seasonal life history (i.e., breeding, post-breeding migration, non-breeding, and pre-breeding migration).


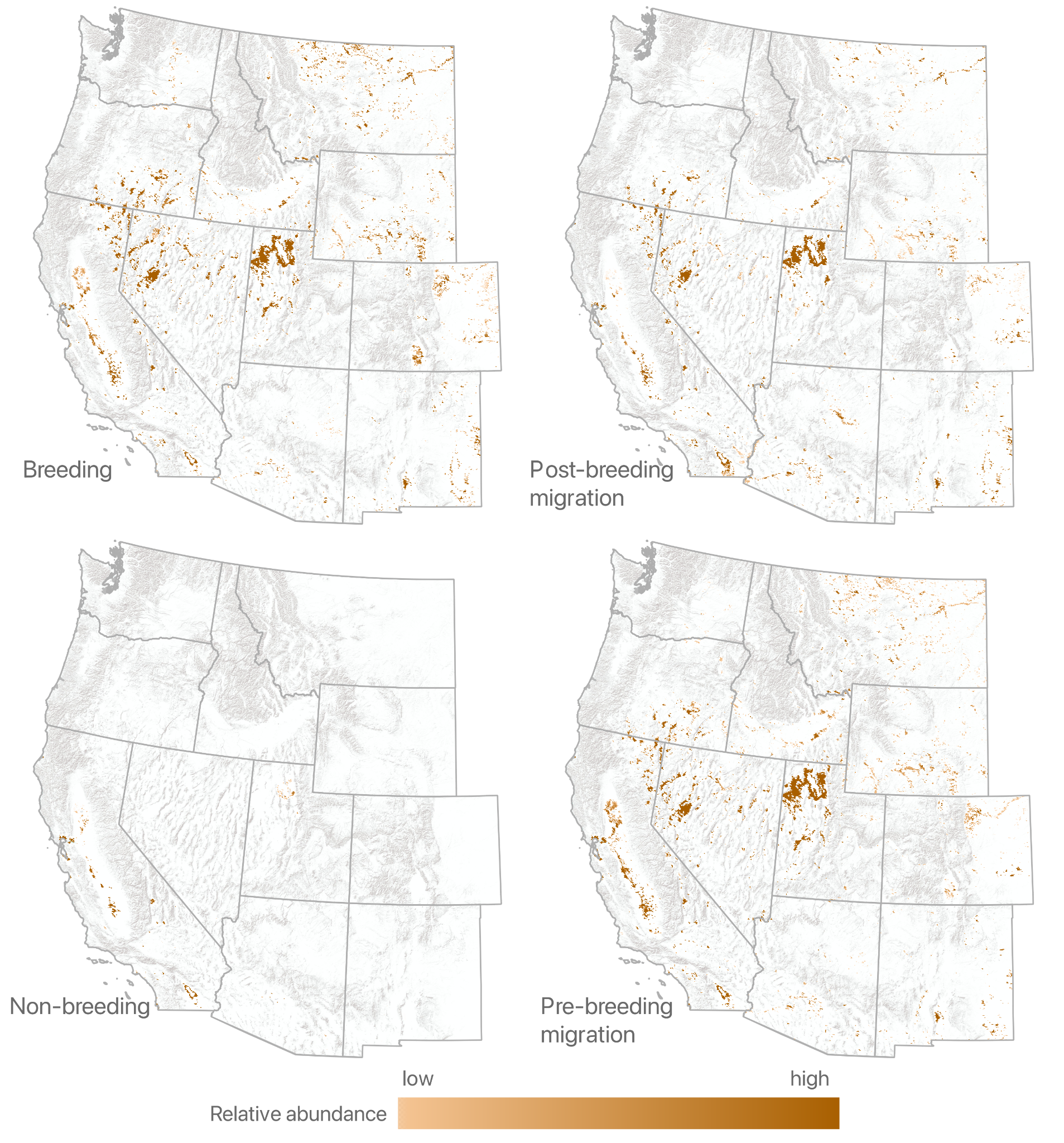


Figure S1. American avocet relative abundance—explanation of results as referenced previously.


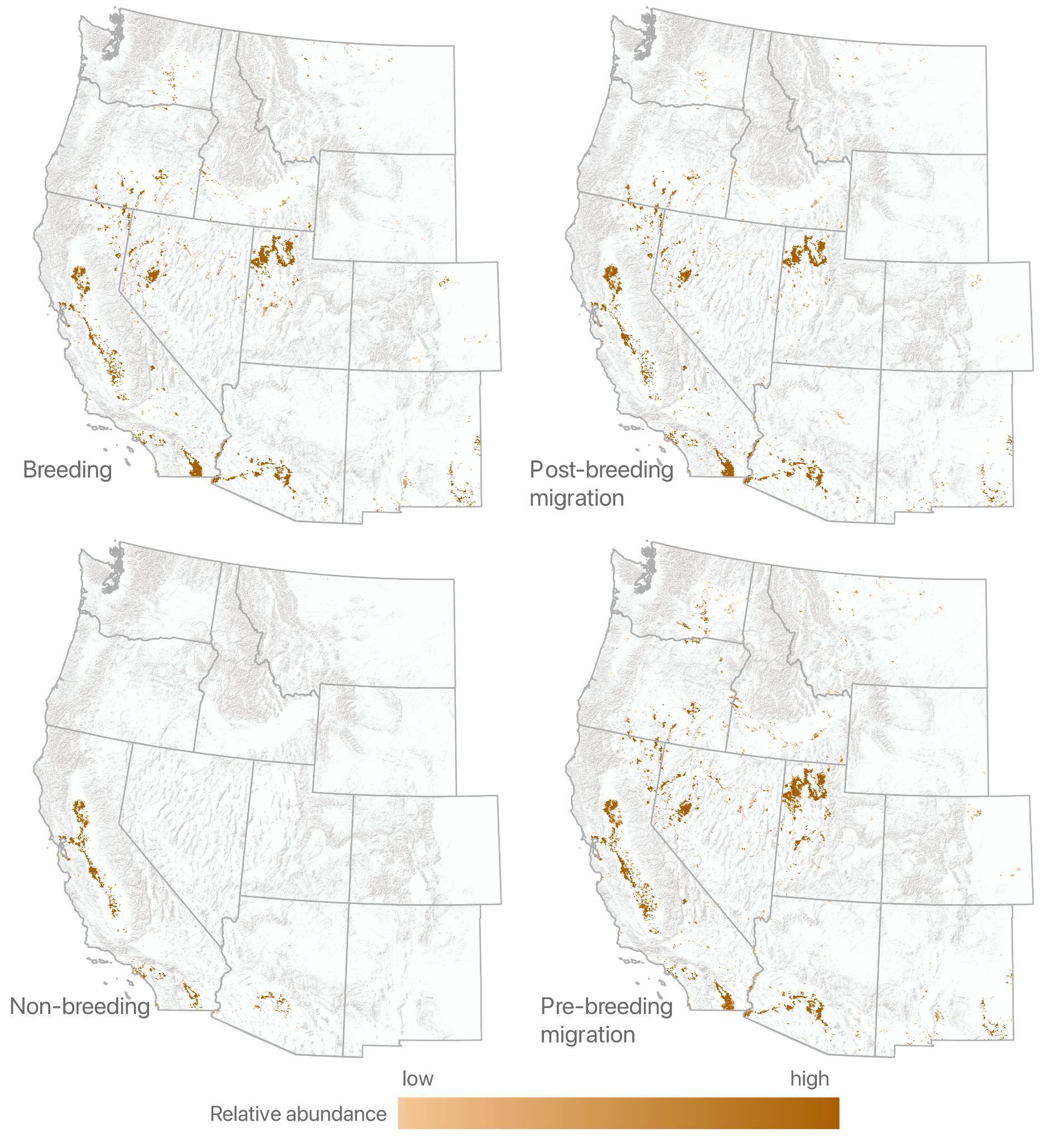


Figure S2. Black-necked stilt—explanation of results as referenced previously.


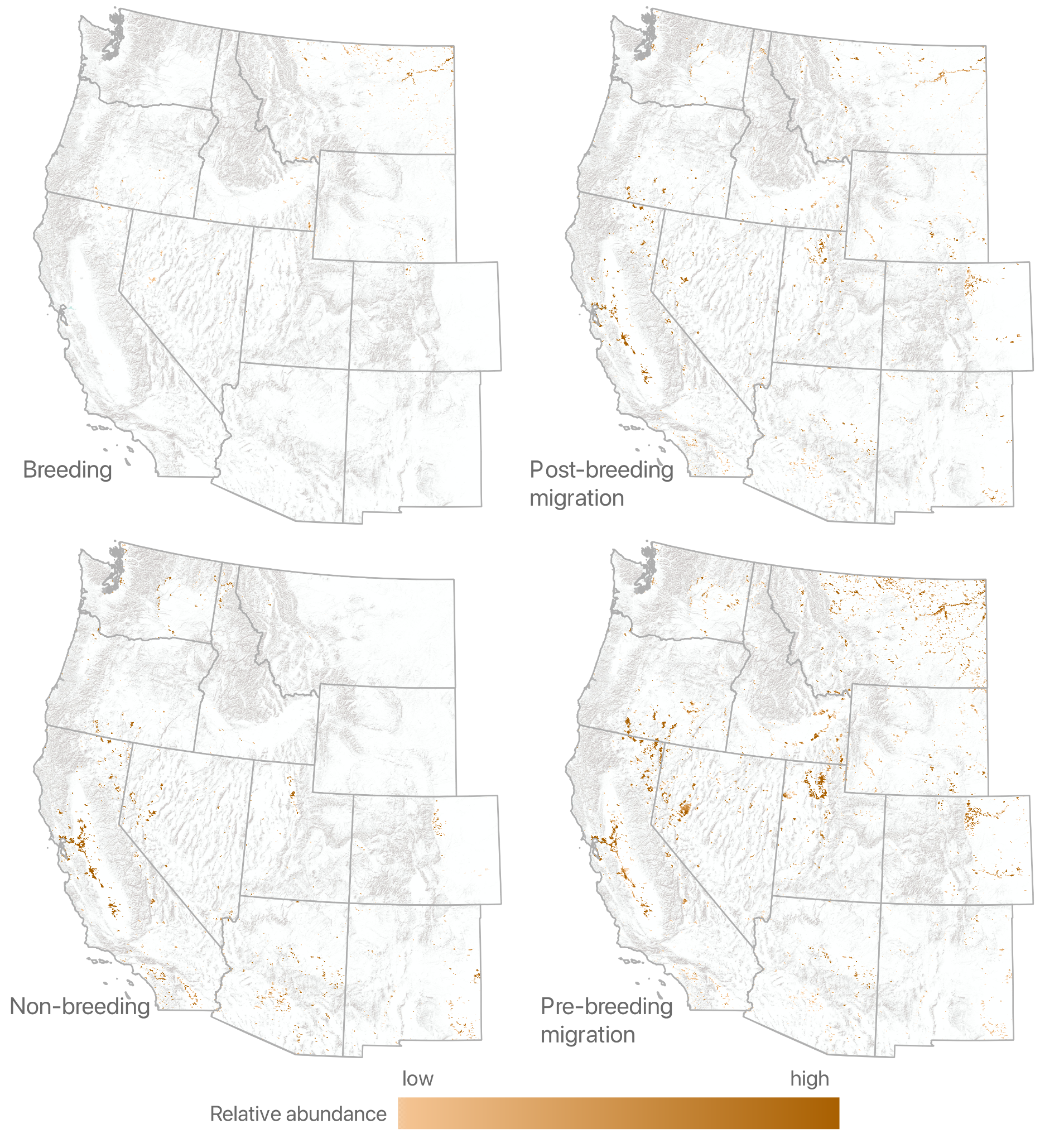


Figure S3. Canvasback—explanation of results as referenced previously.


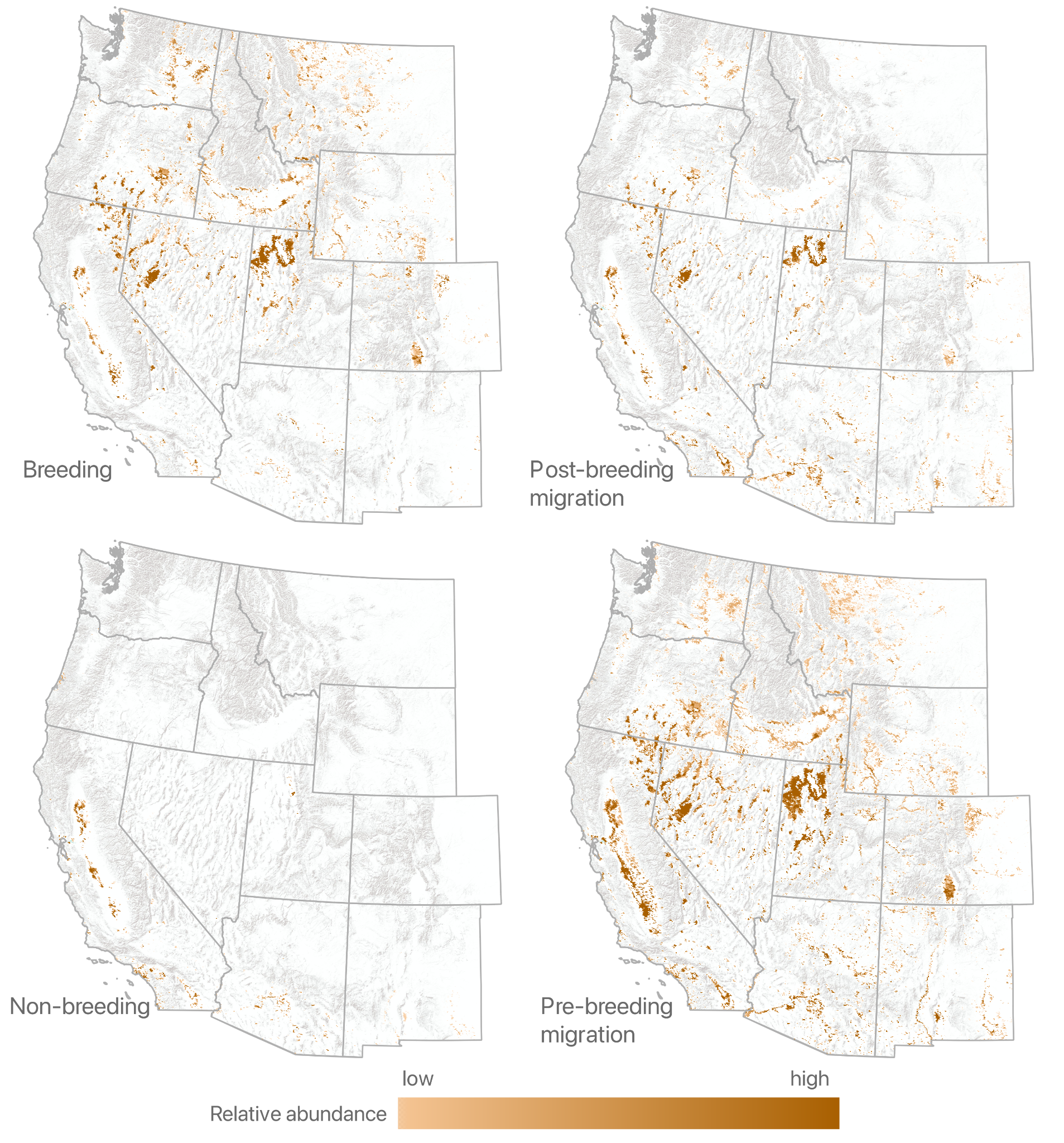


Figure S4. Cinnamon teal—explanation of results as referenced previously.


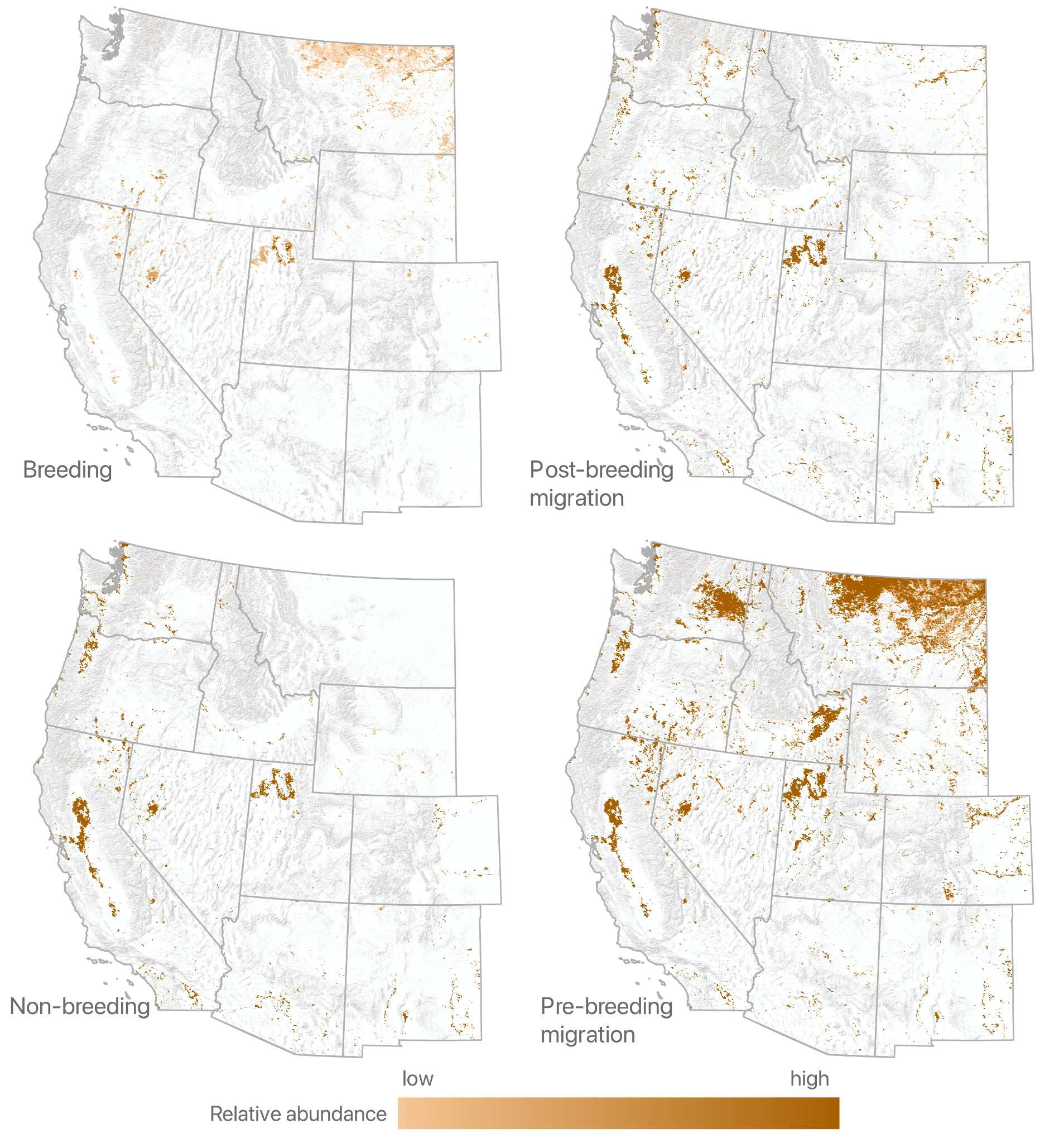


Figure S5. Northern pintail—explanation of results as referenced previously.


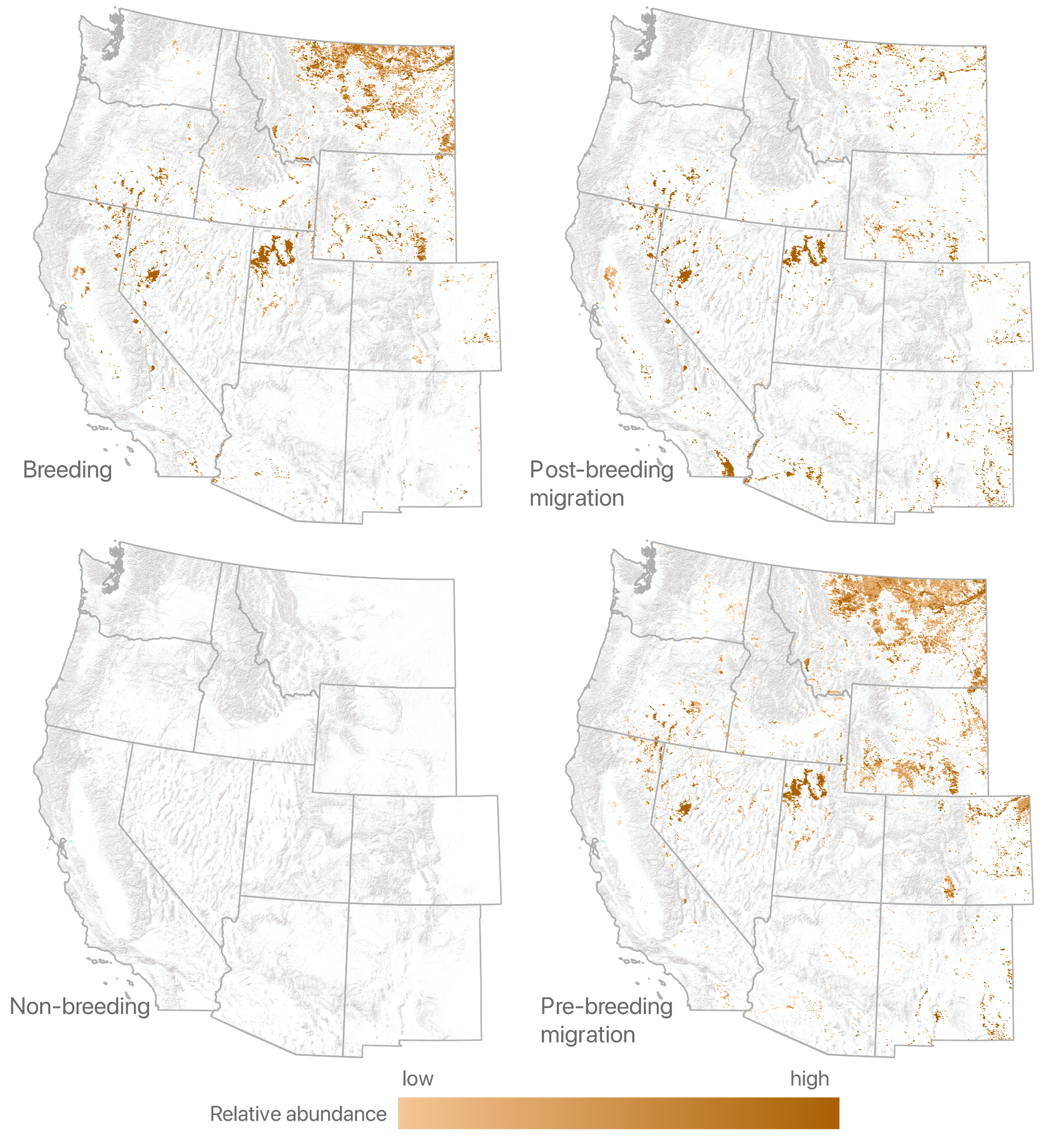


Figure S6. Wilson’s phalarope—explanation of results as referenced previously.

1.2 RandomForestSRC wetland variable importance

Figures S7-S12 show RandomForestSRC wetland variable importance (VIMP) scores by species and seasonal life-history. VIMP scores important to structuring waterbird distributions are shown in green. Low-scoring predictors (gray) are considered to have a limited effect on bird abundance.


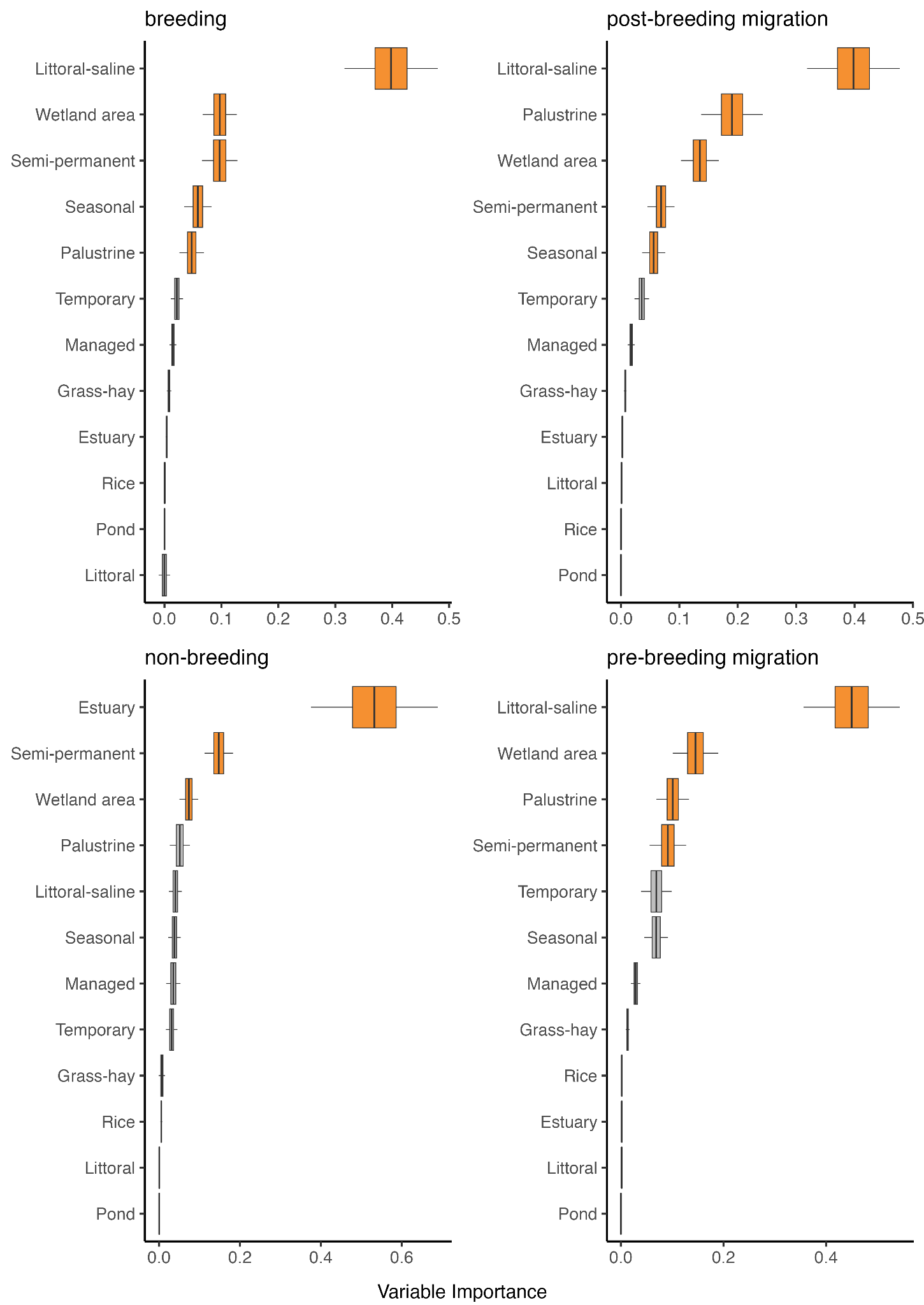


Figures S7. American avocet—explanation of results as referenced previously.


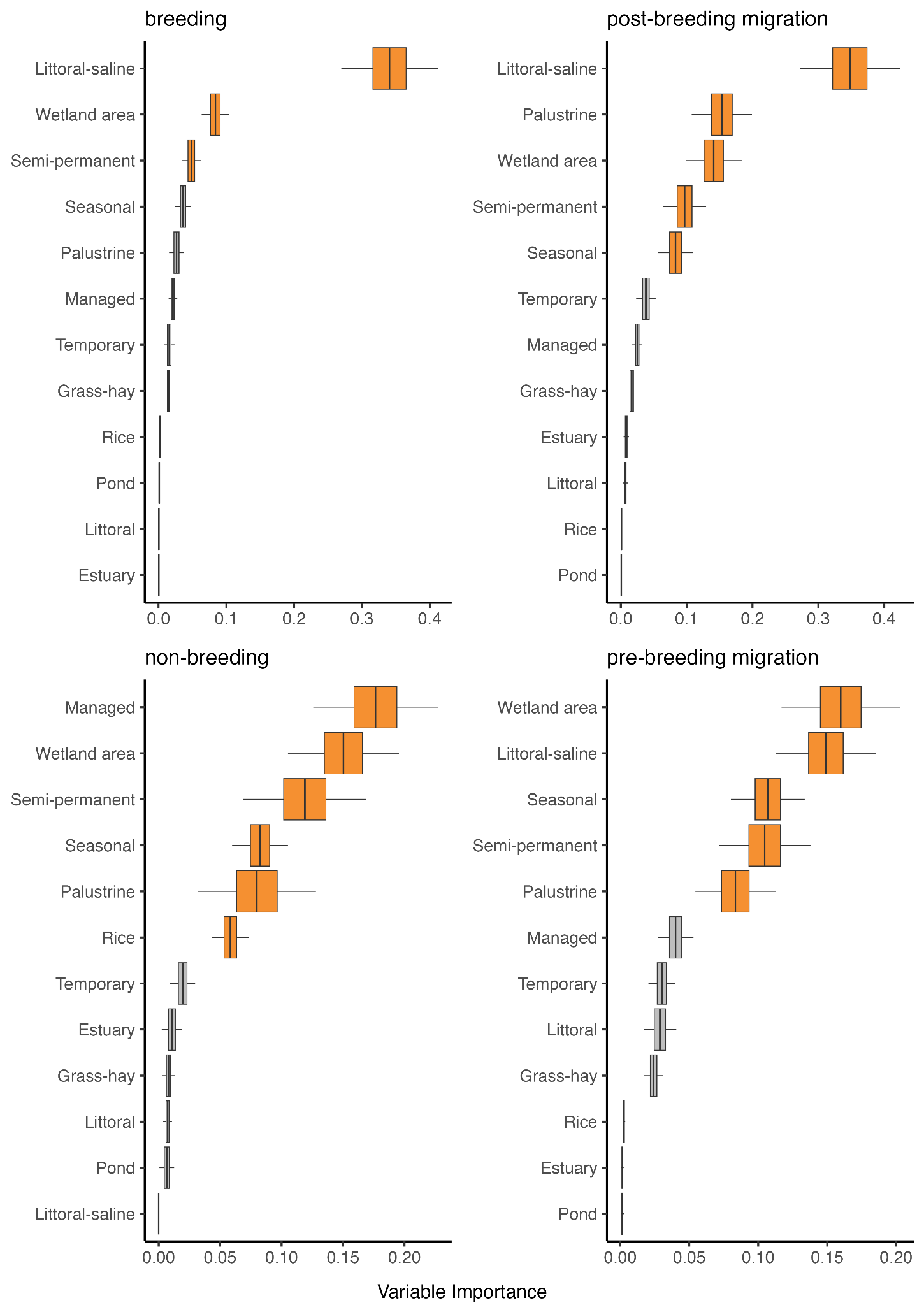


Figures S8. Black-necked stilt—explanation of results as referenced previously.


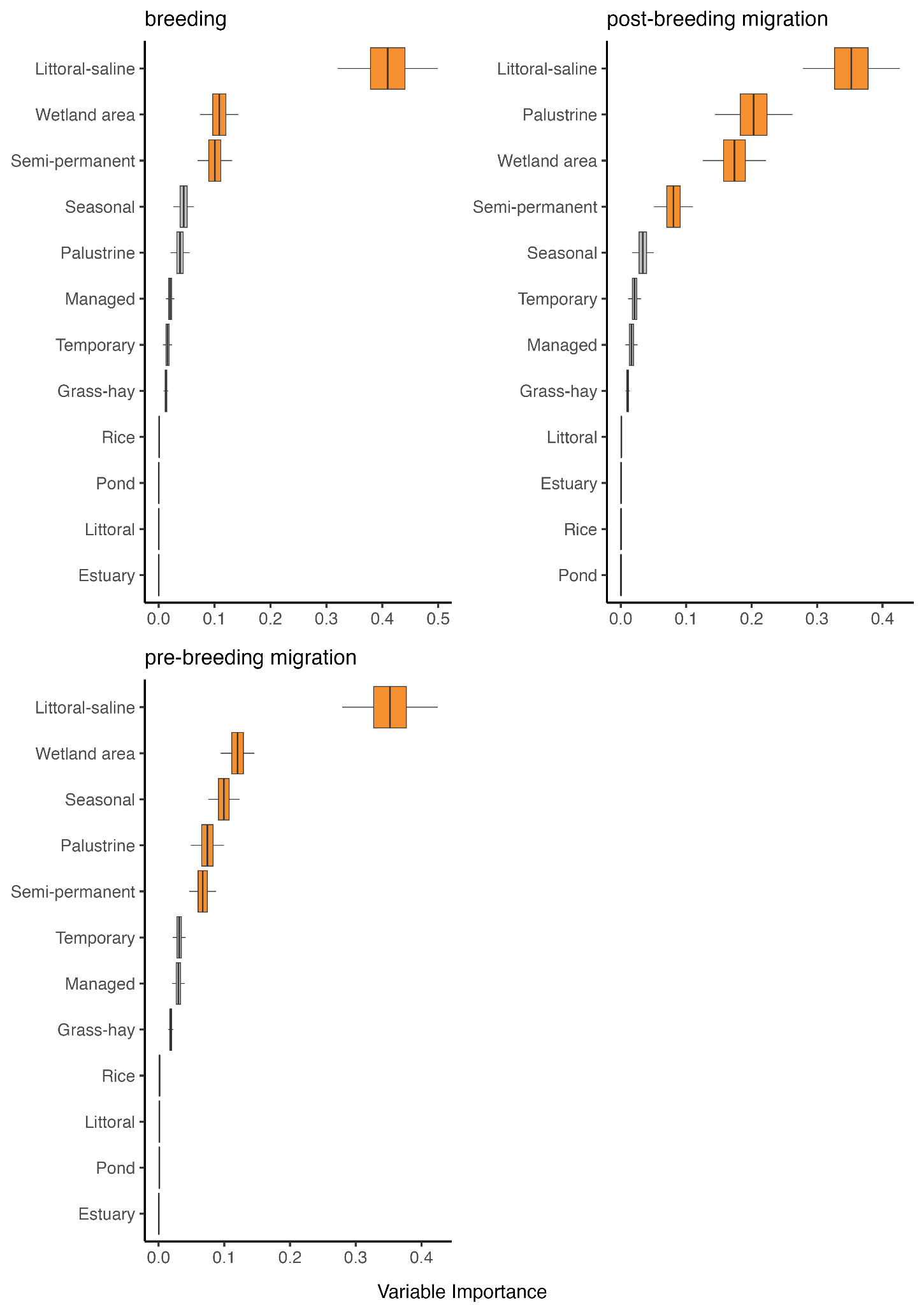


Figure S9. Wilson’s phalarope—explanation of results as referenced previously.


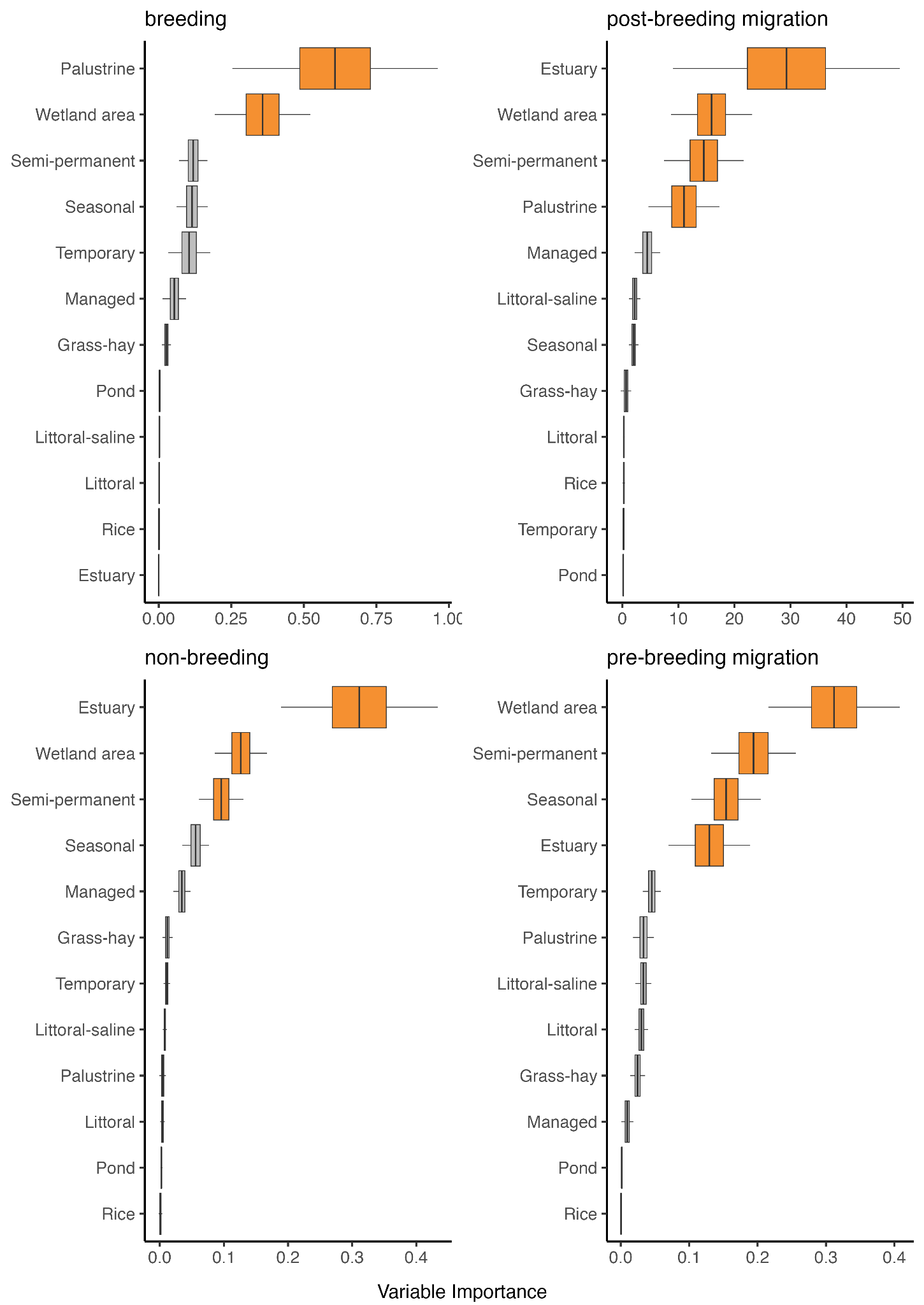


Figures S10. Canvasback—explanation of results as referenced previously.


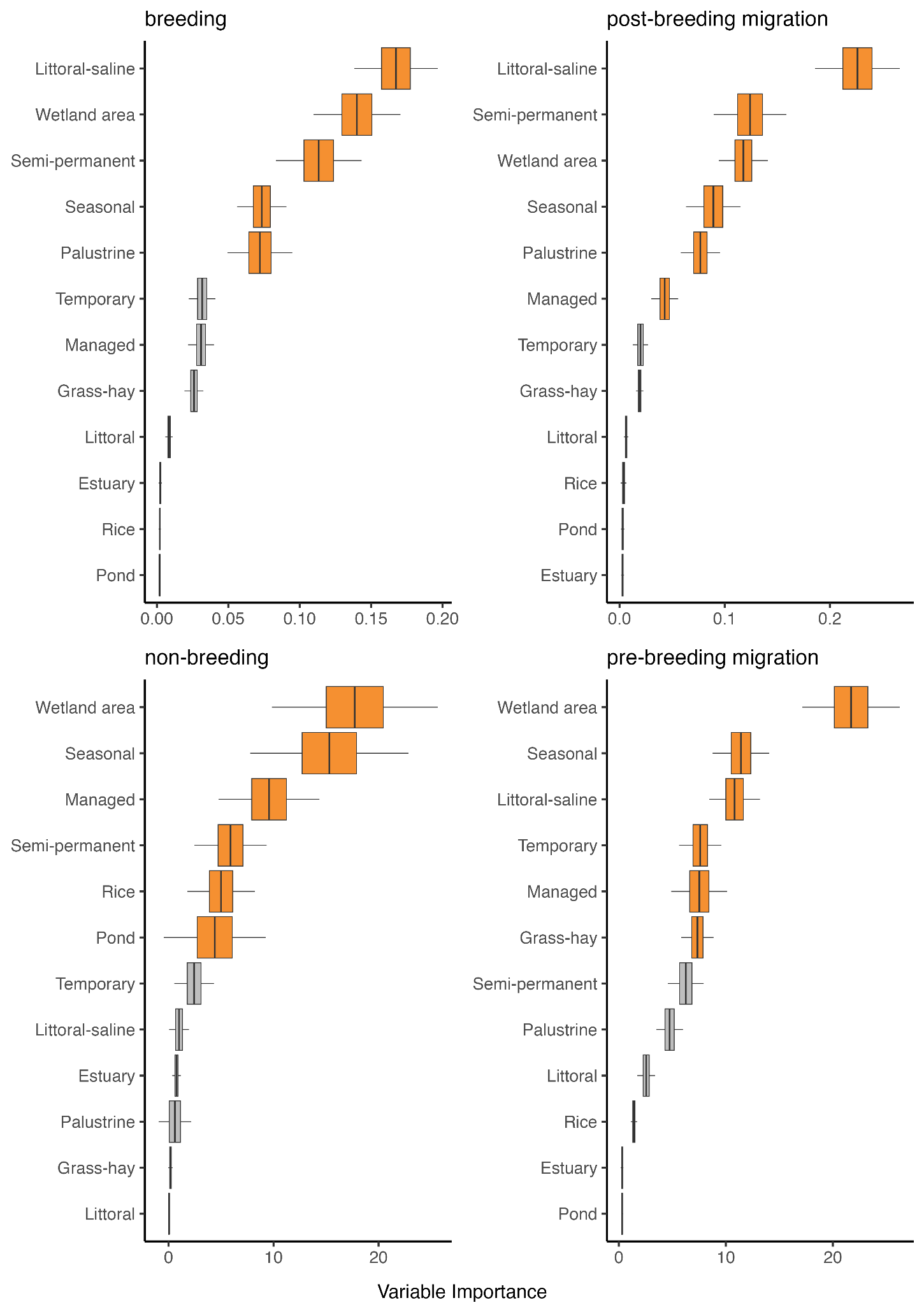


Figure S11. Cinnamon teal—explanation of results as referenced previously.


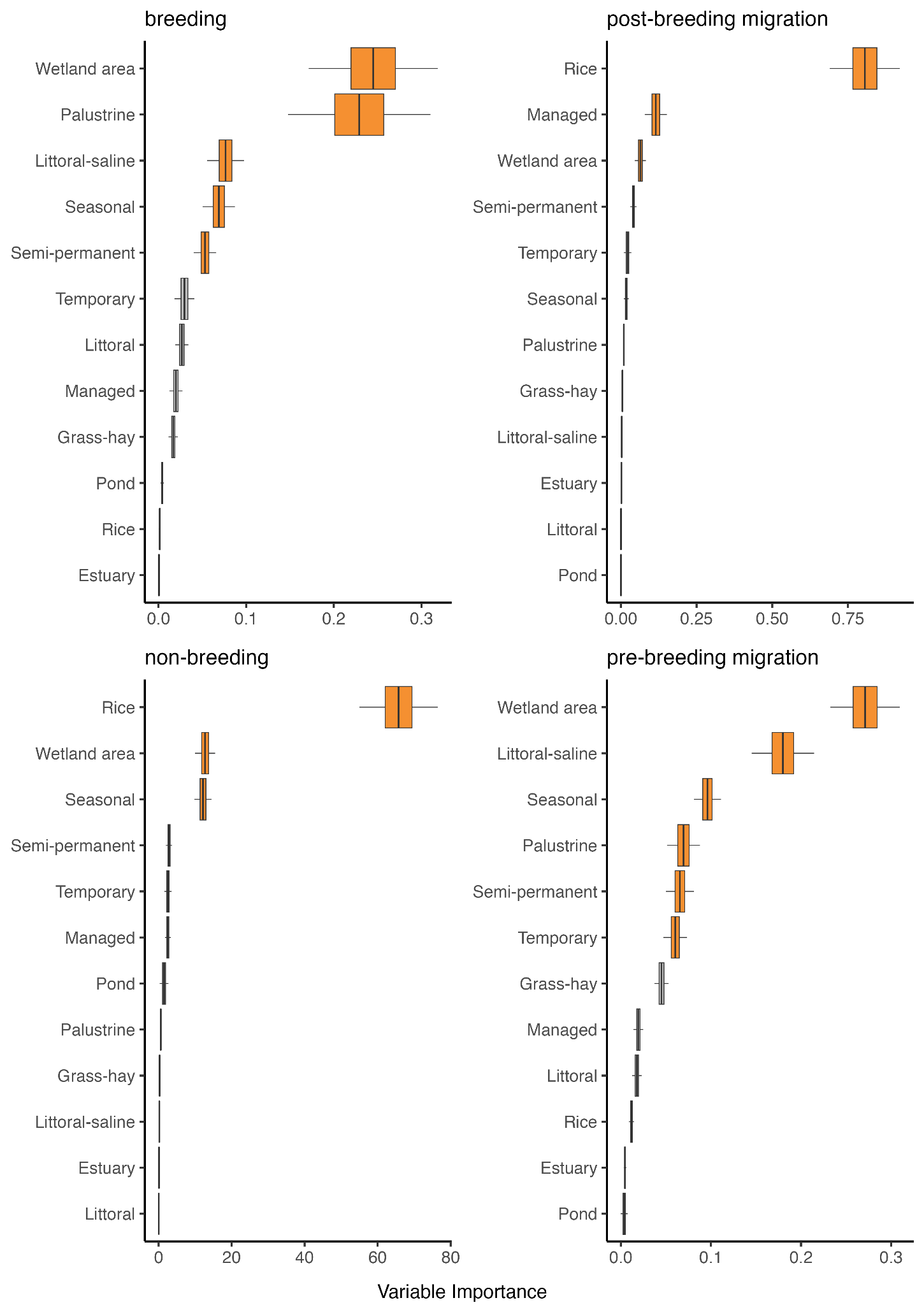


Figure S12. Northern pintail—explanation of results as referenced previously.

1.3 Partial dependence plots

Figures S13-S35 are partial dependence plots depicting marginal effects of wetland variables on relative bird abundance by species (y-axis) and seasonal life-history. Lower-ranking variables interpreted as having a limited effect in structuring bird abundance were excluded. Wetland density measured as ha/2.5km^2^ (x-axis). Gray ribbon denotes 95% confidence interval.


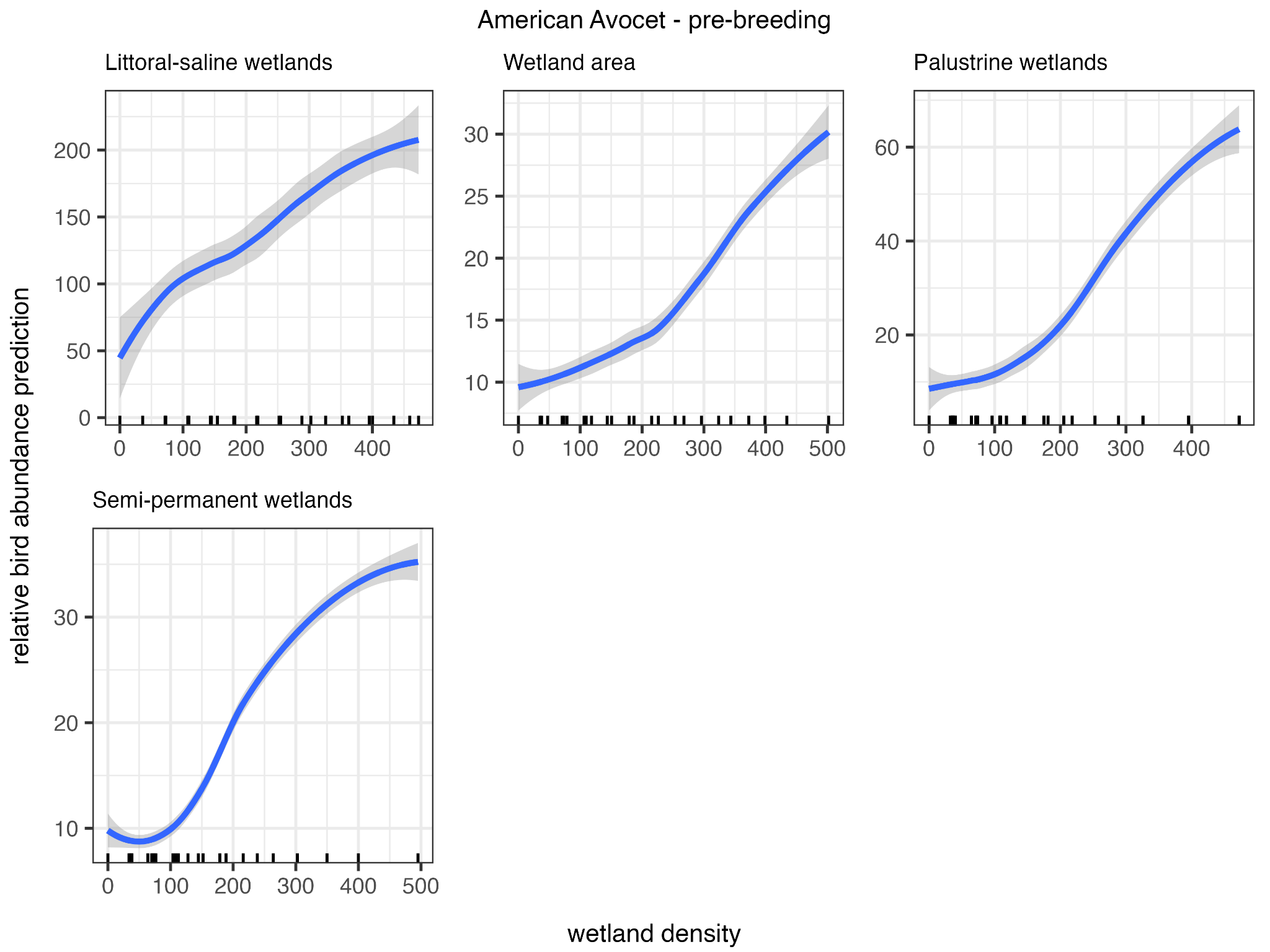


Figure S13 American avocet - breeding—explanation of results as referenced previously.


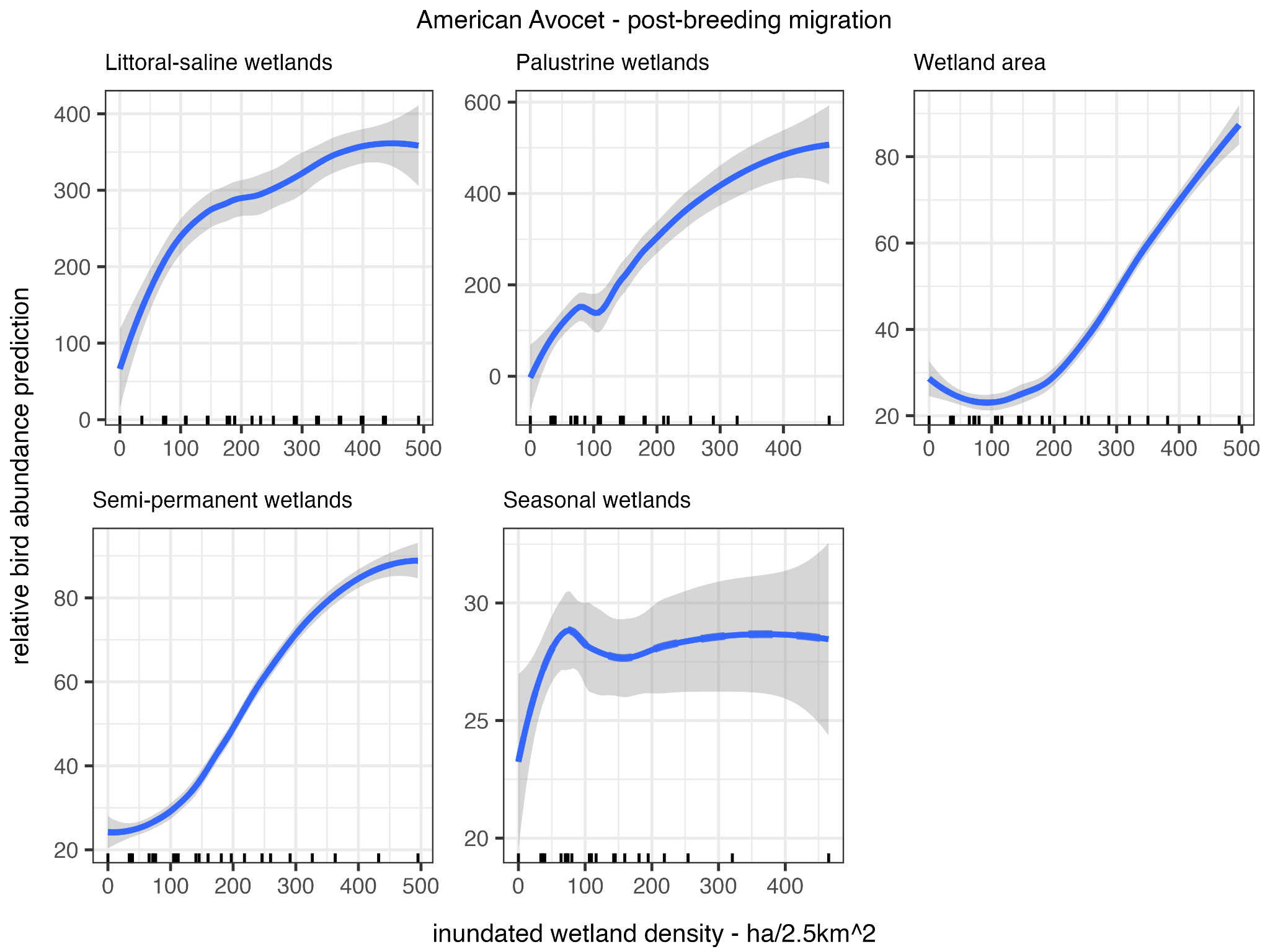


Figure S14 American avocet - post-breeding migration—explanation of results as referenced previously.


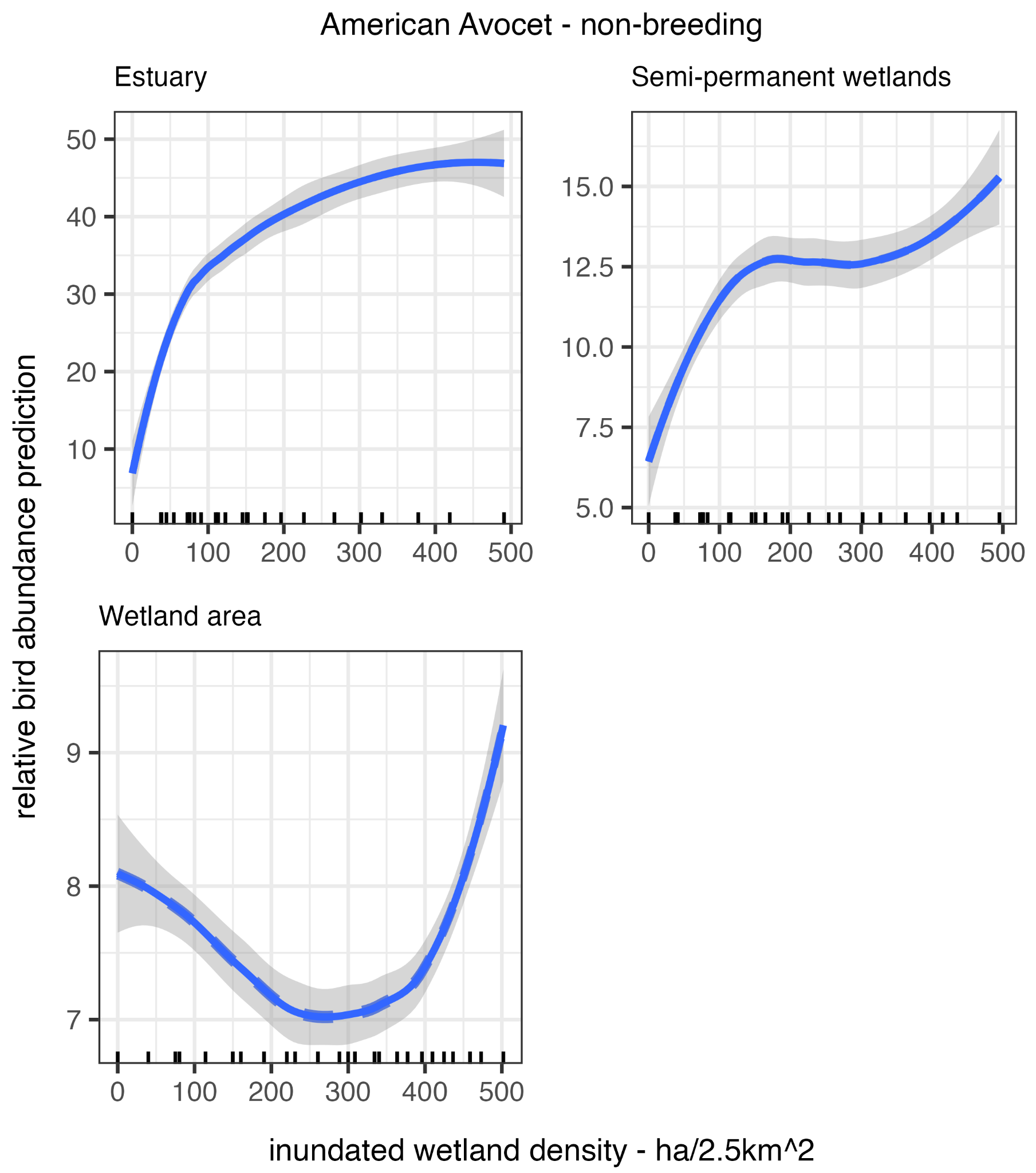


Figure S15 American avocet - non-breeding—explanation of results as referenced previously.


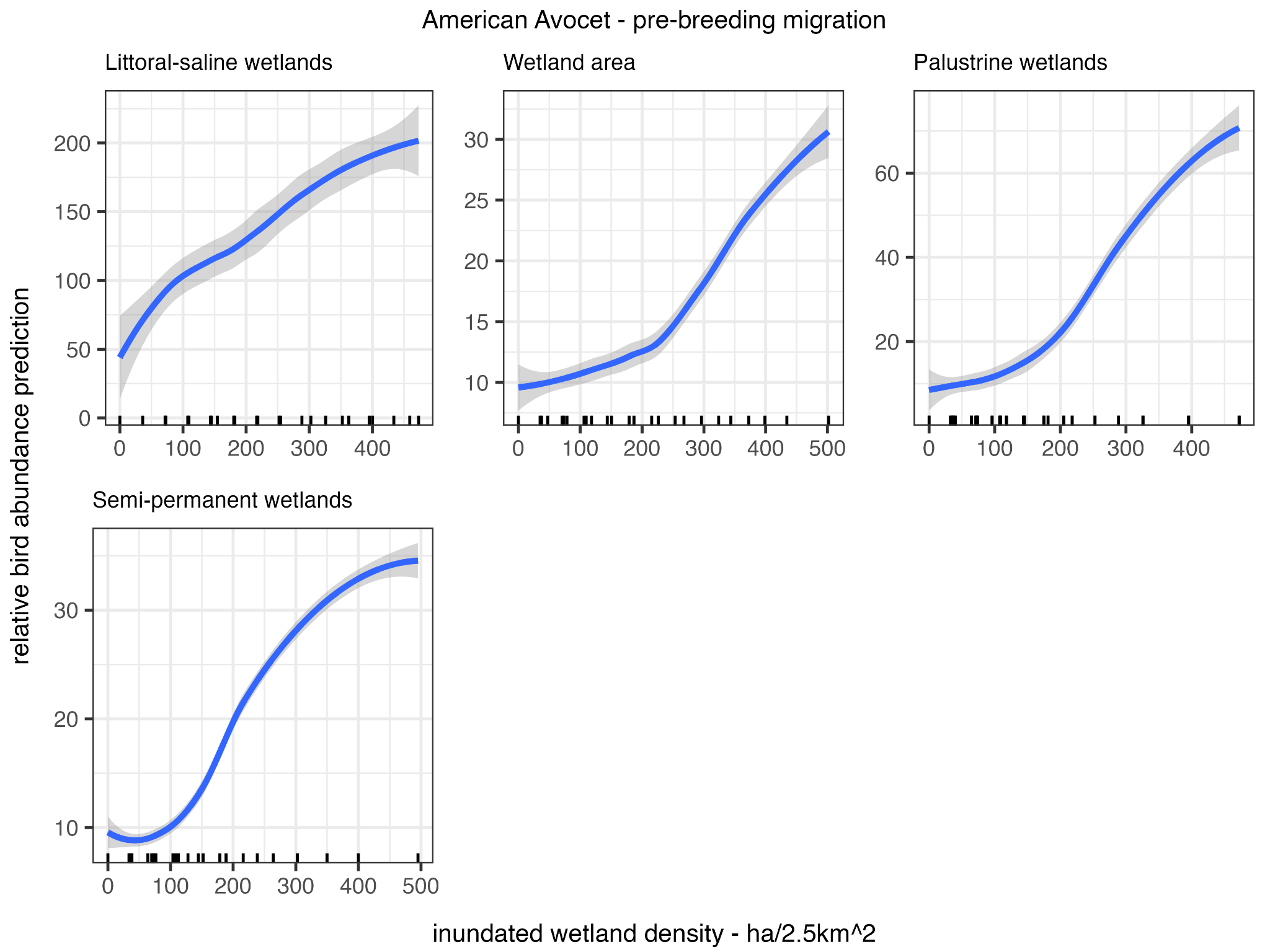


Figure S16 American avocet - pre-breeding migration—explanation of results as referenced previously.


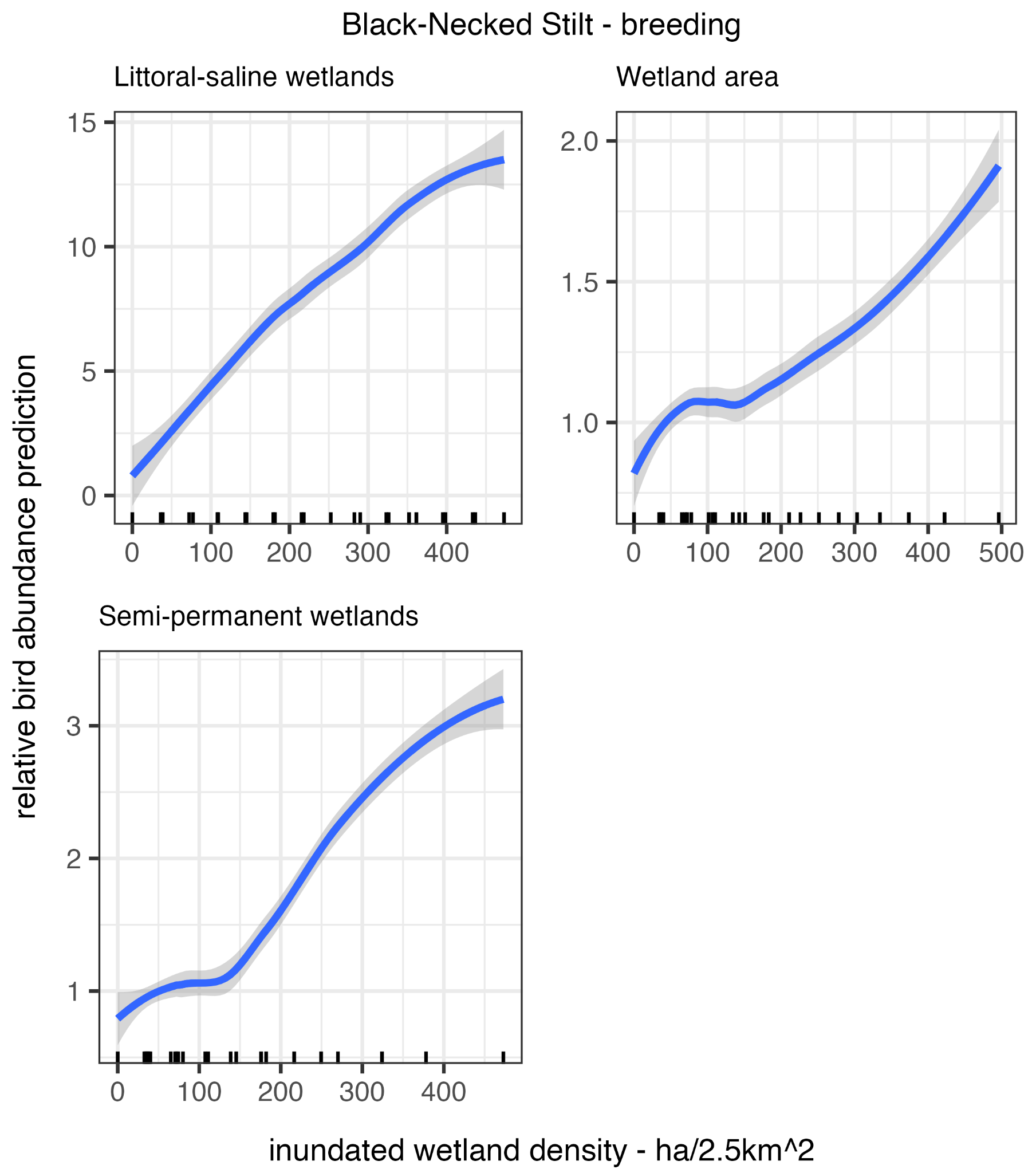


Figure S17 Black-necked stilt - breeding—explanation of results as referenced previously.


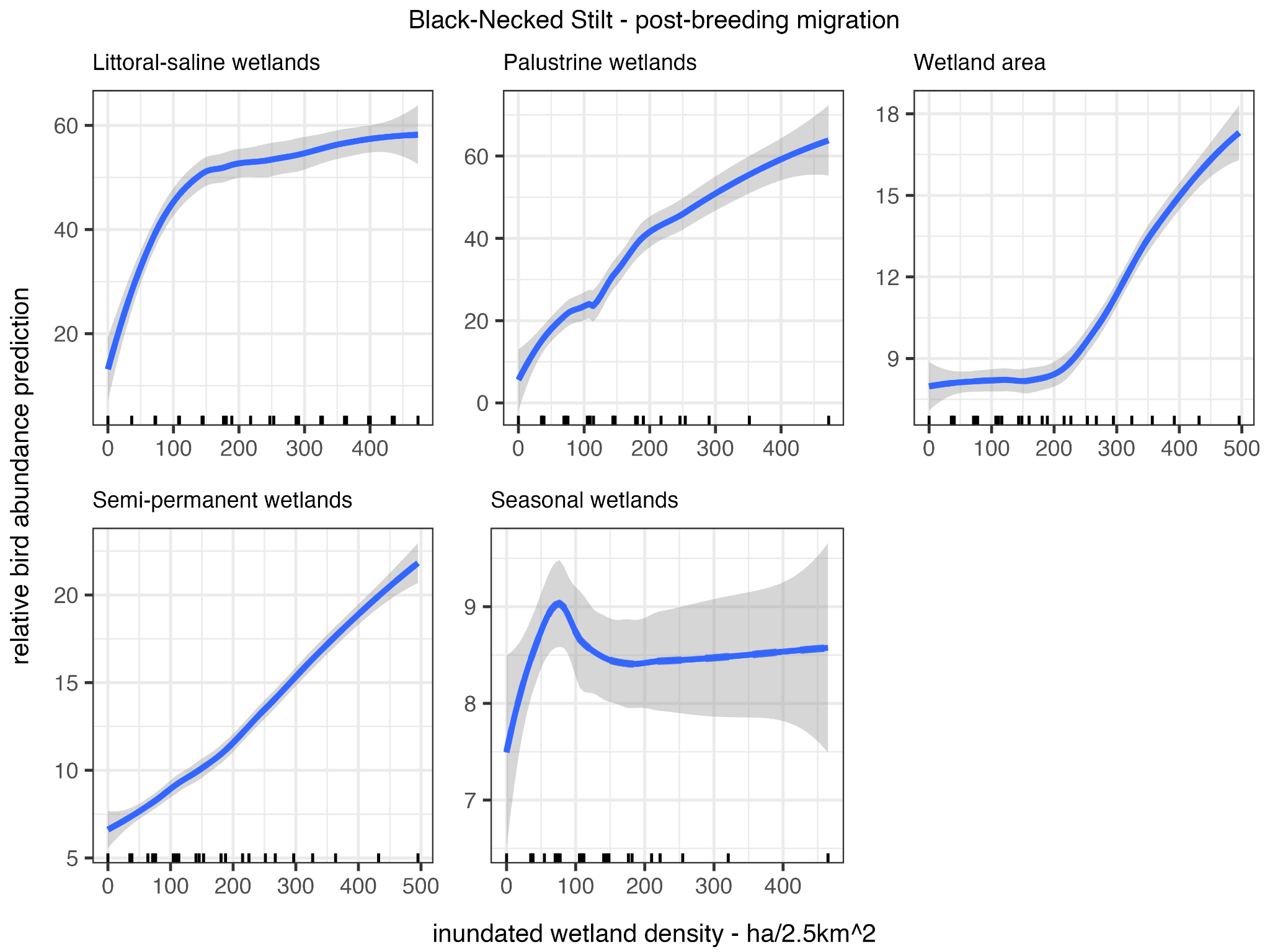


Figure S18 Black-necked stilt - post-breeding migration—explanation of results as referenced previously.


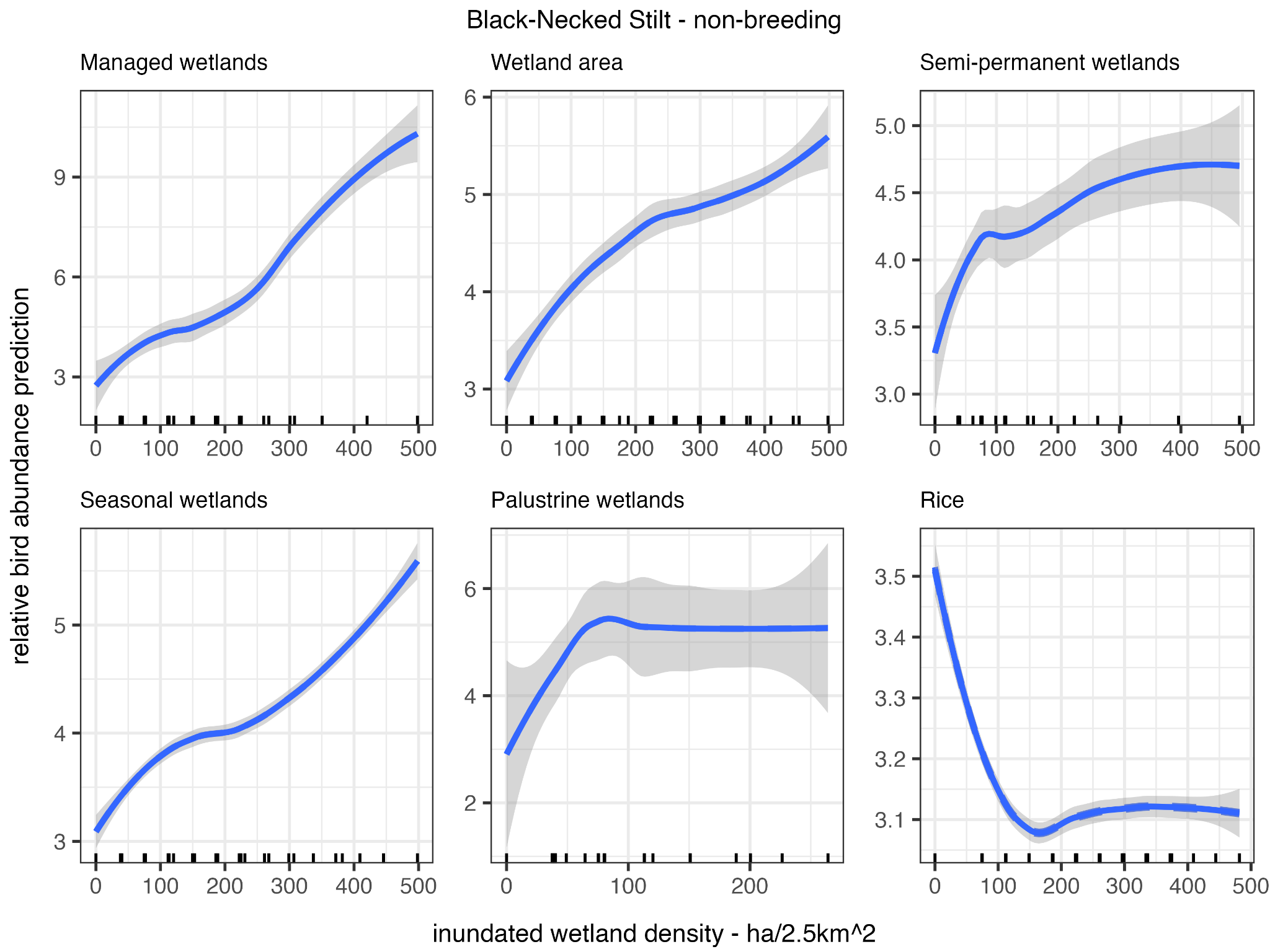


Figure S19 Black-necked stilt - non-breeding—explanation of results as referenced previously.


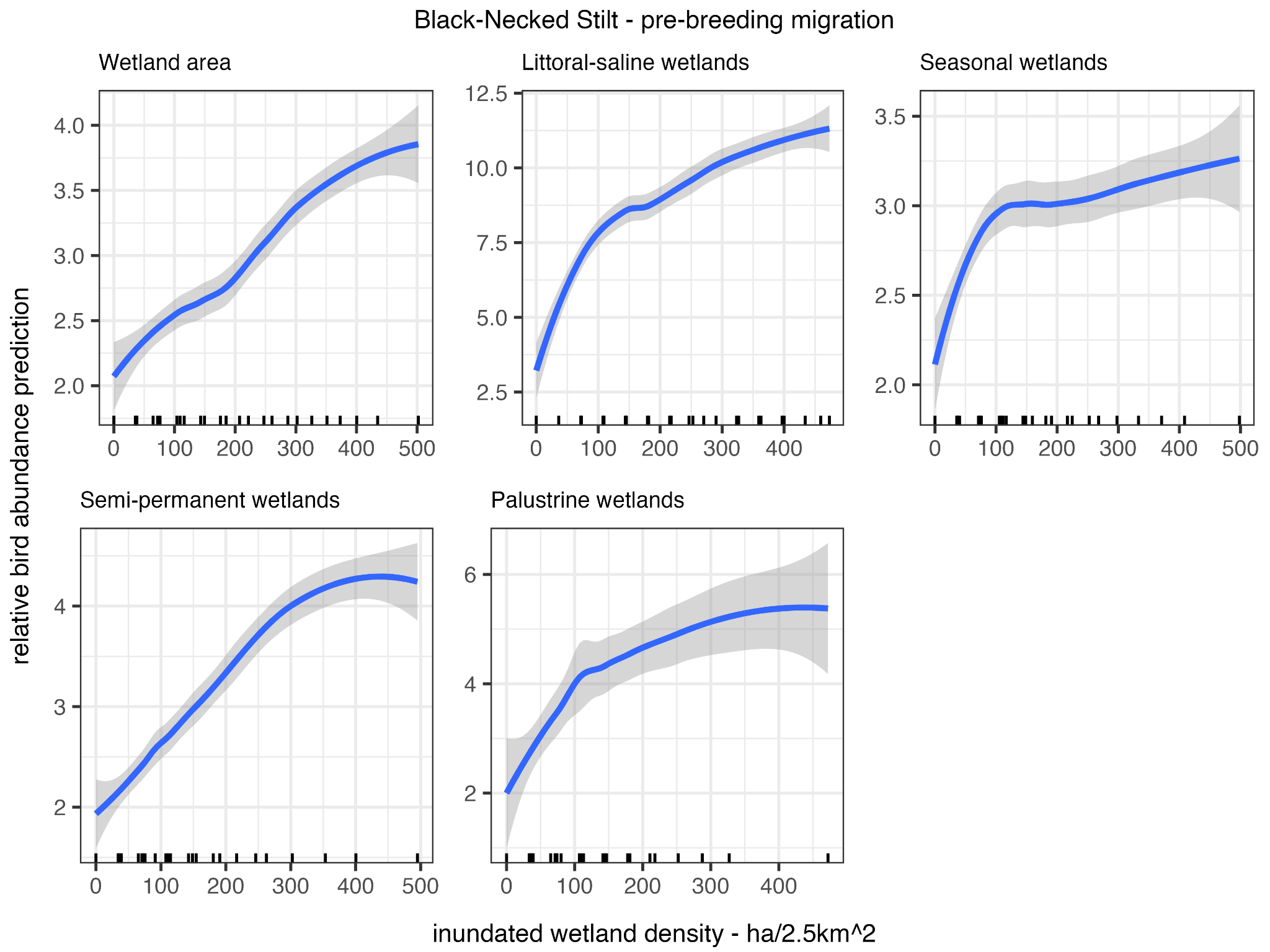


Figure S20 Black-necked stilt - pre-breeding migration—explanation of results as referenced previously.


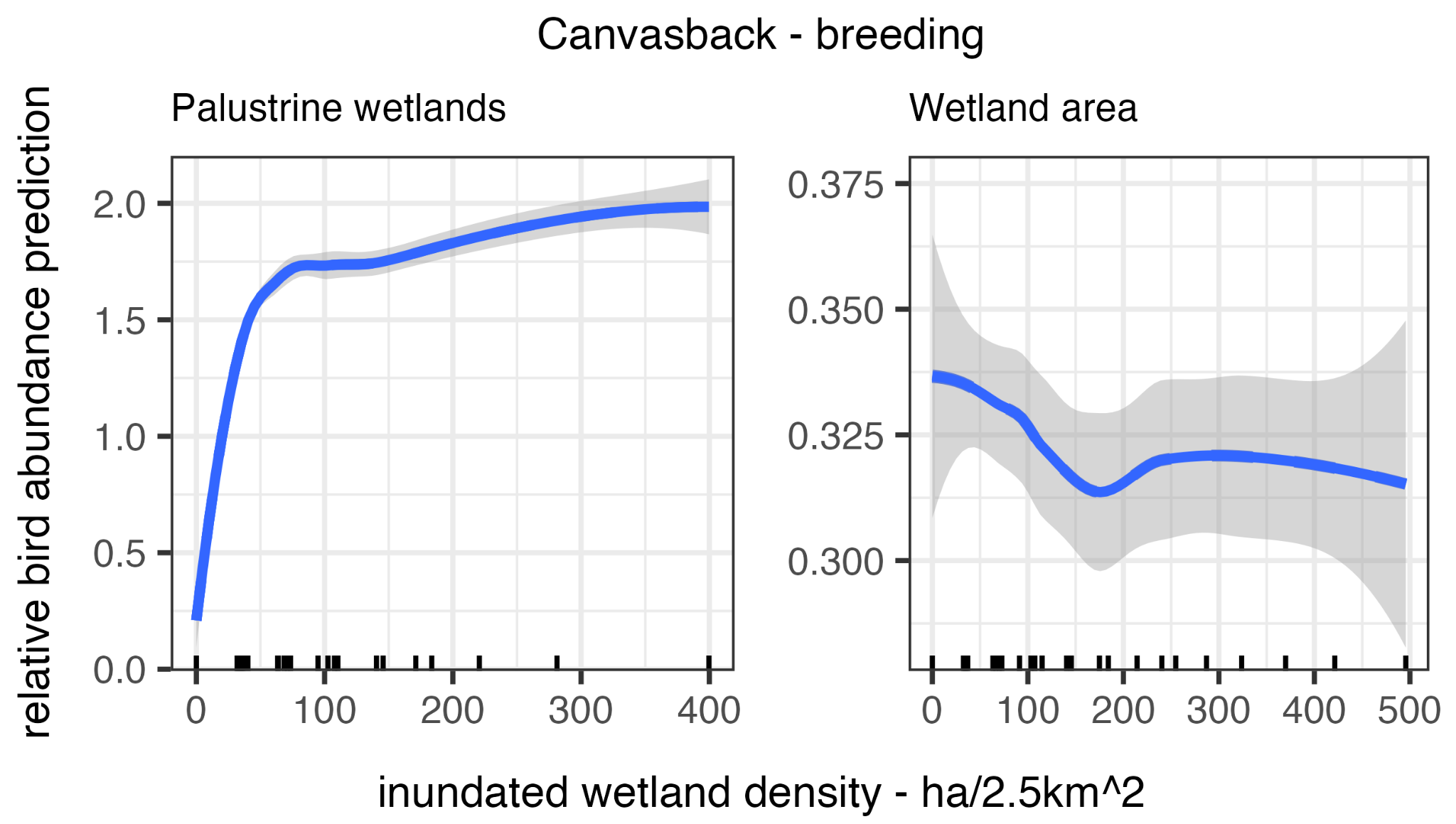


Figure S21 Canvasback - breeding—explanation of results as referenced previously.


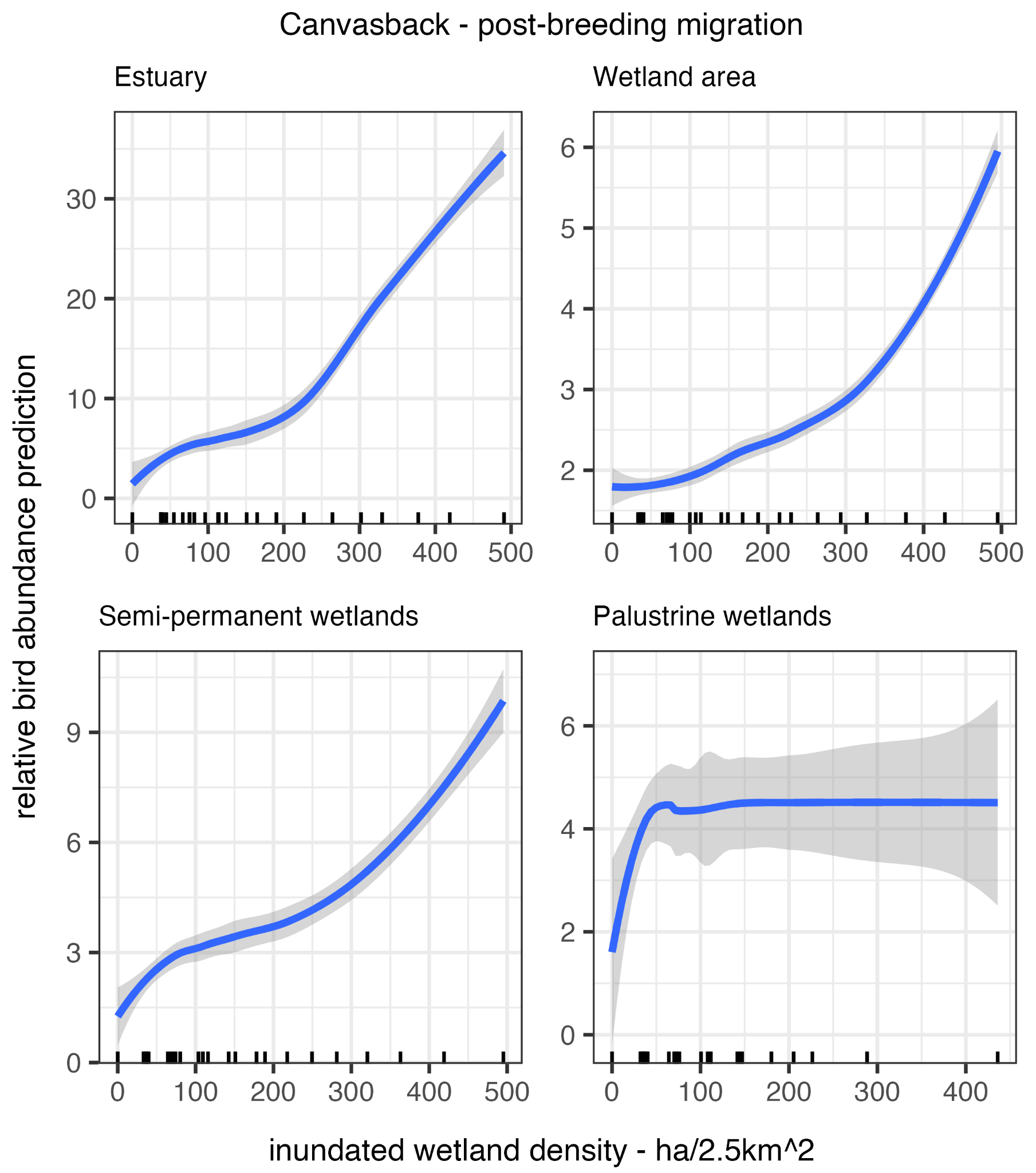


Figure S22 Canvasback - post-breeding migration—explanation of results as referenced previously.


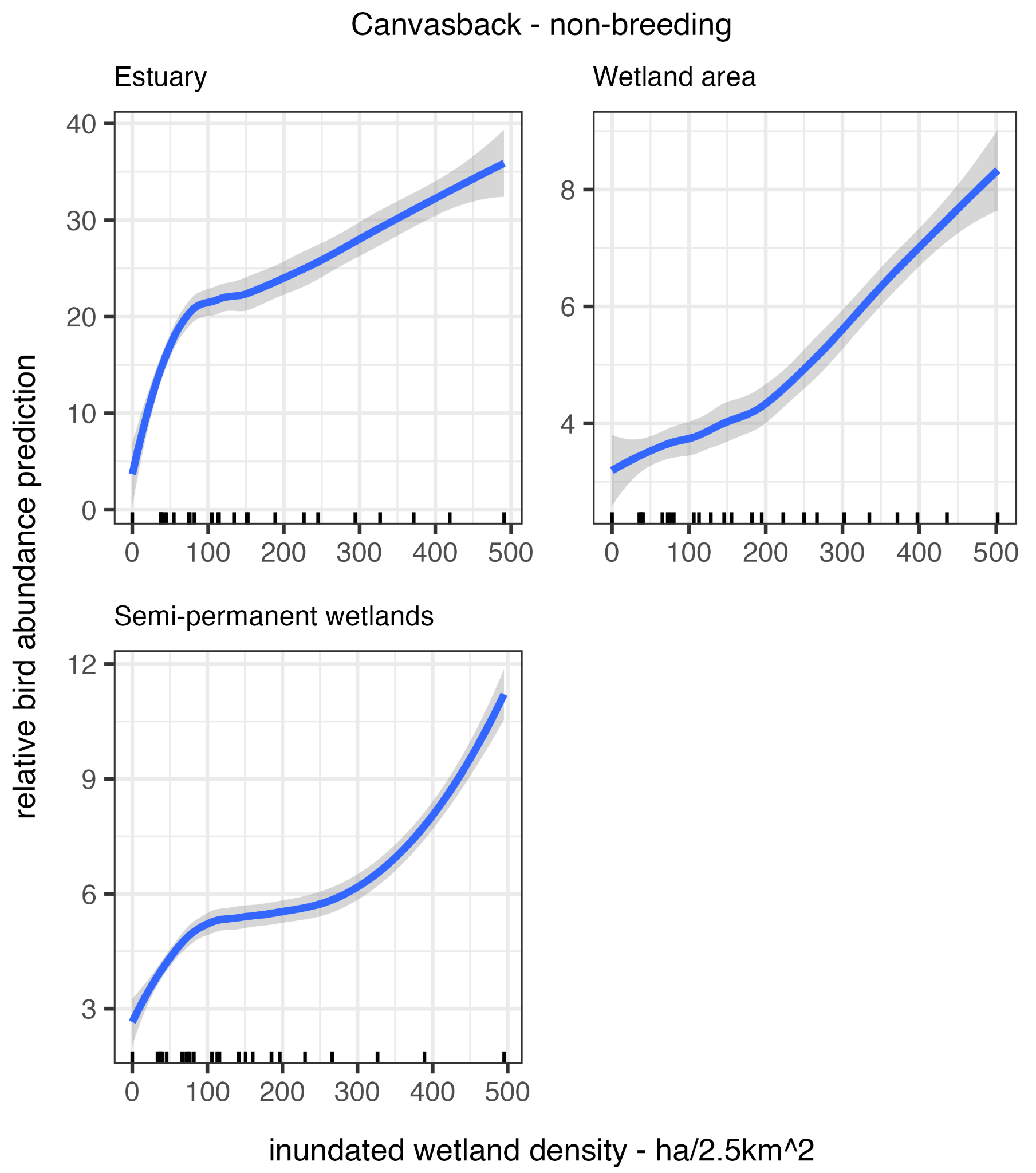


Figure S23 Canvasback - non-breeding—explanation of results as referenced previously.


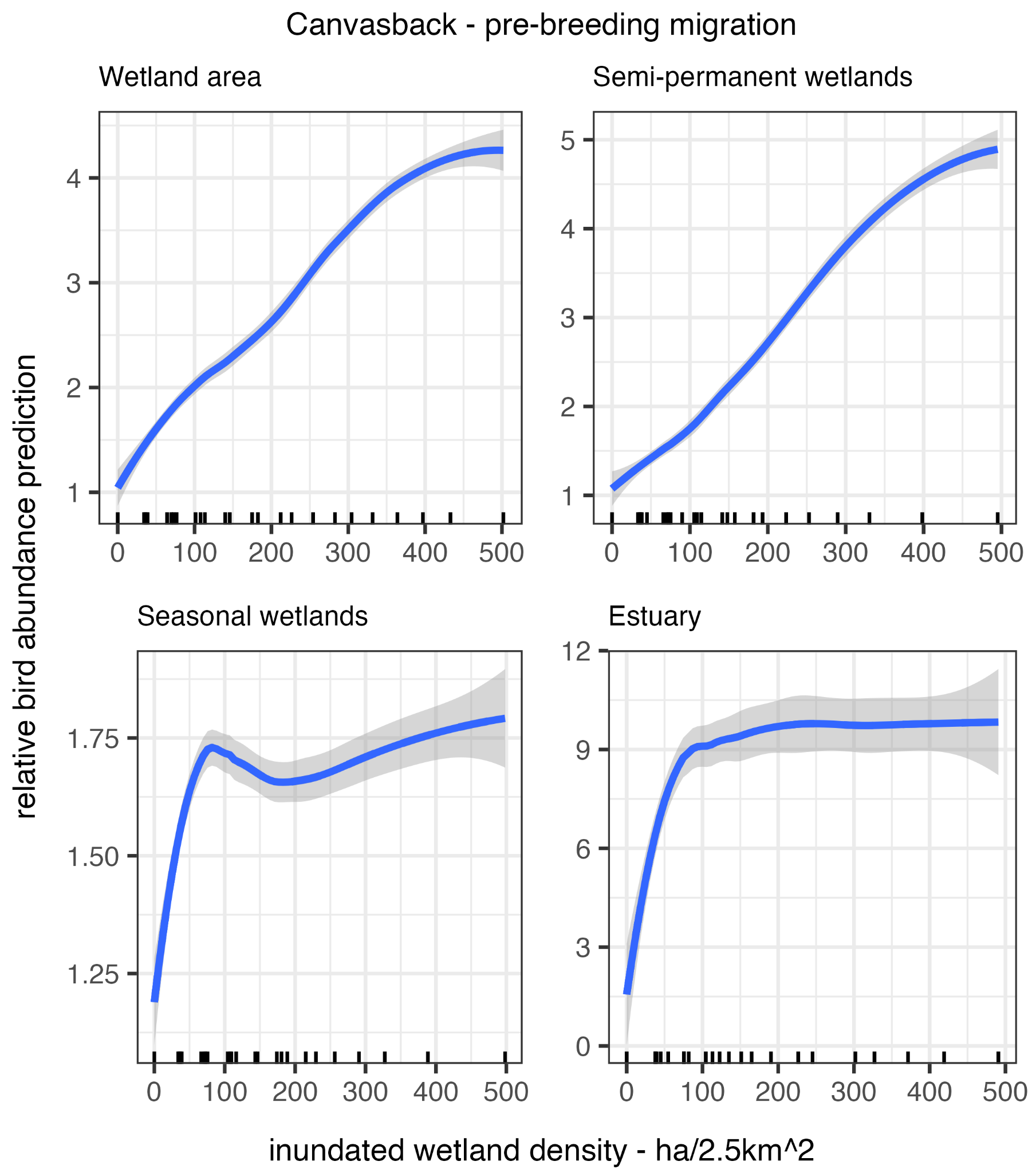


Figure S24 Canvasback - pre-breeding—explanation of results as referenced previously.


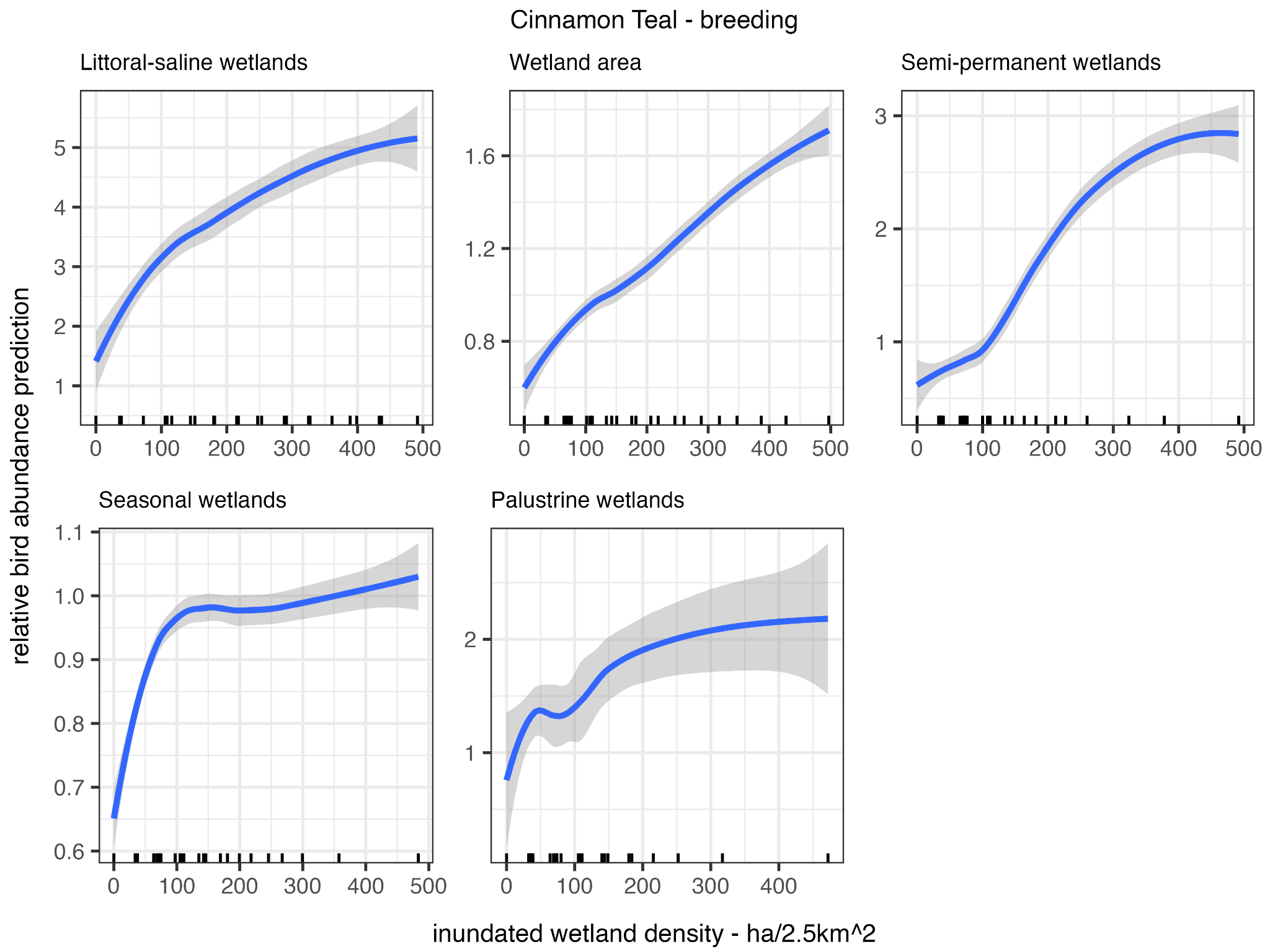


Figure 25 Cinnamon teal - breeding—explanation of results as referenced previously.


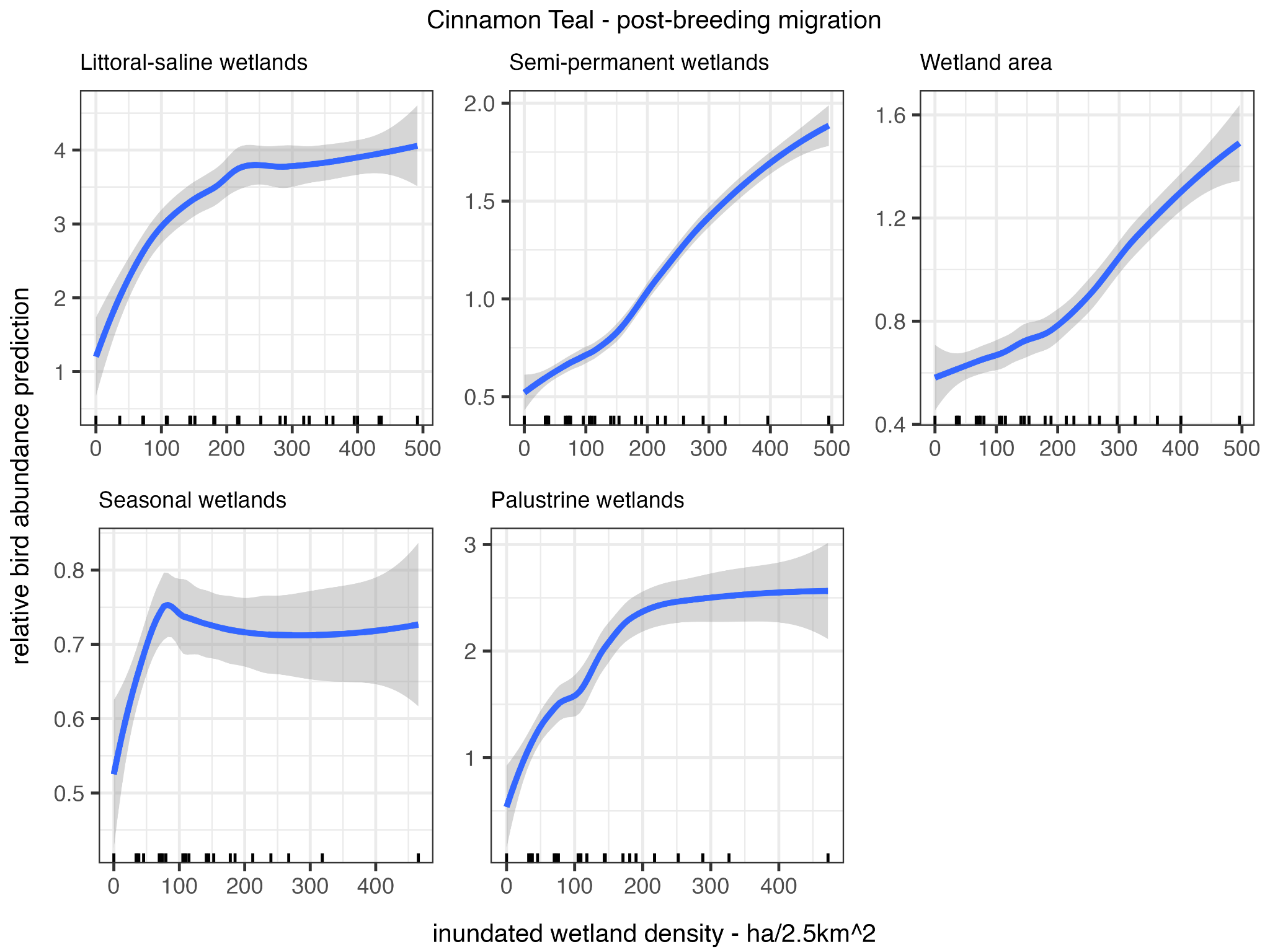


Figure S26 Cinnamon teal - post-breeding migration—explanation of results as referenced previously.


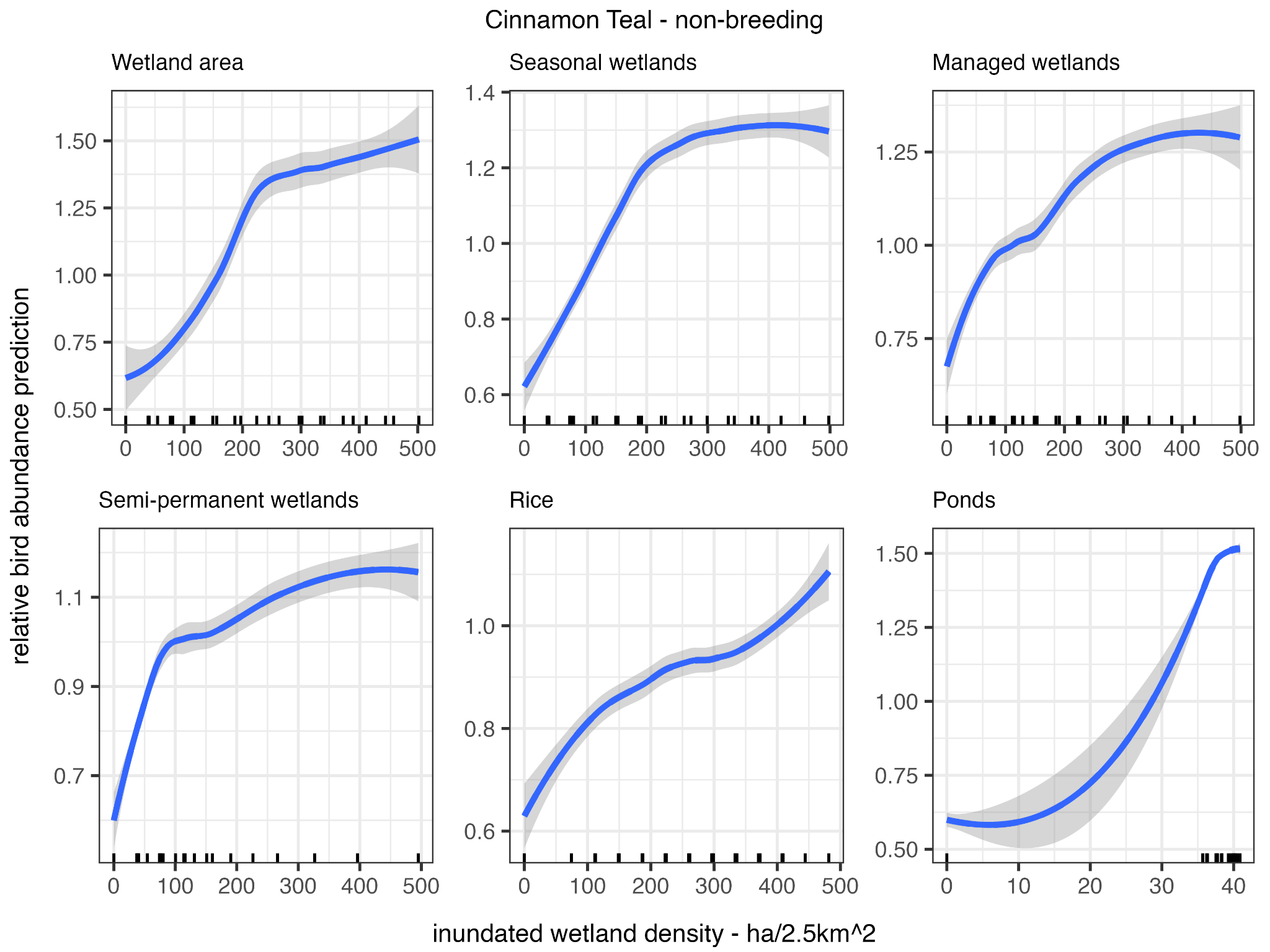


Figure S27 Cinnamon teal - non-breeding—explanation of results as referenced previously.


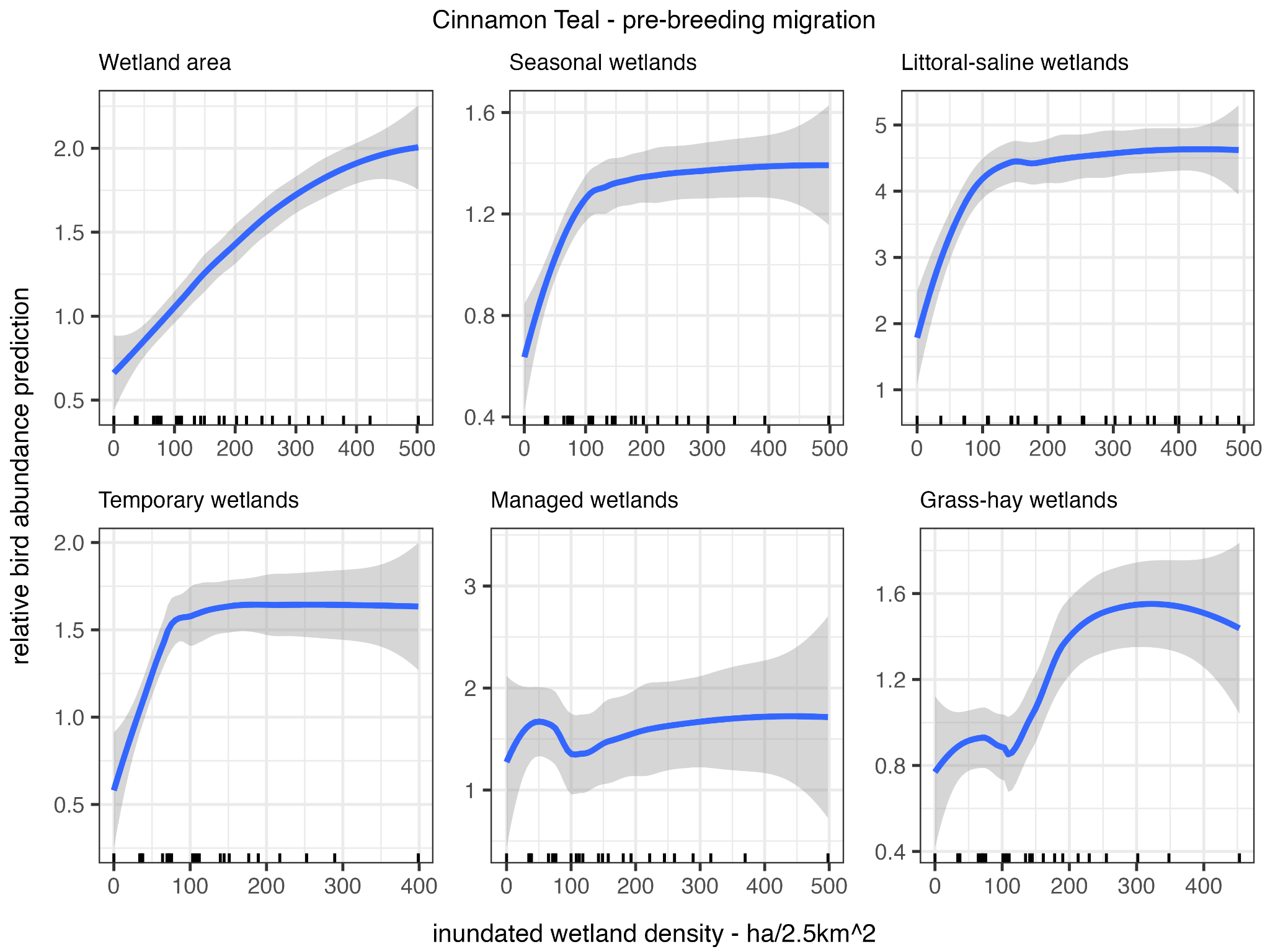


Figure S28 Cinnamon teal - pre-breeding migration—explanation of results as referenced previously.


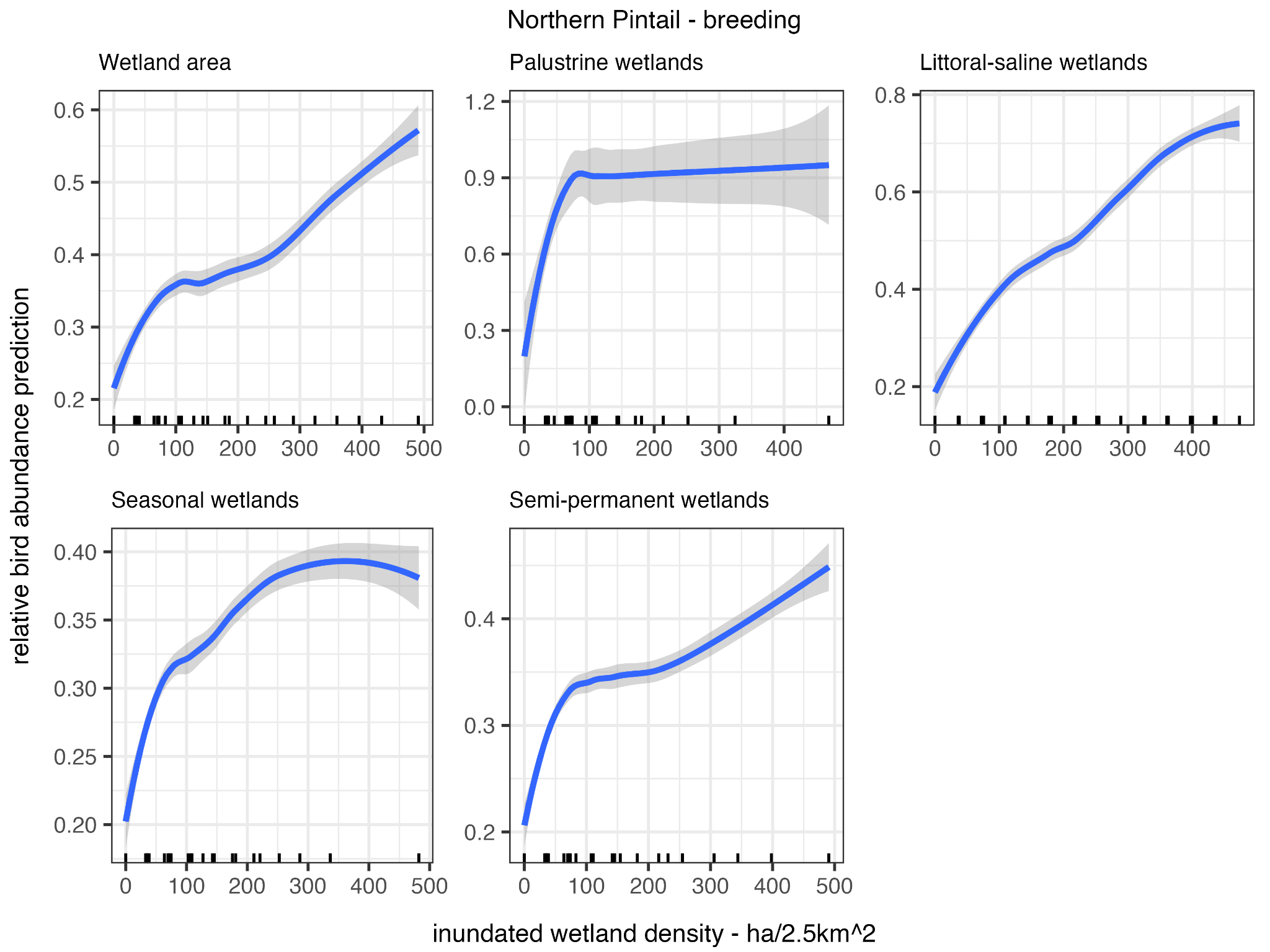


Figure S29 Northern pintail - breeding—explanation of results as referenced previously.


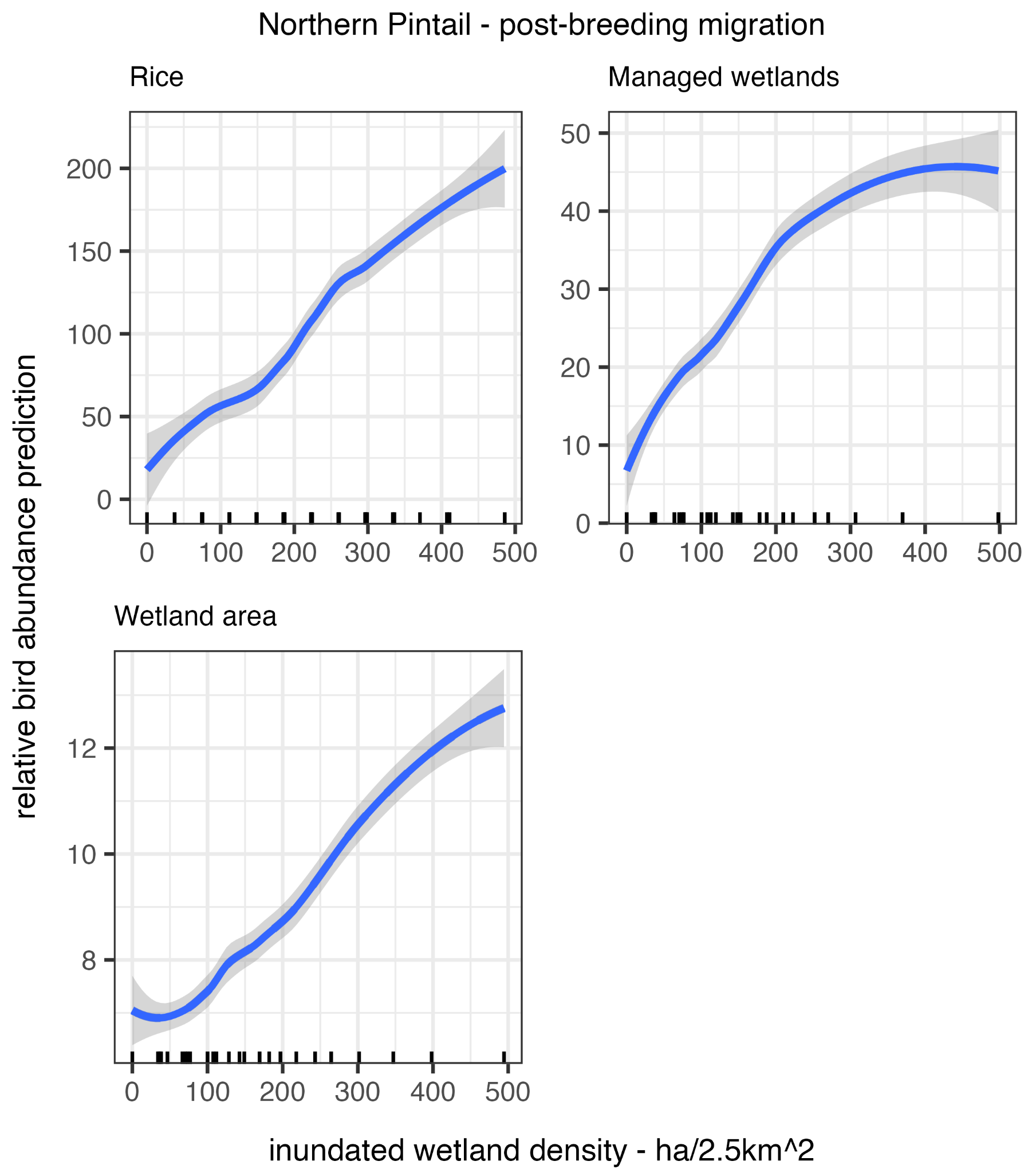


Figure S30 Northern pintail - post-breeding migration—explanation of results as referenced previously.


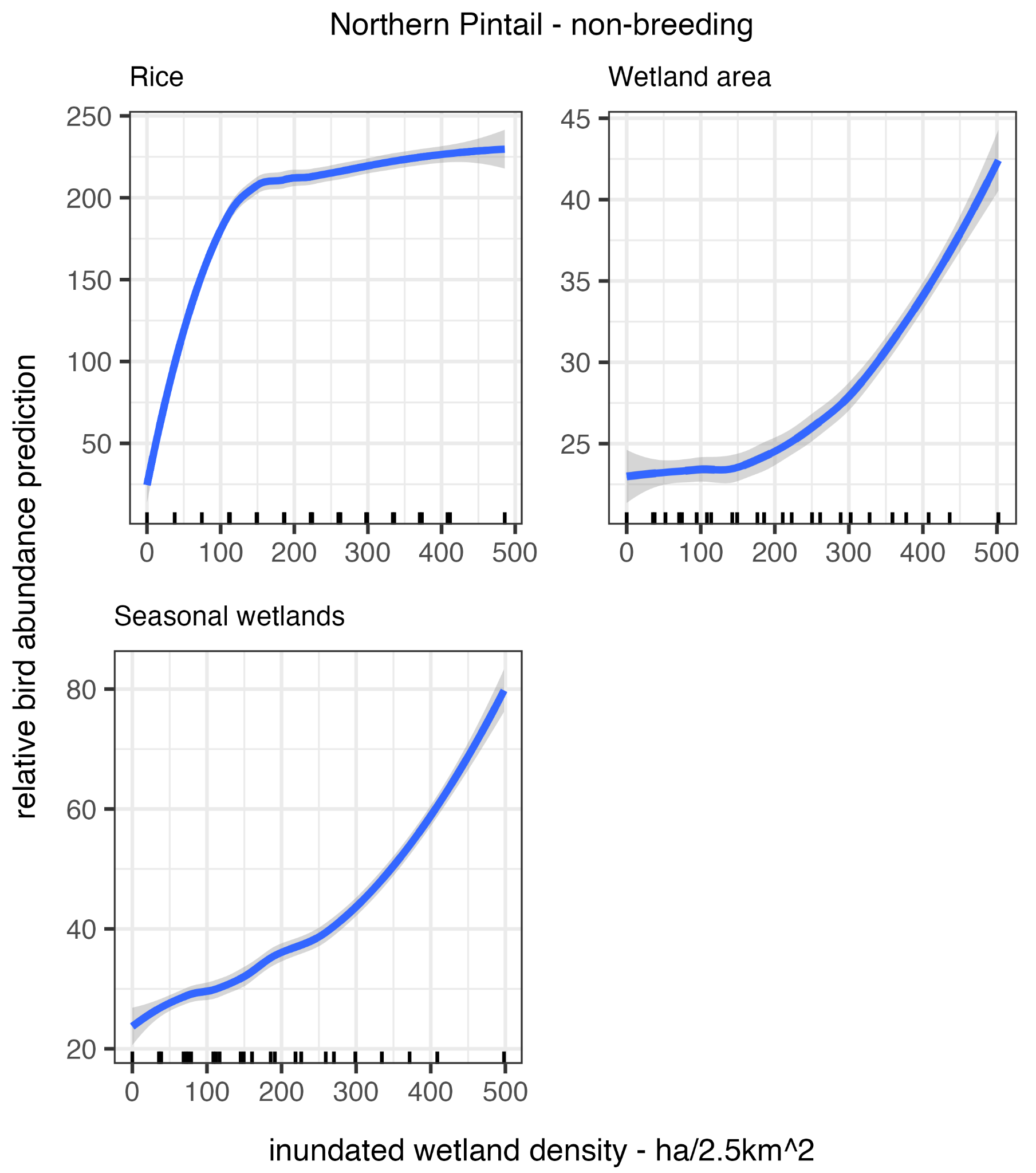


Figure S31 Northern pintail - non-breeding—explanation of results as referenced previously.


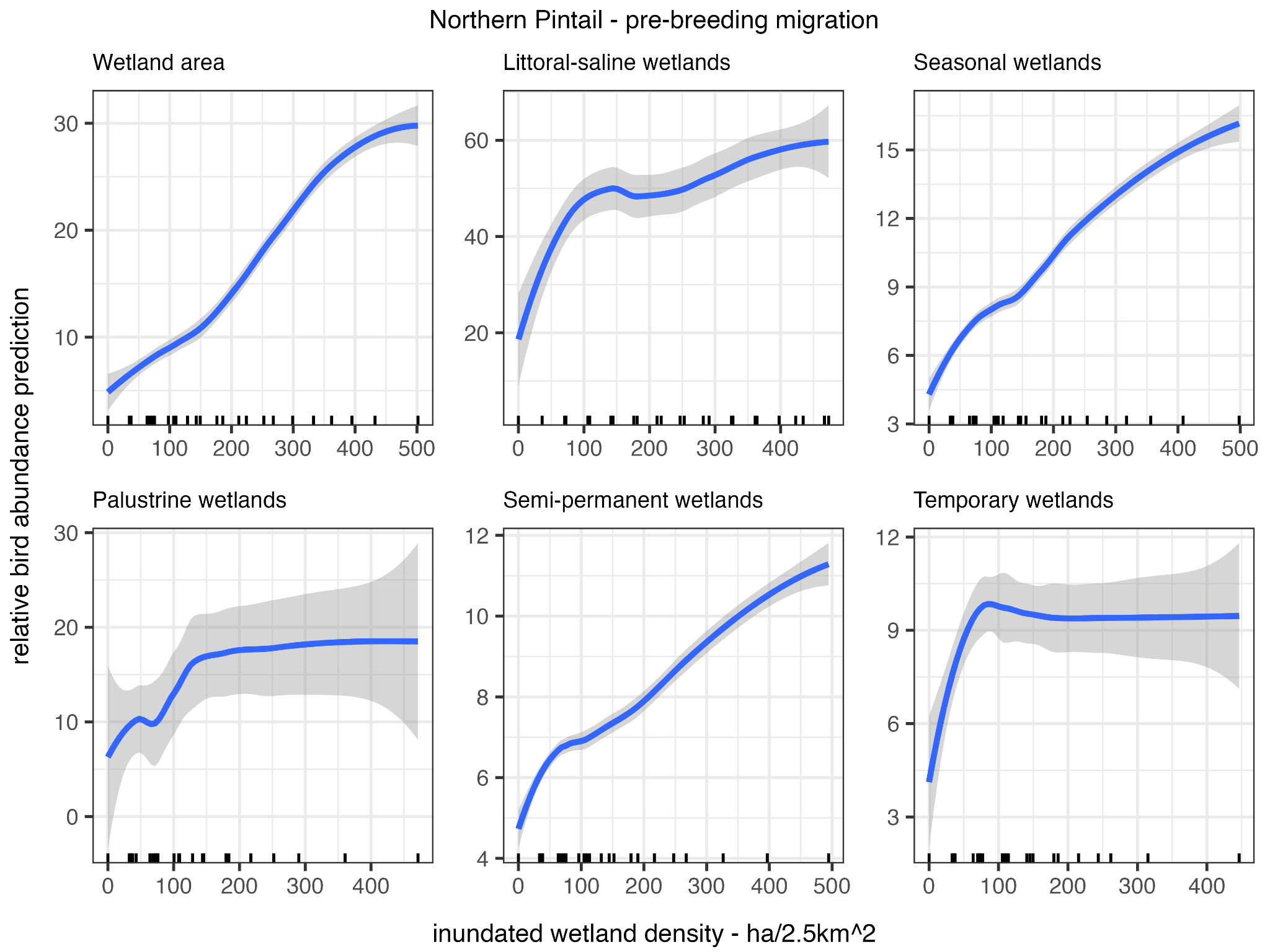


Figure S32 Northern pintail - pre-breeding migration—explanation of results as referenced previously.


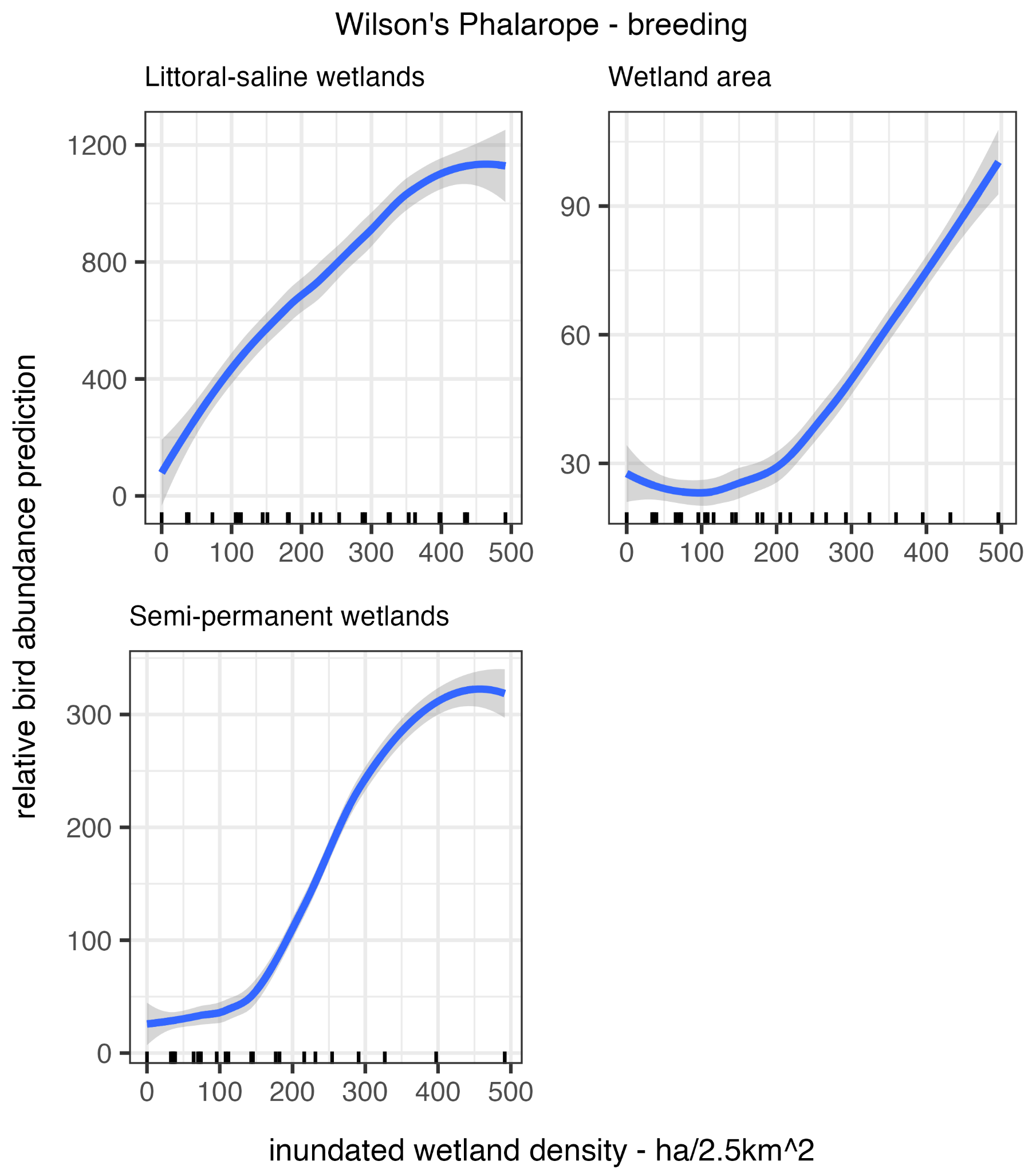


Figure S33 Wilson’s phalarope - breeding—explanation of results as referenced previously.


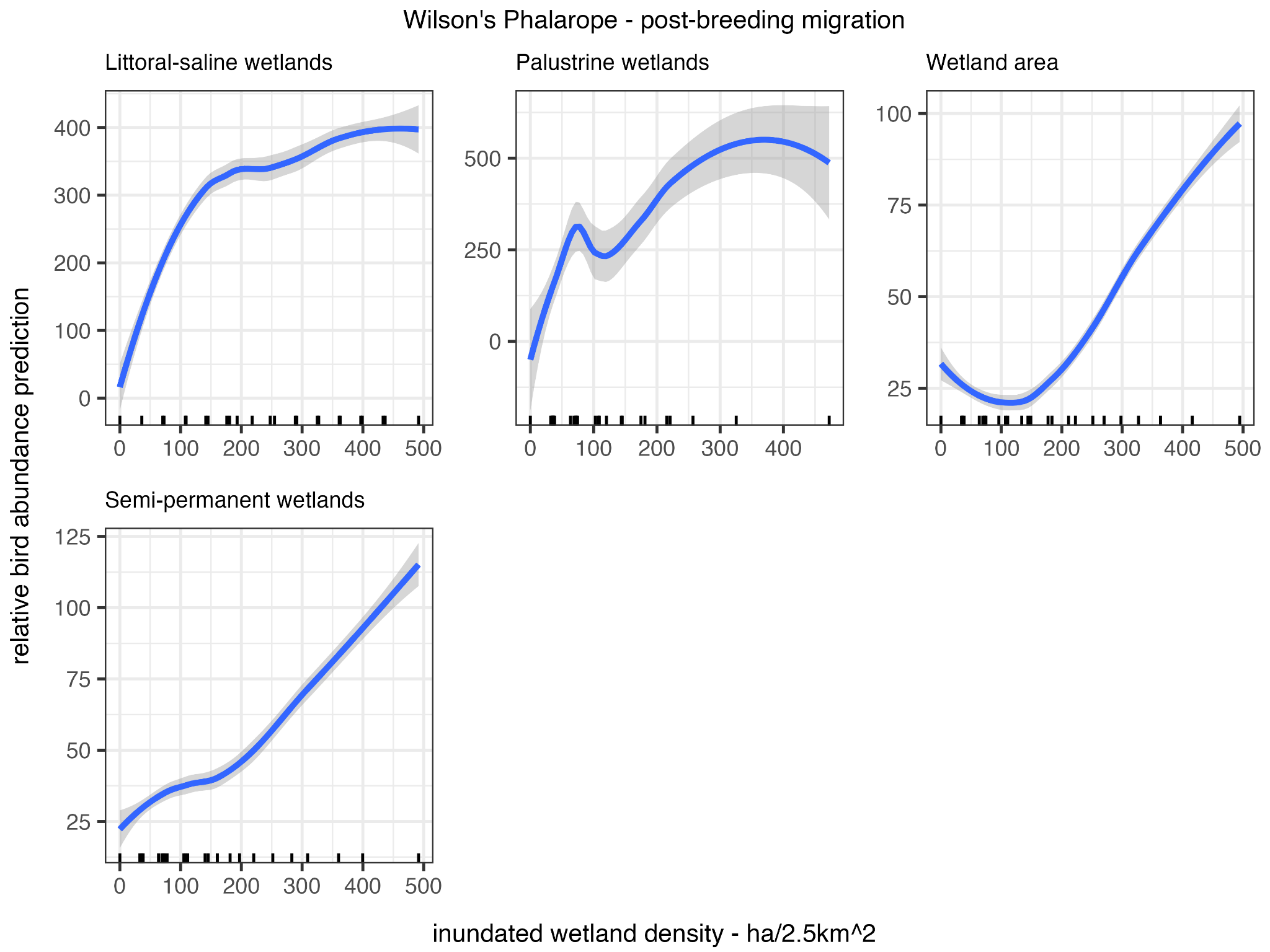


Figure S34 Wilson’s phalarope - post-breeding migration—explanation of results as referenced previously.


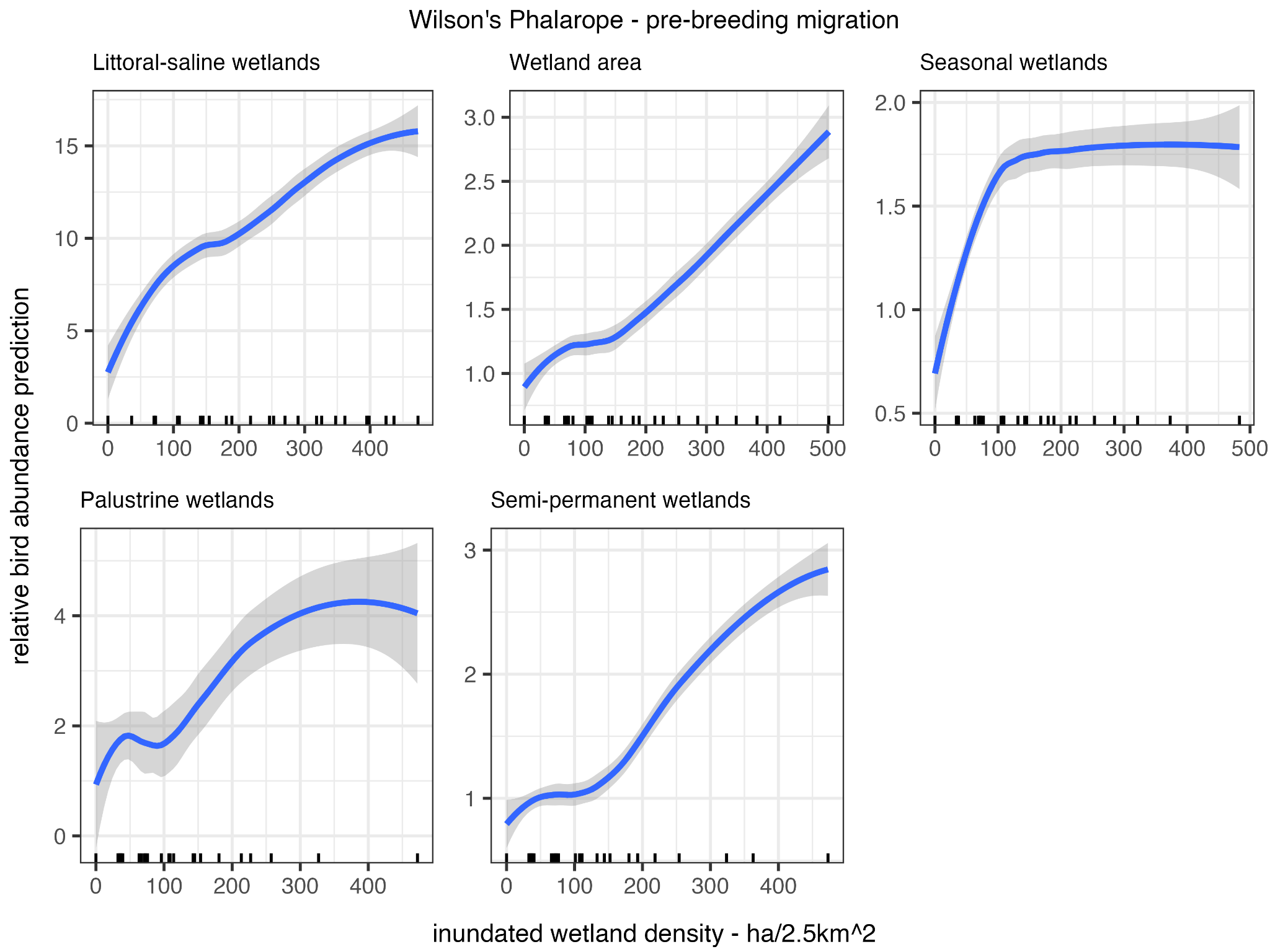


Figure S35 Wilson’s phalarope - pre-breeding migration—explanation of results as referenced previously.

1.4 Waterbird-wetland long-term surface water trends

Figures S36-S41 depict trends in surface water area (kha) for wetland features structuring American avocet, black-necked stilt, canvasback, cinnamon teal, northern pintail, and Wilson’s Phalarope abundance in the western U.S. Wetlands features are presented in order (left to right) of RandomForestSRC variable importance score for each life-history period. Low-scoring features with limited influence on bird abundance were excluded. Measures of wetland surface water change were made between P1 (1984-2003) and P2 (2004-2023) for areas of estimated bird abundance greater than zero. Statistical difference was determined as p-values < 0.1 derived from Wilcoxon ranked order tests. Red indicates significant wetland decline, and blue indicates stable to increasing trends. Boxes, interquartile range (IQR); line dividing the box horizontally, median value; whiskers, 1.5 times the IQR; points, outliers.


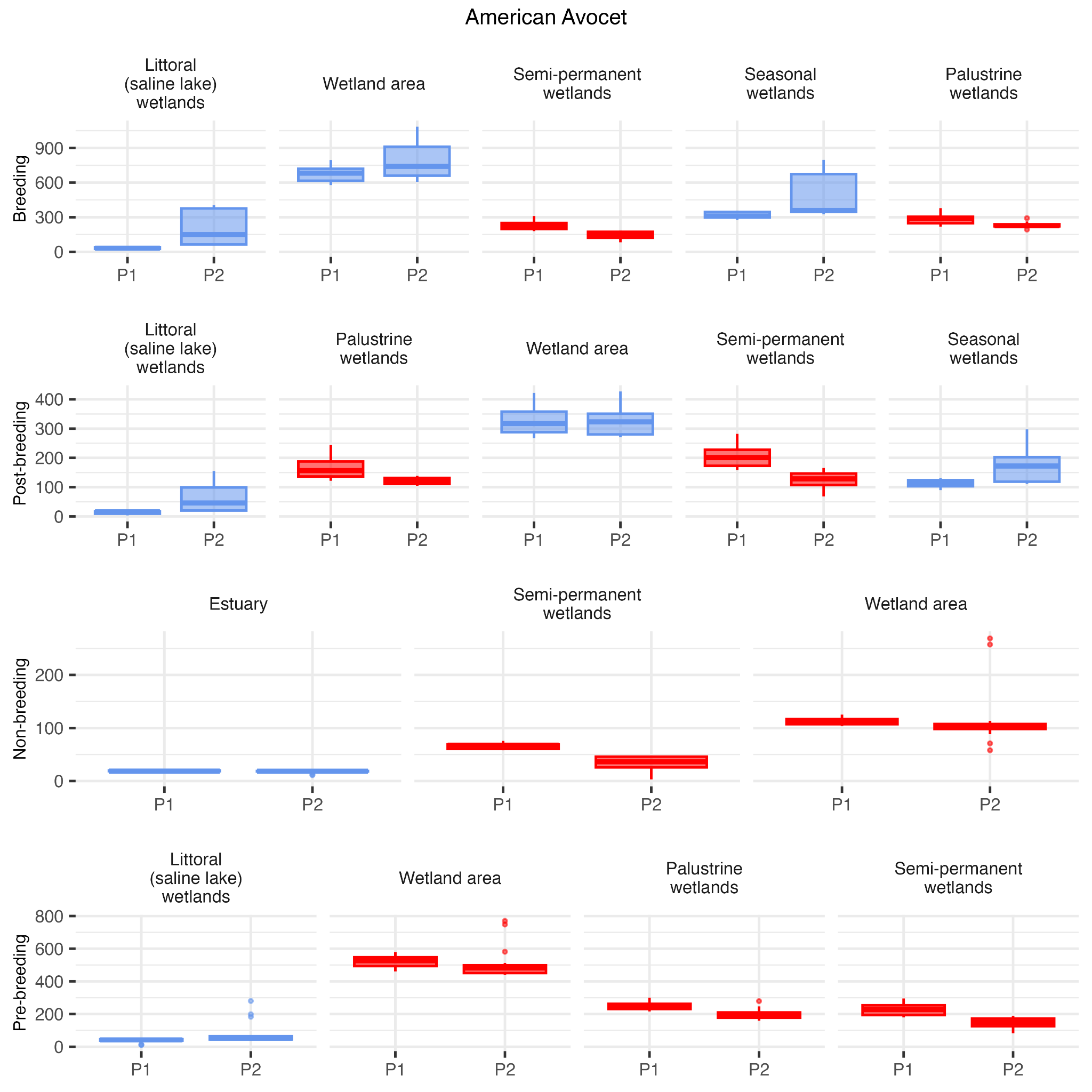


Figure S36. American avocet—explanation of results as referenced previously.


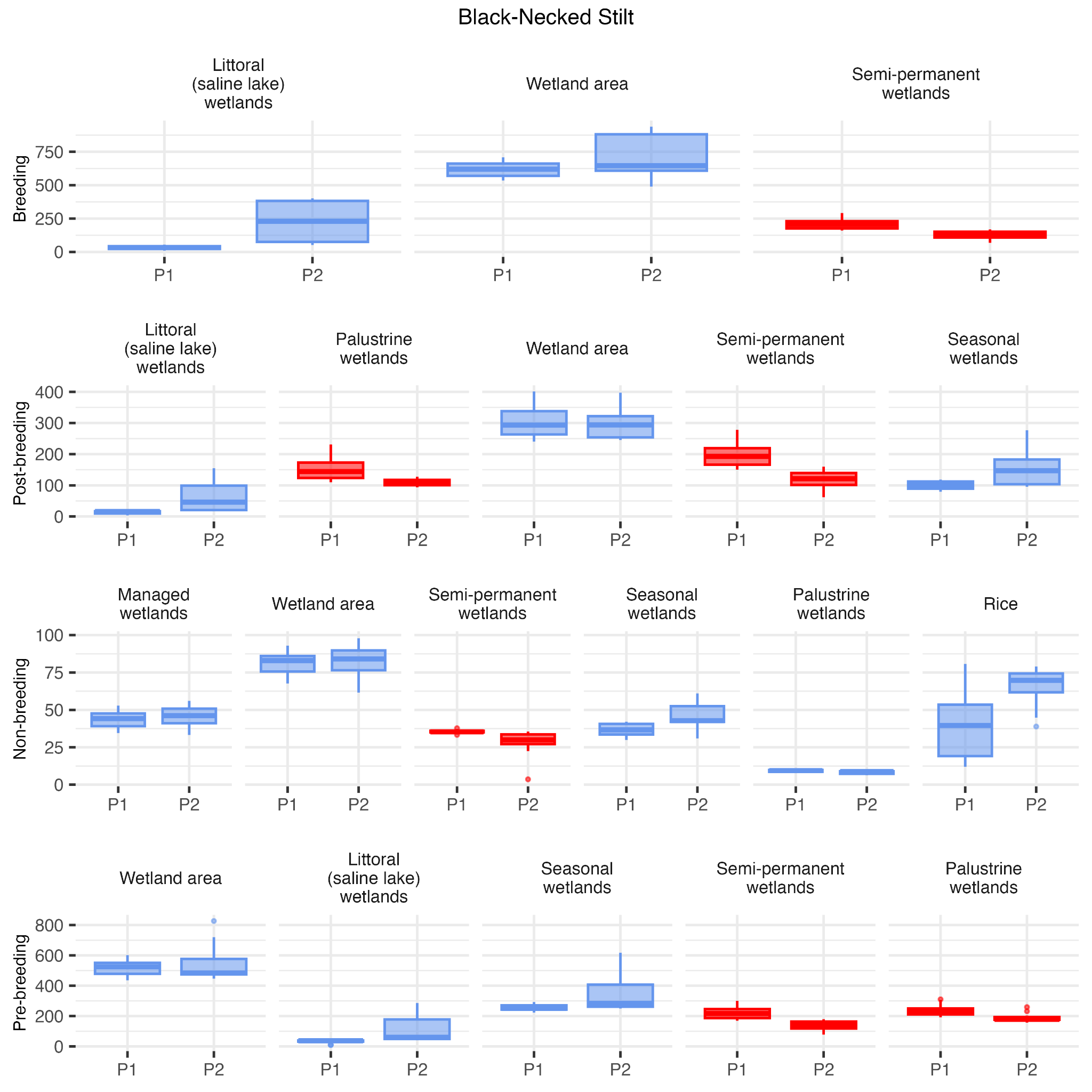


Figure S37. Black-necked stilt—explanation of results as referenced previously.


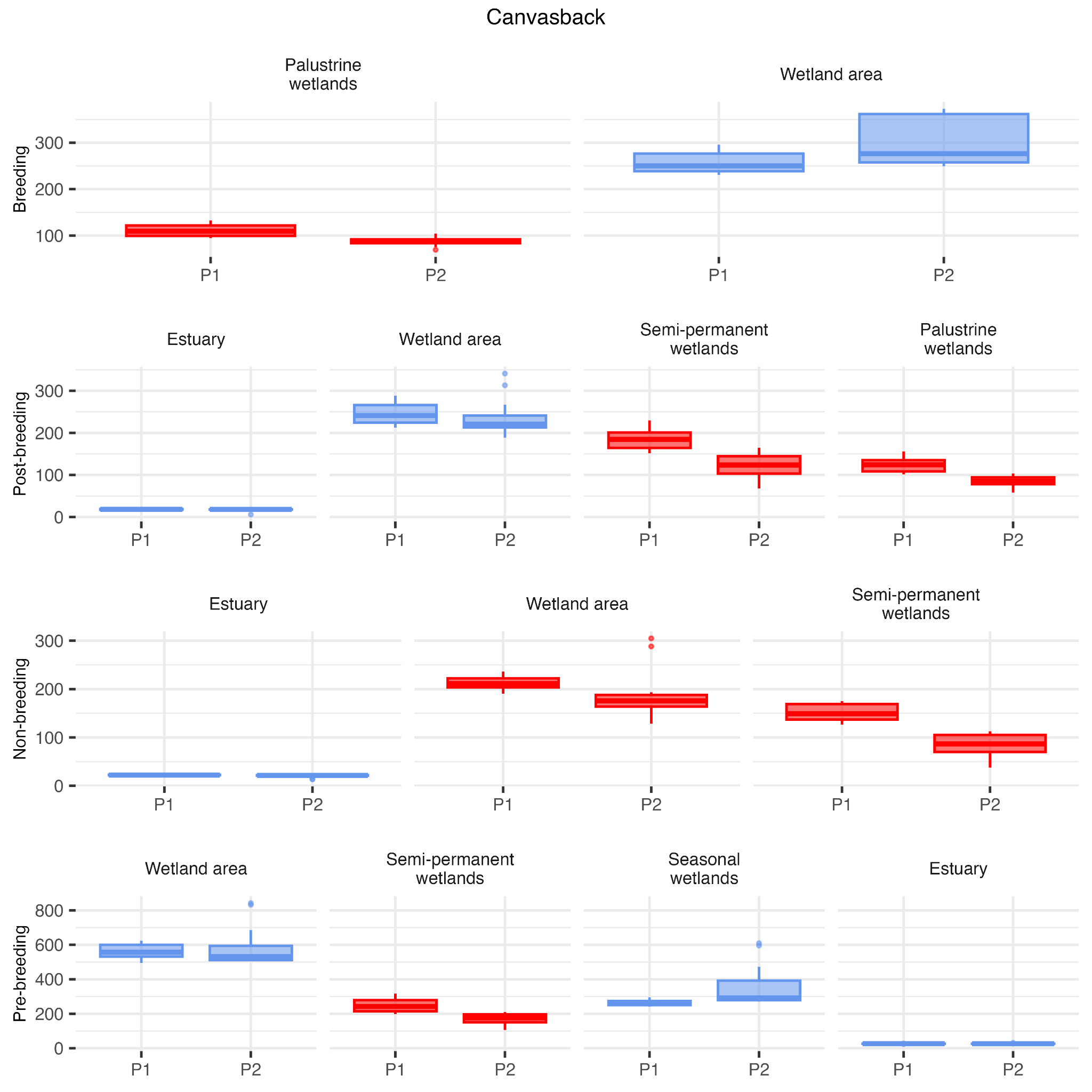


Figure S38. Canvasback—explanation of results as referenced previously.


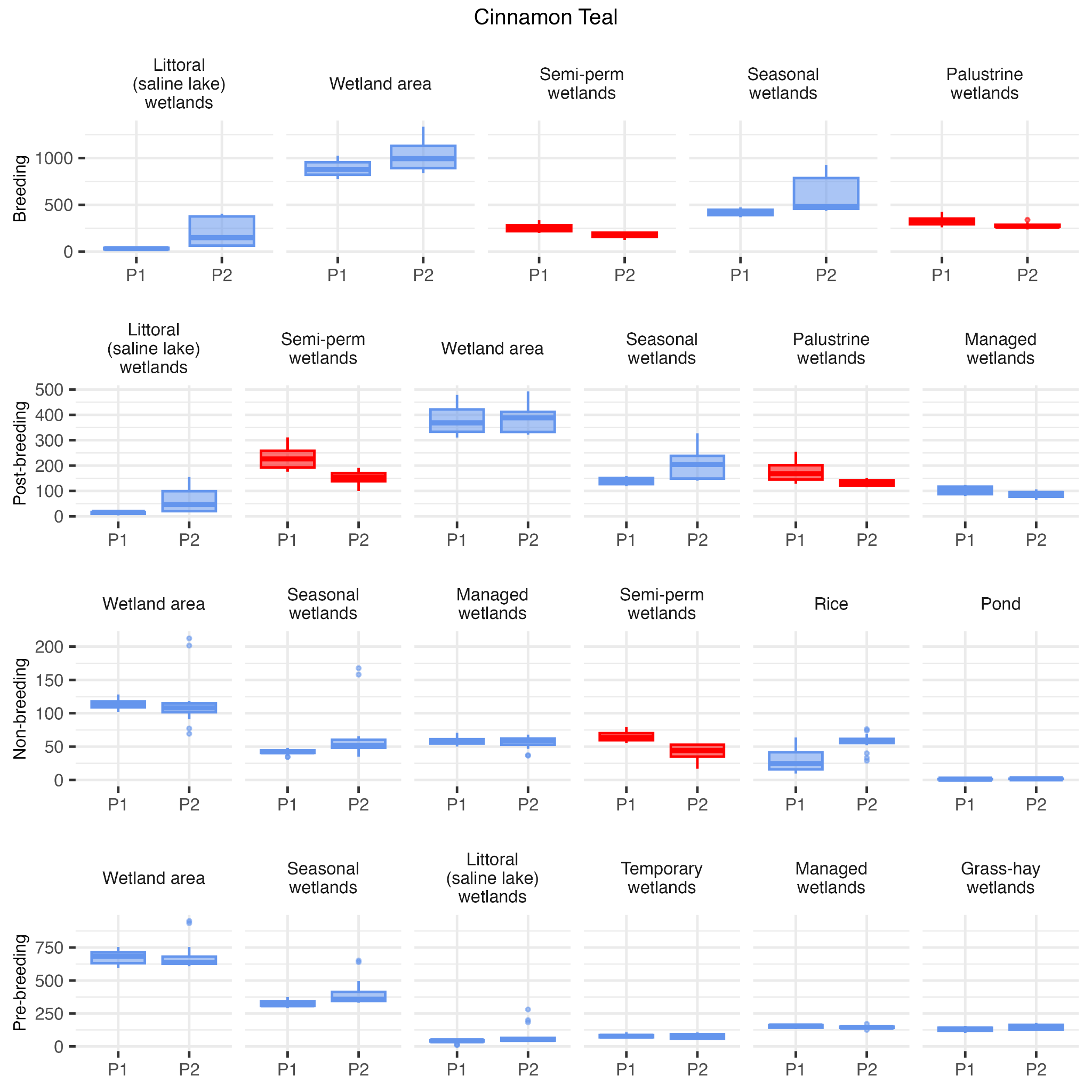


Figure S39. Cinnamon teal—explanation of results as referenced previously.


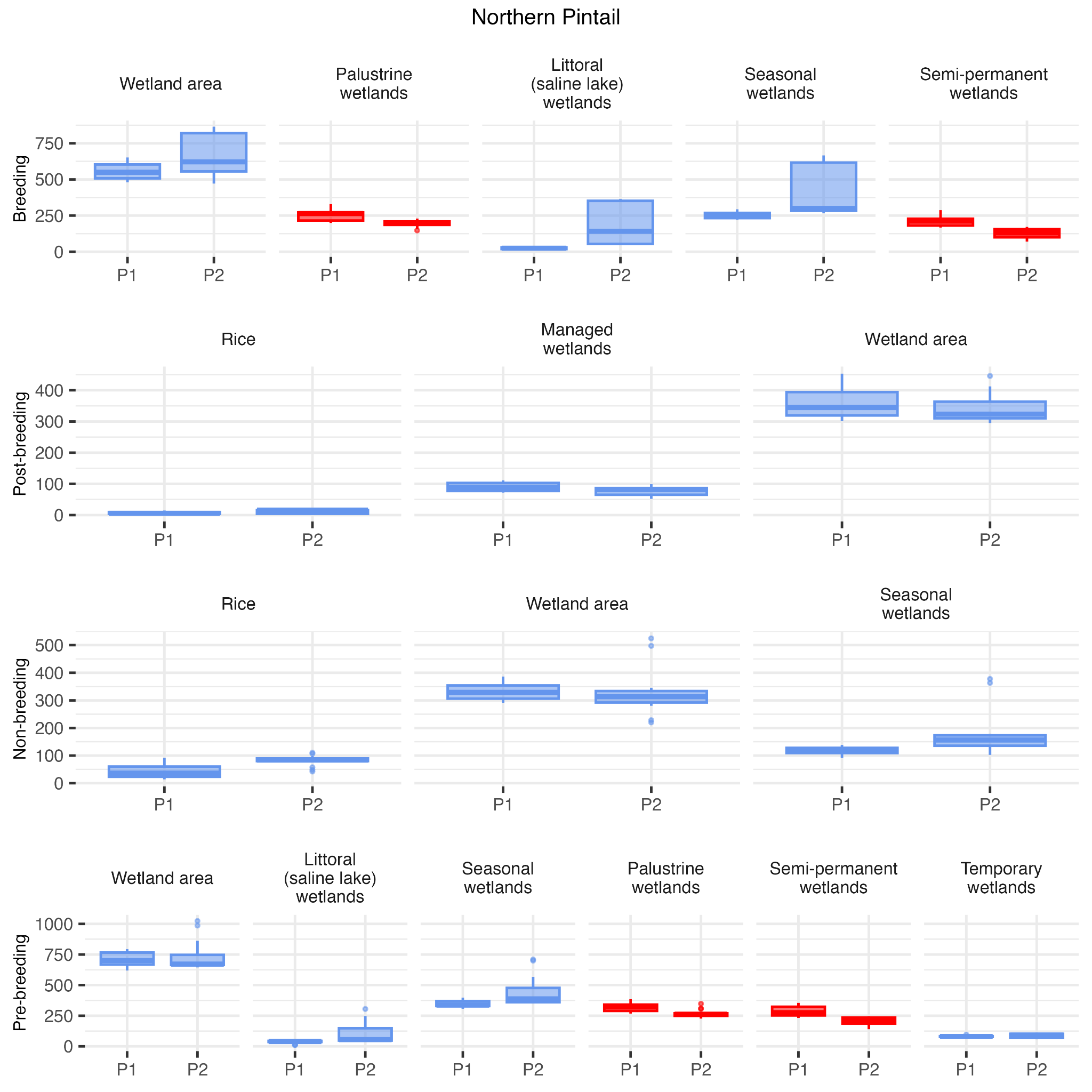


Figure S40. Northern pintail—explanation of results as referenced previously.


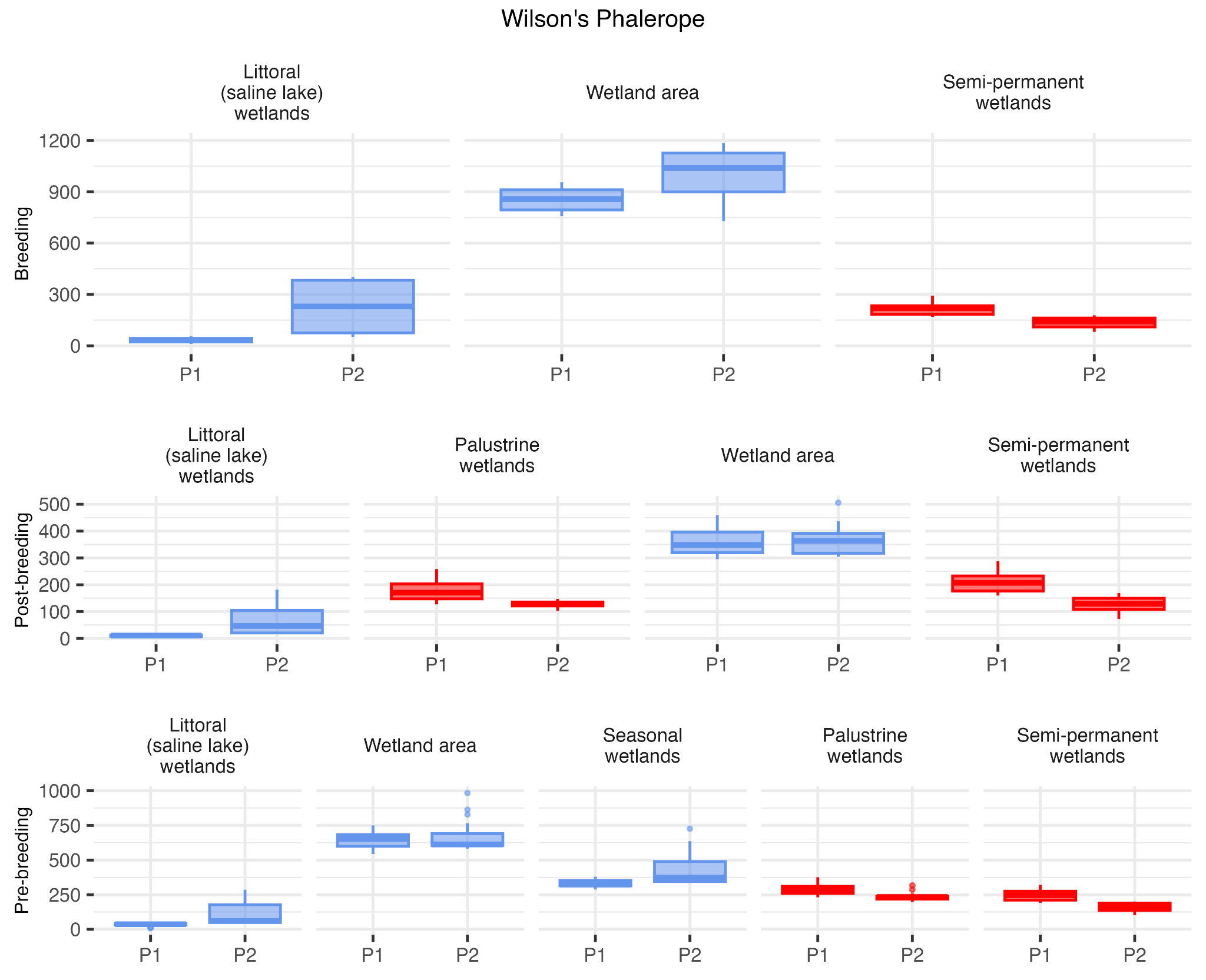


Figure S41. Wilson’s phalarope—explanation of results as referenced previously.

1.5 Mean wetland-waterbird surface water change

Tables S1-S6 measure the change in surface water area (kha) for wetland features structuring the abundance of American avocet, black-necked stilt, canvasback, cinnamon teal, northern pintail, and Wilson’s Phalarope in the western U.S. Wetlands features are presented in descending order of RandomForestSRC variable importance score for each life-history period. Low-scoring features with limited influence on bird abundance were excluded. Wetland change was measured as mean annual differences in surface water area (ha) between P1 (1984-2003) and P2 (2004-2023).

Table S1.American avocet—explanation of results as referenced previously.

| Wetland class | P1(1984-2003) | P2(2004-2023) | Difference |
| --- | --- | --- | --- |
| Breeding |  |  |  |
| Littoral (saline lake) wetlands | 31,429 | 195,532 | 522% |
| Wetland area | 674,261 | 785,552 | 17% |
| Semi-permanent wetlands | 227,574 | 145,201 | -36% |
| Seasonal wetlands | 317,747 | 469,373 | 48% |
| Palustrine wetlands | 282,835 | 231,060 | -18% |
| Post-breeding migration |  |  |  |
| Littoral (saline lake) wetlands | 12,985 | 62,391 | 380% |
| Palustrine wetlands | 166,900 | 121,413 | -27% |
| Wetland area | 327,357 | 322,970 | -1% |
| Semi-permanent wetlands | 203,835 | 125,214 | -39% |
| Seasonal wetlands | 112,821 | 169,956 | 51% |
| Non-breeding |  |  |  |
| Estuary | 19,223 | 17,576 | -9% |
| Semi-permanent wetlands | 66,123 | 34,600 | -48% |
| Wetland area | 112,237 | 117,971 | 5% |
| Pre-breeding migration |  |  |  |
| Littoral (saline lake) wetlands | 37,620 | 81,953 | 118% |
| Wetland area | 522,577 | 511,671 | -2% |
| Palustrine wetlands | 248,832 | 199,030 | -20% |
| Semi-permanent wetlands | 224,374 | 144,901 | -35% |

Table S2. Black-necked stilt—explanation of results as referenced previously.

| Wetland class | P1(1984-2003) | P2(2004-2023) | Difference |
| --- | --- | --- | --- |
| Breeding |  |  |  |
| Littoral (saline lake) wetlands | 32,265 | 228,670 | 609% |
| Wetland area | 616,682 | 718,659 | 17% |
| Semi-permanent wetlands | 207,526 | 128,289 | -38% |
| Post-breeding migration |  |  |  |
| Littoral (saline lake) wetlands | 12,983 | 62,390 | 381% |
| Palustrine wetlands | 154,389 | 109,799 | -29% |
| Wetland area | 304,679 | 295,131 | -3% |
| Semi-permanent wetlands | 196,322 | 118,888 | -39% |
| Seasonal wetlands | 100,227 | 150,835 | 50% |
| Non-breeding |  |  |  |
| Managed wetlands | 43,440 | 45,185 | 4% |
| Wetland area | 80,915 | 82,086 | 1% |
| Semi-permanent wetlands | 35,411 | 28,666 | -19% |
| Seasonal wetlands | 36,674 | 45,271 | 23% |
| Palustrine wetlands | 9,224 | 8,219 | -11% |
| Rice | 38,478 | 65,766 | 71% |
| Pre-breeding migration |  |  |  |
| Wetland area | 518,479 | 540,744 | 4% |
| Littoral (saline lake) wetlands | 33,109 | 111,614 | 237% |
| Seasonal wetlands | 254,268 | 344,141 | 35% |
| Semi-permanent wetlands | 219,210 | 138,046 | -37% |
| Palustrine wetlands | 235,522 | 188,028 | -20% |

Table S3. Canvasback—explanation of results as referenced previously.

| Wetland class | 1984-2003 | 2004-2023 | Difference |
| --- | --- | --- | --- |
| Breeding |  |  |  |
| Palustrine wetlands | 111,261 | 88,163 | -21% |
| Wetland area | 258,157 | 302,262 | 17% |
| Post-breeding migration |  |  |  |
| Estuary | 18,278 | 17,381 | -5% |
| Wetland area | 246,082 | 234,524 | -5% |
| Semi-permanent wetlands | 183,199 | 122,965 | -33% |
| Palustrine wetlands | 124,230 | 84,450 | -32% |
| Non-breeding |  |  |  |
| Estuary | 22,362 | 20,483 | -8% |
| Wetland area | 213,593 | 185,220 | -13% |
| Semi-permanent wetlands | 150,713 | 85,204 | -43% |
| Pre-breeding migration |  |  |  |
| Wetland area | 563,125 | 583,620 | 4% |
| Semi-permanent wetlands | 246,414 | 170,129 | -31% |
| Seasonal wetlands | 263,170 | 354,373 | 35% |
| Estuary | 26,574 | 26,962 | 1% |

Table S4.Cinnamon teal—explanation of results as referenced previously.

| Wetland classes | 1984-2003 | 2004-2023 | Difference |
| --- | --- | --- | --- |
| Breeding |  |  |  |
| Littoral (saline lake) wetlands | 31,354 | 195,512 | 524% |
| Wetland area | 892,665 | 1,022,908 | 15% |
| Semi-perm wetlands | 253,356 | 176,993 | -30% |
| Seasonal wetlands | 417,692 | 587,837 | 41% |
| Palustrine wetlands | 328,922 | 277,248 | -16% |
| Post-breeding migration |  |  |  |
| Littoral (saline lake) wetlands | 12,988 | 62,400 | 380% |
| Semi-perm wetlands | 227,224 | 151,916 | -33% |
| Wetland area | 380,089 | 385,003 | 1% |
| Seasonal wetlands | 138,892 | 201,057 | 45% |
| Palustrine wetlands | 177,203 | 132,344 | -25% |
| Managed wetlands | 101,460 | 86,969 | -14% |
| Non-breeding |  |  |  |
| Wetland area | 114,357 | 115,672 | 1% |
| Seasonal wetlands | 41,706 | 65,216 | 56% |
| Managed wetlands | 58,731 | 56,134 | -4% |
| Semi-perm wetlands | 65,273 | 42,889 | -34% |
| Rice | 28,948 | 56,684 | 96% |
| Pond | 1,310 | 1,683 | 28% |
| Pre-breeding migration |  |  |  |
| Wetland area | 676,263 | 682,235 | 1% |
| Seasonal wetlands | 323,354 | 403,772 | 25% |
| Littoral (saline lake) wetlands | 37,651 | 82,000 | 118% |
| Temporary wetlands | 79,373 | 78,720 | -1% |
| Managed wetlands | 152,167 | 145,501 | -4% |
| Grass-hay wetlands | 130,452 | 142,520 | 9% |

Table S5. Northern pintail—explanation of results as referenced previously.

| Wetland Class | 1984-2003 | 2004-2023 | Difference |
| --- | --- | --- | --- |
| Breeding |  |  |  |
| Wetland area | 558,496 | 664,739 | 19% |
| Palustrine wetlands | 251,107 | 195,831 | -22% |
| Littoral (saline lake) wetlands | 23,177 | 189,245 | 717% |
| Seasonal wetlands | 253,898 | 398,944 | 57% |
| Semi-permanent wetlands | 209,938 | 125,546 | -40% |
| Post-breeding migration |  |  |  |
| Rice | 7,447 | 11,844 | 59% |
| Managed wetlands | 90,222 | 76,046 | -16% |
| Wetland area | 359,855 | 342,601 | -5% |
| Non-breeding |  |  |  |
| Rice | 42,227 | 81,752 | 94% |
| Wetland area | 333,091 | 326,690 | -2% |
| Seasonal wetlands | 117,595 | 176,901 | 50% |
| Pre-breeding migration |  |  |  |
| Wetland area | 713,119 | 734,055 | 3% |
| Littoral (saline lake) wetlands | 35,849 | 102,024 | 185% |
| Seasonal wetlands | 346,419 | 443,211 | 28% |
| Palustrine wetlands | 318,766 | 265,541 | -17% |
| Semi-permanent wetlands | 285,756 | 205,741 | -28% |
| Temporary wetlands | 80,944 | 85,103 | 5% |

Table S6. Wilson’s phalarope—explanation of results as referenced previously.

| Wetland class | 1984-2003 | 2004-2023 | Difference |
| --- | --- | --- | --- |
| Breeding |  |  |  |
| Littoral (saline lake) wetlands | 32,196 | 228,641 | 610% |
| Wetland area | 850,719 | 1,006,064 | 18% |
| Semi-permanent wetlands | 214,309 | 133,953 | -37% |
| Post-breeding migration |  |  |  |
| Littoral (saline lake) wetlands | 10,275 | 63,861 | 522% |
| Palustrine wetlands | 179,361 | 127,324 | -29% |
| Wetland area | 360,795 | 364,768 | 1% |
| Semi-permanent wetlands | 207,937 | 127,405 | -39% |
| Pre-breeding migration |  |  |  |
| Littoral (saline lake) wetlands | 33,049 | 111,609 | 238% |
| Wetland area | 644,375 | 674,282 | 5% |
| Seasonal wetlands | 329,628 | 431,834 | 31% |
| Palustrine wetlands | 287,967 | 235,583 | -18% |
| Semi-permanent wetlands | 244,805 | 158,498 | -35% |
