# Supplementary material for: eBird Data Highlight Shifts in Wetland Resources Structuring Waterfowl and Shorebird Abundance
Source: Ecol Evol. 2026 Feb 17;16(2):e73061. doi: 10.1002/ece3.73061 (PMC12913221; doi:10.1002/ece3.73061)
Supplement: Supplementary file 2 — Appendix S2: ece373061‐sup‐0002‐AppendixS2.docx. [file ECE3-16-e73061-s002.docx]

Appendix S2

1.0 Remote sensing methods for wetland surface water trends

Following the methods outlined by Donnelly et al. [(2022)](https://paperpile.com/c/DF1J8W/koC0y/?noauthor=1), wetland surface water conditions were measured monthly as a 15-year mean (2008-2022) to overlap the period of eBird abundance data. Conditions were measured using constrained spectral mixture analysis [(SMA; Adams and Gillespie, 2006)](https://paperpile.com/c/DF1J8W/W38LE/?prefix=SMA%3B) applied to Landsat 5 Thematic Mapper and Landsat 8/9 Operational Land Imager satellite imagery. This approach allowed proportional estimations of water contained within a continuous 30×30 m pixel grid [(Halabisky et al., 2016; Jin et al., 2017)](https://paperpile.com/c/DF1J8W/yuNnM+BN56o) and provided an accurate account of flooding when detectability was reduced due to interspersion of emergent vegetation, shallow, or turbid water (DeVries et al., 2017), characteristics common to seasonal wetlands in semi-arid regions (Jolly et al., 2008). Because these conditions can partially mask areas covered with water [(DeVries et al., 2017; sensu Donnelly et al., 2019)](https://paperpile.com/c/DF1J8W/YUpVg+D88mt/?prefix=sensu,), we considered pixels fully inundated when water was present. Pixels containing <15% surface water were omitted from summaries to minimize the overestimation of surface water area. Satellite data used for SMA were formatted by binning individual Landsat scenes by month and averaging results into twelve composite images. Areas containing cloud, cloud shadow, snow, and ice were masked using the Landsat CFMask band [(Foga et al., 2017)](https://paperpile.com/c/DF1J8W/HTAia). All unmasked pixels in Landsat 30 m visible, near-infrared, and short-wave infrared bands were incorporated into SMA except for the Landsat 8 coastal aerosol band. All Landsat data were preprocessed to level-2 surface reflectance [(USGS 2017a, USGS 2017b)](https://paperpile.com/c/DF1J8W/rl8iq+tjEve/?prefix=USGS,USGS&noauthor=1,1). Surface water was not measured in 2012 due to poor-quality satellite imagery.

Training data for SMA were extracted from satellite imagery as spectral end-members unique to individual composite images classified. Non-water spectral end-members were used to account for spectral diversity within satellite images as a means to isolate surface water pixels from other land cover types. Training site locations represented homogeneous land cover types for water, herbaceous vegetation, shrubs, trees, and bare soil. End-member training locations were spatially stratified to ensure data were representative of diverse environmental conditions. Spectral end-members for water were collected using image masks generated from the 99th percentile normalized difference water index values [(McFeeters, 1996)](https://paperpile.com/c/DF1J8W/aNEgL). Endmember generation was constrained to large deepwater lakes and reservoirs (n = 34) within the study area to avoid false positives from non-water dark body objects (i.e., topographic shadows). A similar masking approach was applied to collect herbaceous vegetation end-members using 95th percentile normalized difference vegetation indices [(Box et al., 1989)](https://paperpile.com/c/DF1J8W/5VtkB). Sampling was constrained to sites with herbaceous cover greater than 80% using masks generated through fractional estimations of functional plant groups derived from Rangeland Analysis Platform data [(Allred et al., 2021)](https://paperpile.com/c/DF1J8W/JIBMY). This approach was repeated for shrub and tree end-member generation. Because spectral mixture analysis requires minimal training data [(Adams and Gillespie, 2006)](https://paperpile.com/c/DF1J8W/W38LE), mask generation for herbaceous, shrub, and tree end-members was constrained to select locations (n = 34). Spectral end-members for bare soil were derived from static plots identified using on-screen interpretation of natural and near-infrared 0.6-meter and 0.3-meter resolution National Agriculture Imagery Program data [(USDA 2024)](https://paperpile.com/c/DF1J8W/oYV07/?prefix=USDA&noauthor=1). Plots (n = 34, 100x100 meters) were representative of homogenous bare soil conditions. Data accuracy was estimated to be 93-98% by comparison to previous work and identical methods used by Donnelly et al. (in press) that overlapped the entirety of our study area. This estimate was comparable to similar time‐series wetland inundation studies using Landsat data [(Jin et al., 2017)](https://paperpile.com/c/DF1J8W/BN56o).

Wetland hydroperiods were calculated using monthly SMA surface water estimates by totaling the duration of time individual pixels were flooded. Wetlands were classified as ‘temporary’ (flooded < 2 months), ‘seasonal’ (flooded > 2 and < 9 months), or ‘semi-permanent’ (flooded > 8 months) using standards similar to Cowardin et al. [(1979)](https://paperpile.com/c/DF1J8W/SCPkU/?noauthor=1). We used hydroperiod data and raster masking functions to classify surface water by masking the hydroperiod layer with monthly surface water estimates. The outcomes delineated monthly temporary, seasonal, and semi-permanent wetland extents representative of mean conditions from 2008 to 2022. All remote sensing and raster‐based analyses were conducted using Google Earth Engine, a cloud‐based geospatial processing platform [(Gorelick et al., 2017)](https://paperpile.com/c/DF1J8W/SJ9DK).

References

[Adams, J.B., Gillespie, A.R., 2006. Spectral-mixture analysis. In: Remote Sensing of Landscapes with Spectral Images: A Physical Modeling Approach. Cambridge University Press, pp. 126–165.](http://paperpile.com/b/DF1J8W/W38LE)

[Allred, B.W., Bestelmeyer, B.T., Boyd, C.S., Brown, C., Davies, K.W., Duniway, M.C., Ellsworth, L.M., Erickson, T.A., Fuhlendorf, S.D., Griffiths, T.V., Jansen, V., Jones, M.O., Karl, J., Knight, A., Maestas, J.D., Maynard, J.J., McCord, S.E., Naugle, D.E., Starns, H.D., Twidwell, D., Uden, D.R., 2021. Improving Landsat predictions of rangeland fractional cover with multitask learning and uncertainty. Methods Ecol. Evol. 12, 841– 849.](http://paperpile.com/b/DF1J8W/JIBMY)

[Box, E.O., Holben, B.N., Kalb, V., 1989. Accuracy of the AVHRR vegetation index as a predictor of biomass, primary productivity and net CO2 flux. Vegetatio 80, 71–89.](http://paperpile.com/b/DF1J8W/5VtkB)

[Cowardin, L.M., Carter, F.C., Golet, E.T., 1979. Classification of wetlands and deepwater habitats of the United States. United States Department of the Interior, Fish and Wildlife Service, Washington, DC, USA.](http://paperpile.com/b/DF1J8W/SCPkU)

[DeVries, B., Huang, C., Lang, M.W., Jones, J.W., Huang, W., Creed, I.F., Carroll, M.L., 2017. Automated Quantification of Surface Water Inundation in Wetlands Using Optical Satellite Imagery. Remote Sensing 9, 807.](http://paperpile.com/b/DF1J8W/D88mt)

[Donnelly, J.P., Moore, J.N., Casazza, M.L., Coons, S.P., 2022. Functional Wetland Loss Drives Emerging Risks to Waterbird Migration Networks. Frontiers in Ecology and Evolution 10, 1–18.](http://paperpile.com/b/DF1J8W/koC0y)

[Donnelly, J.P., Naugle, D.E., Collins, D.P., Dugger, B.D., Allred, B.W., Tack, J.D., Dreitz, V.J., 2019. Synchronizing conservation to seasonal wetland hydrology and waterbird migration in semi‐arid landscapes. Ecosphere 10, 1–12.](http://paperpile.com/b/DF1J8W/YUpVg)

[Foga, S., Scaramuzza, P.L., Guo, S., Zhu, Z., Dilley, R.D., Beckmann, T., Schmidt, G.L., Dwyer, J.L., Joseph Hughes, M., Laue, B., 2017. Cloud detection algorithm comparison and validation for operational Landsat data products. Remote Sens. Environ. 194, 379–390.](http://paperpile.com/b/DF1J8W/HTAia)

[Gorelick, N., Hancher, M., Dixon, M., Ilyushchenko, S., Thau, D., Moore, R., 2017. Google Earth Engine: Planetary-scale geospatial analysis for everyone. Remote Sens. Environ. 202, 18–27.](http://paperpile.com/b/DF1J8W/SJ9DK)

[Halabisky, M., Moskal, L.M., Gillespie, A., Hannam, M., 2016. Reconstructing semi-arid wetland surface water dynamics through spectral mixture analysis of a time series of Landsat satellite images (1984–2011). Remote Sens. Environ. 177, 171–183.](http://paperpile.com/b/DF1J8W/yuNnM)

[Jin, H., Huang, C., Lang, M.W., Yeo, I.-Y., Stehman, S.V., 2017. Monitoring of wetland inundation dynamics in the Delmarva Peninsula using Landsat time-series imagery from 1985 to 2011. Remote Sens. Environ. 190, 26–41.](http://paperpile.com/b/DF1J8W/BN56o)

[McFeeters, S.K., 1996. The use of the Normalized Difference Water Index (NDWI) in the delineation of open water features. Int. J. Remote Sens. 17, 1425–1432.](http://paperpile.com/b/DF1J8W/aNEgL)

[[USDA] U.S. Department of Agriculture, Farm Production and Conservation - Business Center, Geospatial Enterprise Operations, 2024. National Agriculture Imagery Program (NAIP) [WWW Document]. NAIP GeoHub. URL](http://paperpile.com/b/DF1J8W/oYV07) <https://naip-usdaonline.hub.arcgis.com/> [(accessed 2.24).](http://paperpile.com/b/DF1J8W/oYV07)

[[USGS] United States Department of Interior, Geological Survey, 2017a. LANDSAT 4-7 Surface Reflectance (LEDAPS) Product ( No. version 7.9). United States Department of the Interior, Geological Survey.](http://paperpile.com/b/DF1J8W/rl8iq)

[[USGS] United States Department of Interior, Geological Survey, 2017b. LANDSAT 8 Surface Reflectance Code (LASRC) Product ( No. version 4.0). United States Department of the Interior, Geological Survey.](http://paperpile.com/b/DF1J8W/tjEve)
